# Toward quantitative and reproducible clinical use of OCT-Angiography

**DOI:** 10.1371/journal.pone.0197588

**Published:** 2018-07-06

**Authors:** Ikrame Douma, David Rousseau, Rebecca Sallit, Laurent Kodjikian, Philippe Denis

**Affiliations:** 1 LARIS, UMR INRA IRHS, Université d’Angers, 62 Avenue Notre Dame du Lac, 49000 Angers, France; 2 Department of Ophthalmology, Croix-Rousse University Hospital, Hospices Civils de Lyon, University of Lyon I, Lyon, France; Charite Universitatsmedizin Berlin, GERMANY

## Abstract

Optical coherence tomography angiography (OCT-A) is an ophthalmic imaging technique which has recently been introduced to clinical use. OCT-A provides visualization of the retinal vascularization in three dimensions, without injection of contrast agents. OCT-A could thus replace the current standard of opthalmic imaging, which is 2D only and requires contrast agents. However, quantitative studies remain to be carried out to assess the full potential of OCT-A. In this context, the present work proposes a methodology to perform OCT-A in a more reproducible and precise way. We introduce a procedure to automatically extract the area of interest in avascular regions, which we demonstrate on various avascular areas with a focus on the optic nerve extracted in 2-dimensional images for a selected depth. We then study the repeatability of OCT-A with our segmentation technique when implemented on various clinical devices. For illustration, we apply this segmentation to healthy control group and to patients presenting different stages of glaucoma, a disease of clinical interest. The variability observed between these two cohorts compares favorably to the variability due to instrumental limitations or the segmentation algorithm. Our results thus constitute a significant step toward a more quantitative use of OCT-A in a clinical context.

## Introduction

Optical coherence tomography angiography (OCT-A) is an emerging imaging method that allows visualization of retinal vascularization without injection of contrast agents [1]. As is the case with for any novel imaging technique, a comparison of instrumentation issues and clinical tests are necessary before consideration of the approach as a clinical tool of reference. Various open questions remain such as the most precise way to quantify structures of interest (the vascular density in our case), the reproducibility of acquisitions and how these uncertainties limit the discrimination capability of the instrumentation in definitive clinical studies.

Currently, the standard technique used in clinical practice for retinal vascular inspection is fluorescein angiography (FA), which requires injection of a contrast agent to visualize retinal vessels. FA produces quantitative information in two dimensions on the velocity of blood flow and the presence of vessel extravasations. Although minimal, there is a risk of anaphylaxis with fluororescein angiography that is absent with OCT A. OCT-A allows to obtain high resolution three-dimensional images of the ocular vascularization. Furthermore, FA visualizes only the superficial capillary network whereas OCT-A can probe the vascular network over the full depth of the retina. Consequently, OCT-A seems very promising for the estimation of parameters such as blood flow and vascular density. However, the reproducibility of the quantification of these parameters and the feasibility of automatic region-of-interest selection have not been established so far.

Analyses of 2D vascular networks have been described in numerous publications [2–25]. Parameters such as the vascular density in terms of the skeleton of vessels, diameter of vessels, perimeter and tortuosity have been demonstrated to be correlated with lesions. These parameters serve to understand physio-pathological mechanisms of diseases. Many studies have therefore been carried out with OCT-A to highlight the contrasts observed for different ophthalmic pathologies such as vascular defects in diabetes [13–16], retinal venous occlusion [17–19] neovascularization in age-related macular degeneration [20, 21], or glaucoma [1, 6–10, 22–24]. In general, a region of interest was defined, and an analysis of vascular density was then performed, for example in the peripapillary region [6, 7, 10–12]. These studies suggest that the segmentation of the peripapillary region around the optic nerve is more challenging than for the macula, as the optic nerve exhibits considerable disparities in terms of size and shape. It can be circular, ellipsoidal, or completely irregular. Some authors circumvented the problem by using another imaging method to define the margins of the optic nerve, for example, a reflectance image of the optical nerve [22], while others rely on a constant-size disk or ellipse to analyze the peripapillary region [6, 11, 12, 24]. However, several limitations appear with this approach since the analyzed region is never a pure geometric disk and furthermore the size of the disk remains to be determined. We therefore believe it is important to provide accurate and automated generic methods to select the avascular region of interest without depending on prior assumptions about its shape and size.

It is furthermore important to be noted that there is an increasing number a OCT-A devices available, and others are still currently in development stage. These devices differ from each other by their algorithm, scan-acquisition frequency, wavelength, eyetracking system or resolution. The available comparisons between the various devices are currently rather limited; the most recent work in this spirit is [26], which proposes a mainly qualitative comparison of 4 OCT-A devices, based on artifacts produced by the different instruments. Currently, the fluctuation of measured parameters due to the devices themselves, which has to be taken into account when trying yo assess the evolution of a disease, remains poorly understood.

The main objectives of this study are therefore to establish an automated method for the segmentation of avascular regions of interest in OCT-A, to determine the reproducibility of the acquisitions, and to compare the performance of different devices, based on this segmentation method when applied to an ophthalmic pathology (glaucoma) around the optic nerve. To illustrate the interest of our technique, we tested it on patients suffering from 4 different stages of primary open angle glaucoma.

## Materials and methods

### Cohort

Primary open angle glaucoma (POAG) is defined as a progressive, chronic optic neuropathy characterised by changes in the optic disc that are associated with irreversible defects in the visual field. The data of this study were obtained from the consultation of ophthalmology of the Croix Rousse Hospital, between February, 1 2017, and June, 31 2017. We included patients with POAG based on the following criteria: open angle in gonioscopy, decreased retinal nerve fiber thickness RNFL on the OCT-SD. The OCT apparatus used in this study was a Cirrus HD-OCT 5000 (Carl Zeiss Meditec, Dublin, CA, USA). Finally, alterations of the visual field had to be present (a cluster of 3 or more points on the pattern deviation plot in an expected location of the visual field depressed below the 5% level, at least one of which was depressed below the 1% level). Control data were obtained from medical staff, free from any ophtalmological pathology. The controls necessarily had an intraocular pressure of less than 21 mmHg, a normal ophthalmic examination, and a normal RNFL procedure. The exclusion criteria common to both groups were the presence of a disorder of the media preventing the acquisition of OCT angiography, the presence of an ocular pathology other than glaucoma or cataract, any previous endo-ocular surgery other than cataract surgery. All patients underwent a comprehensive ophthalmologic examination: near and far visual acuity assessment, measurement of intraocular pressure, biomicroscopic examination, fundus, central corneal thickness measurement, gonioscopy, an OCT, an OCT angiography performed with Angioplex (Cirrus 5000 HD-OCT Angioplex, Carl Zeiss Meditec, Dublin, CA, USA). The study has been approved by the Ethics Committee of the French Society of Ophthalmology (IRB 00008855 Société Francaise d’Ophtalmologie IRB1). The data were used anonymously. None of the authors had access to potentially identifying information and none of the authors were the treating physicians.

### Segmentation of the region of interest (ROI)

**Fig 1:**
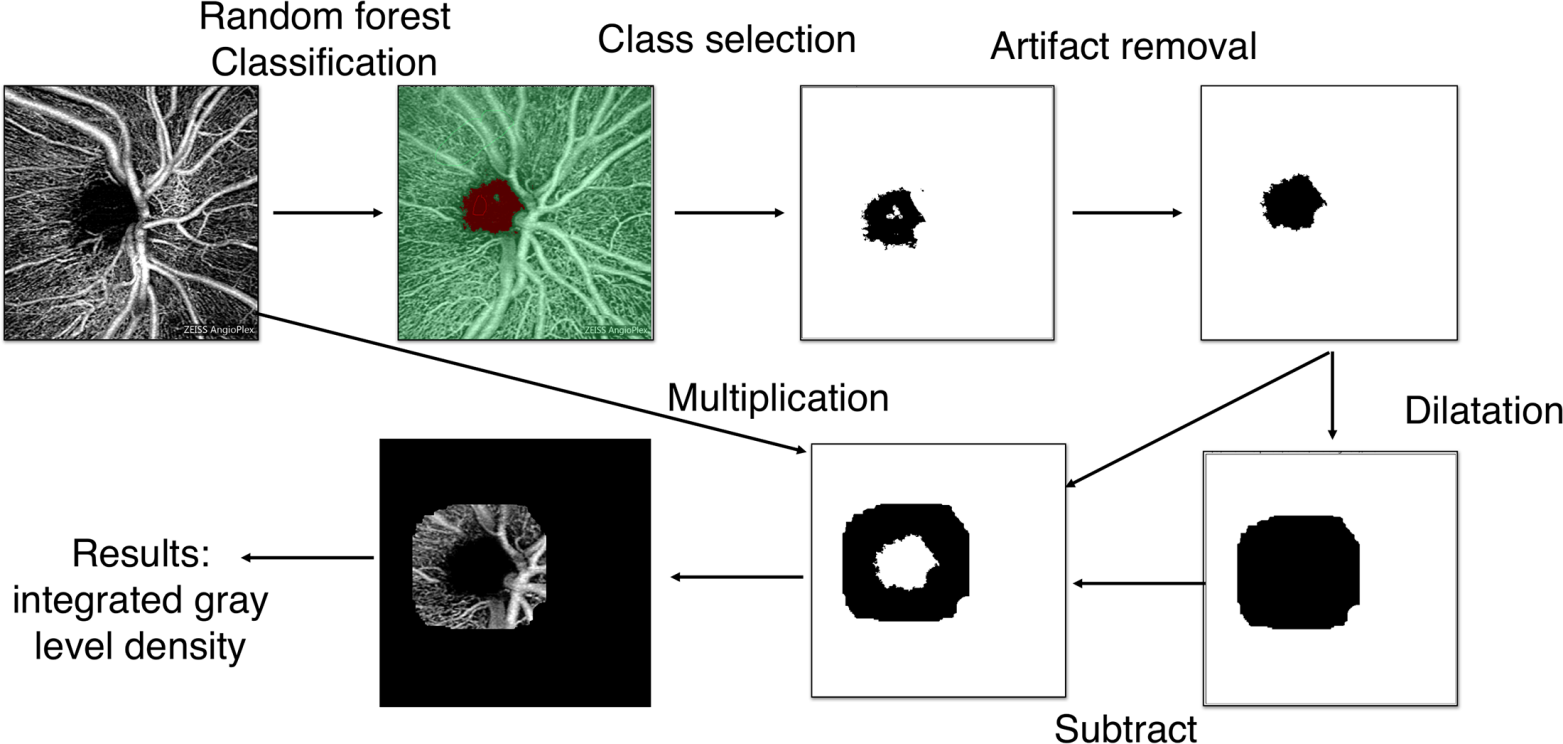
Image processing pipeline for vessel-density measurement in an automatically segmented region of interest.

We selected the peripapillary area for our study since this region is crucial region for the pathophysiology of chronic disease of the optic nerve [23, 24]. In order to isolate the optic nerve, we developed the image processing pipeline described in Fig 1, which uses a supervised classification, random forest algorithm: The segmentation is realized from a set of different features computed in a manually annotated area. Then the combination of these features is optimized by the random forest algorithm so as to fit with the classification requested by the annotator in the annotated area. The classification is pixel-based; however, the features can be computed with an indication of scale. In our case we included the range of scales corresponding to the expected size of vessels (from the tiniest to the biggest). This is an important point since this precaution avoids the identification of avascular region in the tiny interspace between the smallest visible vessels. This image processing pipeline is made available under the form of script, a Macro for the open-source FIJI software provided as supplementary data (See supporting information section).

### Multi-device comparison

To estimate the reproducibility of OCT-A device, we evaluated the repeatability of the pipeline defined in Fig 1. For this purpose, to avoid mixing biological variability and reproducibility due to instrumentation we acquired, on a single control, the OCT-A of the same eye 10 times, at the same position, every few seconds. OCT-A devices used for analysis (Table 1) were Zeiss Cirrus 5000 HD-OCT, AngioPlex (Zeiss Meditec. Inc, Germany), Spectralis OCT2 (Heidelberg Engineering. Germany), Plex Elit (Carl Zeiss Meditec, Inc., Dublin, USA), Topcon DRI swept-source OCT and Angiovue, RTVue XR Avanti (Optovue,Inc). Each instrument has its own characteristics. Angioplex operates using an OCT-microangiography complex algorithm (OMAG) that uses amplitude and phase OCT signal decorrelation to generate angiography images. This instrument produces A-scans at a frequency of 68KHz, using a FastTrac eye traking system to minimize artifacts. The Angioplex produces an image with an axial resolution of 5 *μm*. The Heidelberg OCT angiography prototype uses a full spectrum amplitude decorrelation algorithm (FS-ADA). This apparatus acquires A-scans at a frequency of 85kHz and produces an image with an axial resolution of 7 *μm*. PlexElit is a swept-source OCT-A that records 100,000 A-scans per sec and has a resolution of 6.3 *μm* [27]. Topcon operates using a blood flow detection, employs algorithm called OCT-A Ratio Analysis (OCTARA) and incorporates an eye-tracking system to reduce movement artifact. This system performs 100,000 A-scans per second and is a swept-source OCT using a wavelength of 1050 nm. Angiovue performs 70000 A-scans per second, and uses Split Spectrum amplitude decorelation (SSADA) to generate an image. The acquisition consists of 2 consecutive B-scans, taken at right angles to one another, of 304 × 304 A-scans, at the same position. For each device, we acquired a 3 × 3 mm scan of the optic nerve, from which we determined the mean gray value and its relative variation.

**Table 1:** Characteristics of the different OCT-A devices evaluated in this study.

	Zeiss Angioplex	Heidelberg Spectralis	Zeiss PlexElit	Topcon Triton	Optovue AngioVue
Technology	full spectrum spectral domain OMAG	full spectrum spectral domain	full spectrum swept source OMAG	full spectrum swept source	split spectrum spectral domain SSADA
Axial resolution [μm]	5	7	5.5	8	5
Mean duration of acquisition (3 × 3 mm) [sec]	13	201	12	15	28
Number of scans (3 × 3 mm)	350 horizontal scans	261 horizontal scans	300 horizontal scans	320 horizontal scans	304 horizontal scans and 304 vertical scans
Acquisition speed [scans/s]	68000	85000	100000	100000	70000
Eye tracker	FastTrack	TrueTrack	FastTrack	SMART TRACK	Two right angled OCT-A volumes scans performed correct motion artefacts

#### Reproducibility of the acquisition

For each device, we evaluated the mean gray value of the entire image and the standard deviation for ten images of the same eye acquired from a single patient. Finally, we computed the percentage of fluctuation of the vascular density estimated from the integrated gray-level density with the algorithm of Fig 1.

#### Reproducibility of measurement

Since the automatic segmentation pipeline of Fig 1 depends on manually annotated regions of a (single) training image, it was also necessary to evaluate the repeatability of this method of segmentation. We have, for the same eye, made ten distinct manual annotations in the training phase of the supervised classification. These annotations were positioned at different locations, but in the same anatomical regions.

#### Application

To further illustrate the significance of the reproducibility parameters discussed in the previous section, we studied images of patients followed for POAG from stage 1 to stage 4. The Hoddap-Parrish-Anderson glaucoma grading scale, based on visual field, distinguishes 6 groups of increasing gravity: Stage 0: normal visual field, stage 1 (early defect): MD ≥ −6.00 dB, stage 2 (moderate defect): ≥ −6.00 to −12.00 dB, stage 3 (advanced defect): ≥ −12.01 to −20.00 dB, stage 4 (severe defect): ≥ −20.00 dB and stage 5 (end stage disease): unable to perform VF [28]. We analysed both superficial and deep capillary networks. The selection of the depth (superficial and deep) was realized automatically by the OCT system by offsets of a fixed distance to the surface.

### Statistical analysis

Statistical analyses were performed using the R-Studio software package (www.r-project.org) with four adapted statistical tests. The Kolmogorov-Smirnov test was used to determine whether the two populations (healthy and unhealthy) followed the same distribution law. We furthermore used it to check whether the distribution of the average integrated density followed a normal distribution among healthy or diseased populations. The Fisher test was used to assess whether the samples were from the same population. More precisely, the null hypothesis corresponded to samples from the same population, whereas the alternative hypothesis implied that at least one distribution had a mean which deviated from the other average. If the p-value was greater than 0.05, the null hypothesis was retained. The Student test was used to compare averages (here, mean gray value of 2D images, at a determined depth, of healthy and unhealthy patients). Two conditions of application were verified for the use of this test: The populations must follow a normal law and the variance of the samples must be homogeneous. The Kruskal-Wallis test was used to compare several averages, when populations did not follow a normal law.

## Results

### Segmentation of the region of interest

We applied three sizes of binary masks in the peripapillary region in order to determine the discriminating potential of the imaging technique in a population of 20 healthy controls and 20 patients affected by glaucoma (Table 2). This evaluation was realized in both superficial and deep vascular networks.

**Table 2:** Relative difference of mean value of the peripapillary region of the superficial capillary network (SCN) and the deep capillary network (DCN) of 20 healthy controls and 20 unhealthy patients applied to 3 different sizes of the peripapillary region. The *p* value is given for the Fisher-test for significant difference between control group and patient population.

Diameter of peripapillary region	640 μm	920 μm	1960 μm
*p* value of SCN	0.199	0.71	0.065
*p* value of DCN	0.0028	0.0062	0.059

For the three tested mask sizes, the average integrated gray-levels in the peripapillary region were found to follow a normal distribution for both populations. These two normal distributions did not show any significant difference for the superficial network irrespective of the size of the peripapillary region. Concerning the deep vascular network, the analyses revealed a statistically significant difference for the peripapillary region of 640 μm. The two other tested masks did not produce significant results.

### Multi-device comparison

#### Reproducibility of the acquisition

**Fig 2:**
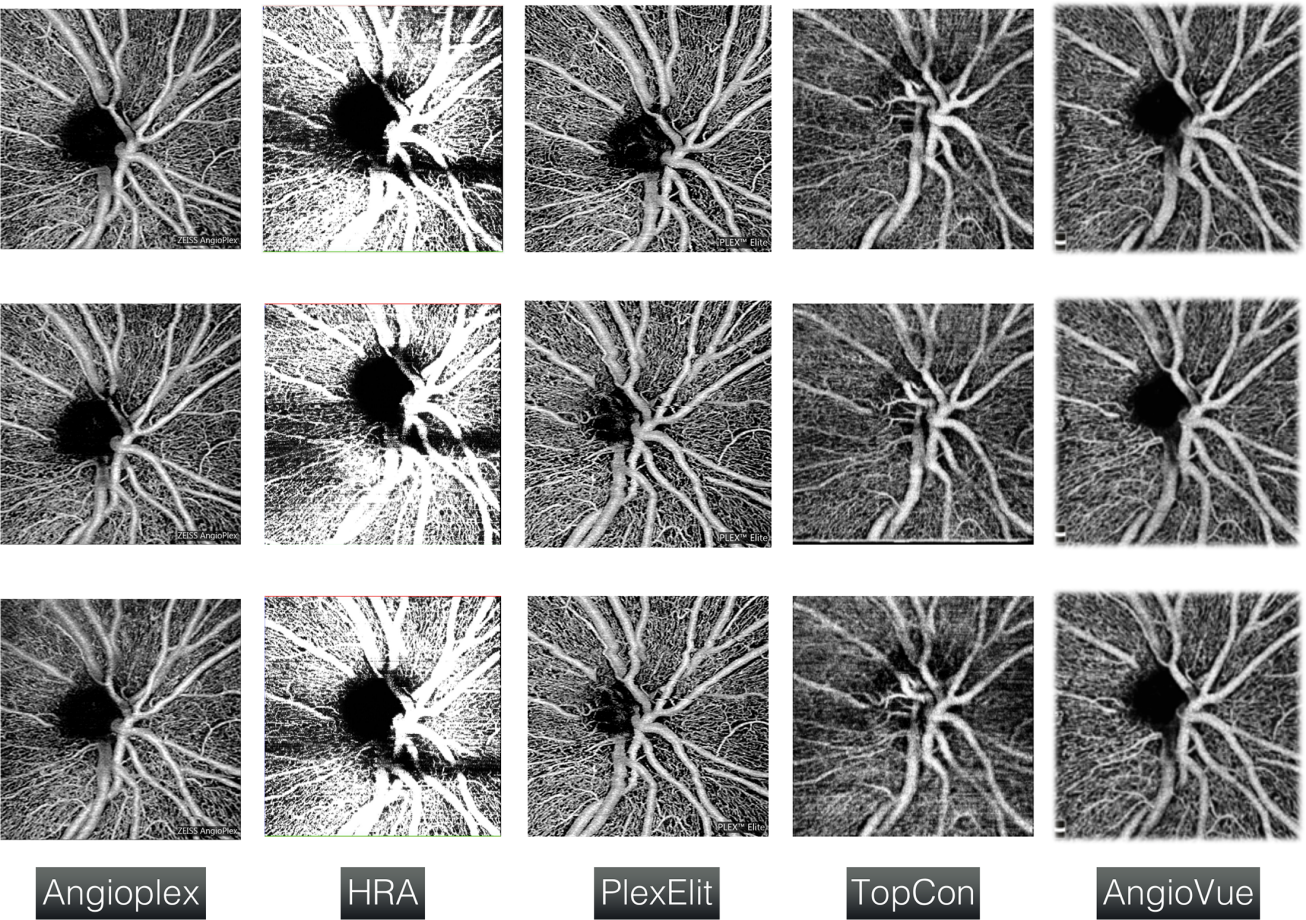
OCT-A scan from different devices (columns), each repeated three times for the same eye (rows).

As visible in Fig 2, images of the same eye recorded with different devices differ significantly from each other. Table 3 summarizes the differences between five devices with respect to the mean gray value of the same eye acquired ten times, and the percentage of fluctuations due to the instrument itself, which ranged between about 1 and 3%. PlexElit had best repeatability, with fluctuations of about 1.08%, then Topcon (1.51%), Angiovue (2.39%), Angioplex (2.92%) and finally the HRA prototype (3.2%). These intrinsic fluctuations mean that a difference between two populations or between two images must be greater than this percentage to be taken into consideration. For our study, based on the Angioplex, the difference between two images therefore had to be greater than 3%.

**Table 3:** Fluctuation due to OCT-A devices.

	Angioplex	HRA	PlexElit	Topcon	Angiovue
Standard deviation	2.92	5.36	1.26	1.49	2.42
Mean gray value	99.82	167.07	115.80	98.06	100.91
Relative fluctuation	2.92	3.2	1.08	1.51	2.39

#### Reproducibility of measurement

**Fig 3:**
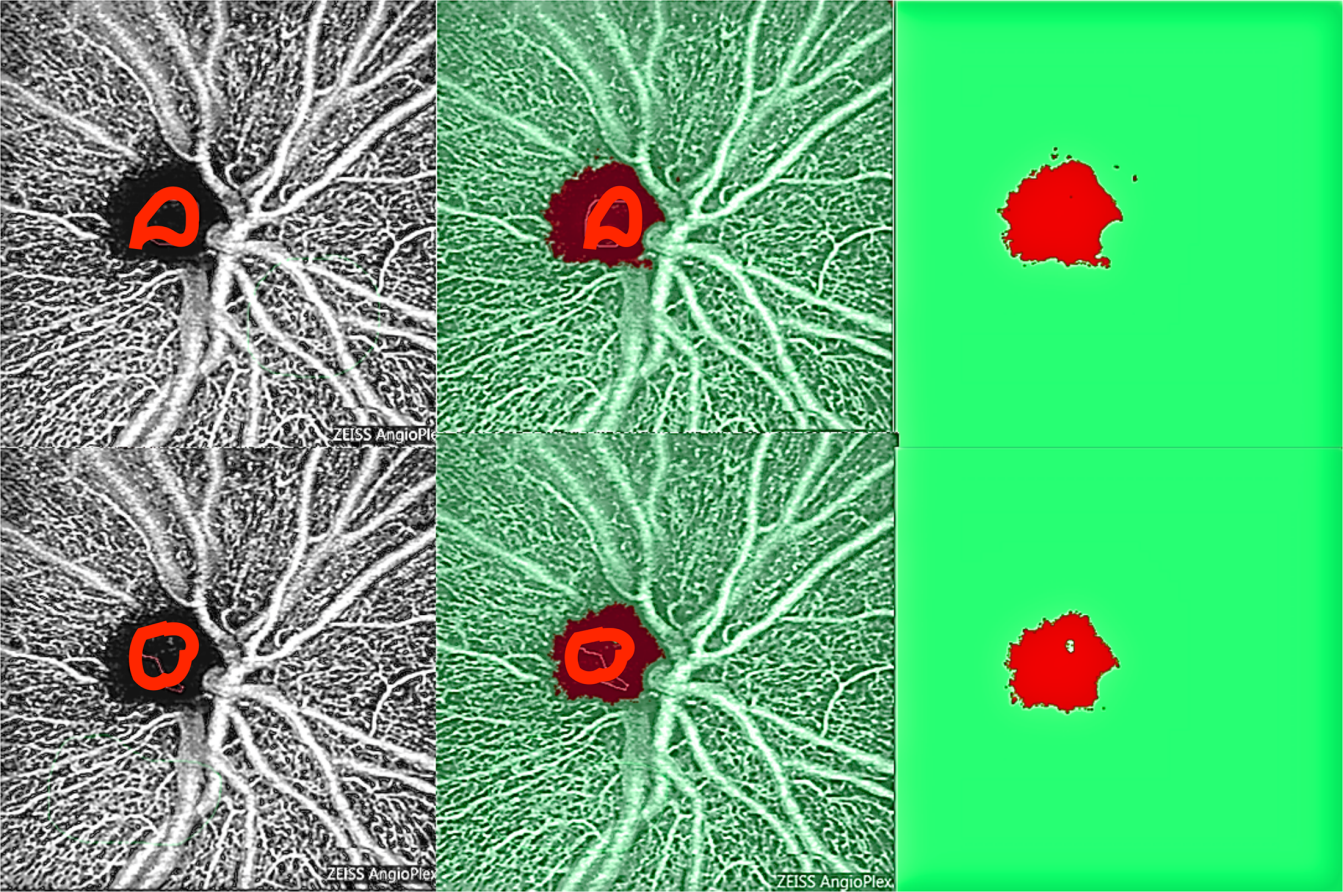
Two different manual annotations for the same eye (top and bottom rows, respectively). The left column superposes the raw images with the corresponding manual annotation (red line) in the avascular area, the right column shows the segmentation result with the avascular region in red and the region with vessels in green, and the middle column is an overlay of the images in the left and right columns.

There is a large number of potential combinations of different manual annotations, as is illustrated in Fig 3 for two different manual annotations of the same eye. For ten annotations tested, the mean standard deviation was 0.31 while the mean gray value was 99.025. These results indicate that fluctuations caused by different manual annotations are on the order of 0.3%, which is negligible compared to the intrinsic fluctuations of each device.

### Application

We compared the peripapillary region with a radius of 640 μm of superficial and deep capillary plexus, with 20 patients for each stage of the disease as shown in Table 4. Given that these samples did not follow a normal distribution, we applied the non-parametric Kruskal-Wallis test to compare averages: The four stages had a statistically different gray level in both the deep (*p* = 10^−5^) and the superficial (*p* = 2.4 ⋅ 10^−13^) vascular networks. A decrease of vascular density around the optic nerve was observed in CPAG as the disease progressed through the four stages. This phenomenon was more visible in the analysis of the superficial capillary network, and was found to be more pronounced with the evolution of the disease. A difference larger than 3% was observed between each stage of development, i.e. larger than the estimated uncertainty due to the device itself (Table 5).

**Table 4:** Mean gray value of peripapillary region of patients suffering from CPAG, stage 1 to 4.

	Stage 1	Stage 2	Stage 3	Stage 4
Superficial capillary network	51.4	37.5	29.4	27.82
Deep capillary network	33.2	25.7	23.7	21.5

**Table 5:** Percentage of difference between each stage for SCN and DCN.

	Stage 1 and 2	Stage 2 and 3	Stage 3 and 4
Superficial capillary network	27	21.6	5.3
Deep capillary network	22.5	7	9

## Discussion

**Fig 4:**
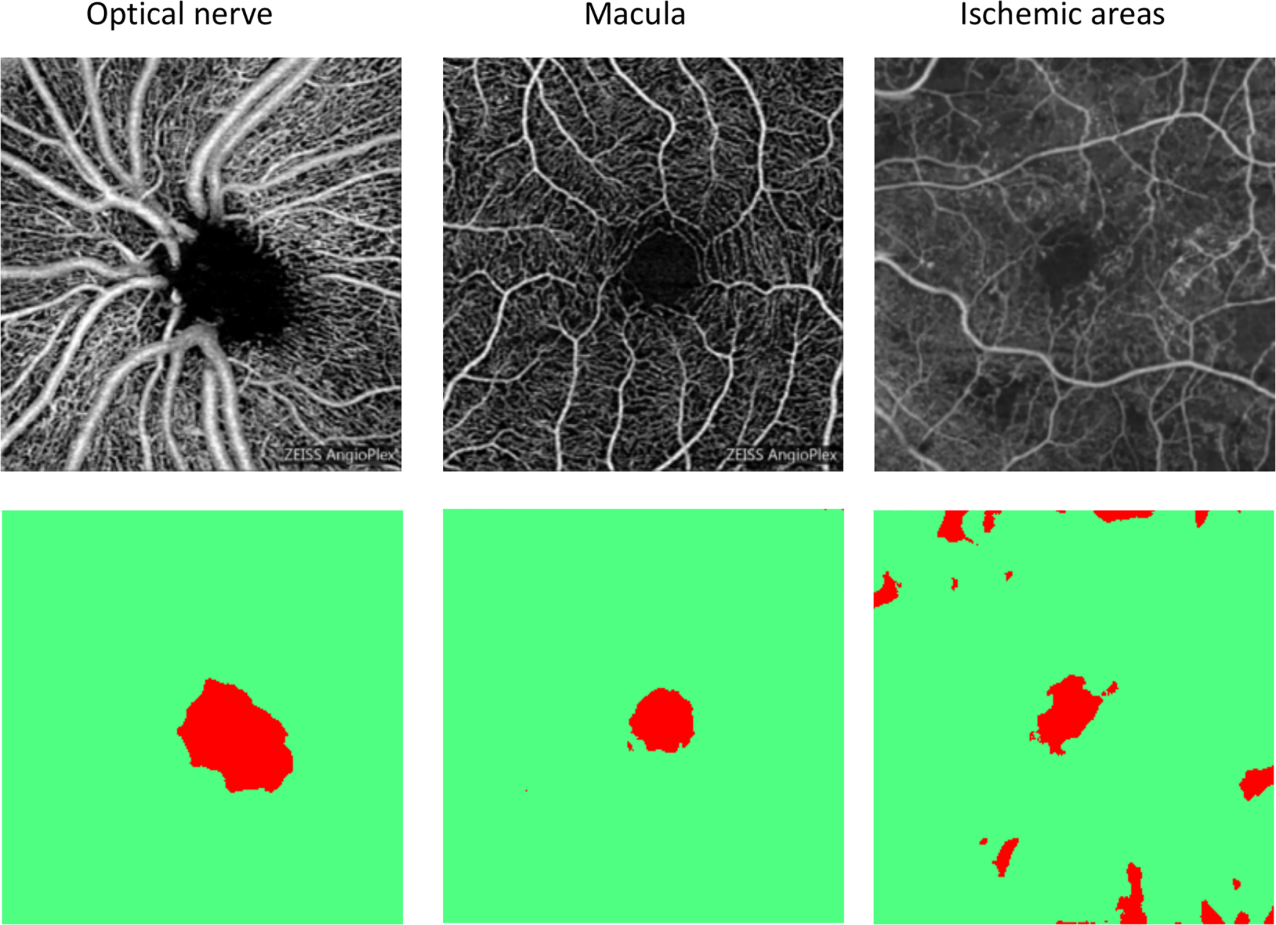
The segmentation procedure of Fig 1 applied to the extraction of various avascular regions (top row). The training of the random forest classifier was carried out with the image of the optic nerve and then applied to the two other images. The bottom row shows the resulting segmentation masks, with red corresponding to the identified avascular regions.

We have demonstrated that the proposed segmentation procedure is suitable for the analysis of the optic nerve and the surrounding region. Moreover, our approach is versatile and in principle applicable to any avascular region, as it adapts automatically to other shapes of the region to be analyzed. Fig 4 illustrates this versatility with the successful application of the random forest classifier, originally trained on an optic-nerve pathology, to other OCT-A images with avascular regions (Macula and ischemic areas). One caveat is that the OCT-A data of Fig 4 were acquired with the same instrument; as the contrast can differ from one OCT-A device to another, see Fig 2, it might be necessary to train such a classifier for each device separately.

The various devices of our comparison give us an indication of the apparent fluctuations of the vascular density generated by the instruments themselves, which were found to be of the order of 1 to 3%. This type of repeatability analysis was introduced in OCT angiography only very recently [26, 29, 30]. The work of Munk et al. [26] compared qualitatively 4 OCT devices for angiography with respect to the presence or absence of artifacts, the continuity of the vessels, the clarity of the central avascular zone and the discriminating power of small vessels relative to the larger ones. These authors furthermore evaluated the instrumental performance quantitatively by calculating the vascular density of the surface network, for which they did not find any significant differences between the four tested devices. Based on the qualitative criteria, the Zeiss AngioPlex seemed the most reliable, followed by Optovue, Topcon and finally Heidelberg. Our results on repeatability found PlexElit in first position, followed by Topcon, Optovue, Angioplex, and Heidelberg. Men et al. [30] studied the repeatability of the determination of vascular density in the skin. Based on measurements of four different areas, each of which was repeated three times, they found a coefficient of variation ranging from 2 to 2.7 for all locations, meaning that the differences were not statistically significant.

Other related works include Refs. [31] and [32]. Al Sheikh et al. [31] compared vascular density and central avascular area of 24 healthy eyes using swept-source and spectral domain OCT angiography, two OCT methods differing mainly in the wavelength used. Theses authors found a non-significant difference between the two devices for the superficial and deep vascular networks.

Guo et al [32] evaluated the repeatability of the AngioPlex measurement of the central avascular area by performing an OCT angiogram in 25 healthy controls on 3 occasions. The central avascular zone was measured with ImageJ as used by two different observers. The difference noted between the two observers was minimal and the authors concluded that the area of the central avascular area could be determined with good repeatability. Our results, based on an automated segmentation of the region of interest, indicated a threshold above which a difference can be attributed to a disease of 1 − 3%, depending on the device. This quantification of the instrumentational uncertainty is important for any tasks of interest in ophtalmology. Furthermore, we have demonstrated the feasibility of precise segmentation of specific regions with the random forest algorithm, suggesting a wide applicability of their approach. For instance, Sakaguchi and al. [25] have recently demonstrated that peripapillary RNFL vessel density and thickness had different characteristic sectoral structure-function relationships in glaucoma. An RNFL vessel density measurement must take into account the presence of peripapillary atrophy, hence the importance of precise segmentation of the region of interest such as the one provided by the approach introduced in this article.

The present study offers several perspectives for further improvement. First, we did not have access to the raw image data and were limited to the image formats made available to the end user. Higher dynamic range (number of bits) and improved contrast could be obtained by applying the global methodology presented here to raw device data. Second, our method of performing OCT angiography of the optic nerve required manual positioning of the light source, meaning that the quality of an image can depend on the operator. Third, one prototype was tested against commercially available modules, therefore it remains to be seen how the performance of the final version of the PlexElit prototype will compare to the other available modules. Finally, the position of the segmentation lines for the deep regions was obtained automatically from a fixed offset to the upper surface of the retina. While this fixed-offset strategy is convenient because it corresponds to an objective criterion, it does not account for a potential variability of the retina shape among the population of patients. In this study, we systematically checked the success of this automated procedure for each image by visual inspection of the OCT, which was possible because we were working on small cohorts. It would therefore be important to implement an automated extraction of anatomic layers in the retina from the vascular content instead of working at a fixed distance to the surface.

## Conclusion

This work allowed us to develop two distinct points: the automated segmentation of the avascular region of interest in OCT-A images without prior knowledge of its shape and the study of the repeatability of the segmentation technique. We realized this study at two different depths of analysis.

There are other ways of quantifying vascular structure, such as counting vessels, measuring diameter, length, bifurcations. However, the segmentation of the area of interest is an indispensable first step independent of the type of quantitative analysis performed subsequently. Integrating modules for automated segmentation in the devices would allow immediate access to these regions of interest.

An essential parameter for OCT-A based work is the repeatability of image acquisitions. Here, we have found fluctuations of the apparent vascular density ranging from 1 to 3% depending on the specific OCT-A device. We believe this information should be transmitted to clinicians to help them to be fully aware of the limitations of the technique. It could for instance be taken into account in accuracy studies on Glaucoma in addition to the current procedure which rather stress the possible bias due to the visual rating of experts [33].

## Supporting information

S1 File Images used for study. (ZIP)

S2 File Macro Dilat 100 et 60. (ZIP)
