# Supplementary figures and images for: Toward quantitative and reproducible clinical use of OCT-Angiography (part 2 of 2)
Source: PLoS One. 2018 Jul 6;13(7):e0197588. doi: 10.1371/journal.pone.0197588 (PMC6034792; doi:10.1371/journal.pone.0197588)

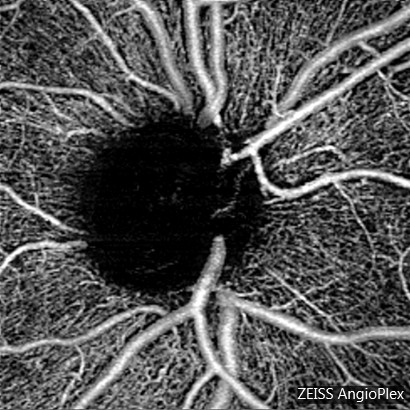

Supplement: S1 File — (ZIP) [file pone.0197588.s001.zip › Data Article Plos/Section application/Superficial network/Stade 1/14.jpg]

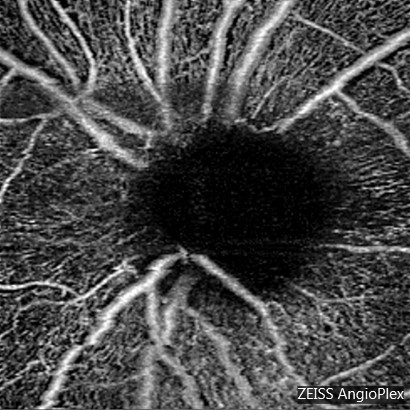

Supplement: S1 File — (ZIP) [file pone.0197588.s001.zip › Data Article Plos/Section application/Superficial network/Stade 1/15.jpg]

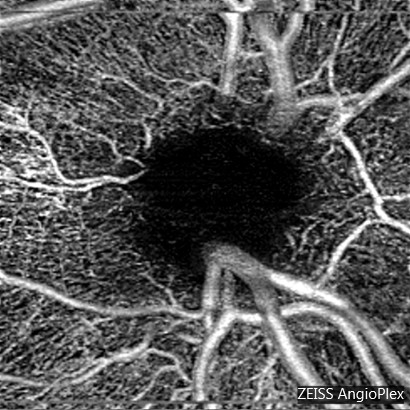

Supplement: S1 File — (ZIP) [file pone.0197588.s001.zip › Data Article Plos/Section application/Superficial network/Stade 1/16.jpg]

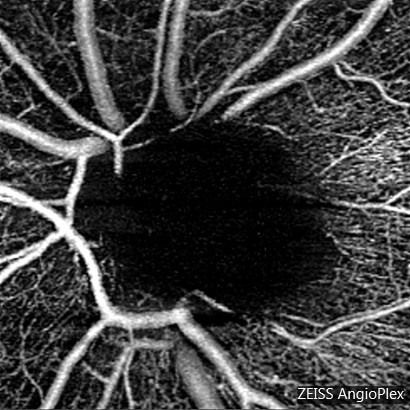

Supplement: S1 File — (ZIP) [file pone.0197588.s001.zip › Data Article Plos/Section application/Superficial network/Stade 1/18.jpg]

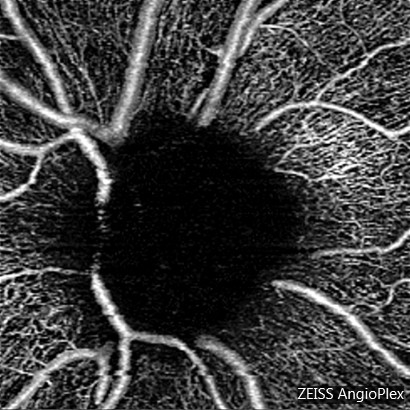

Supplement: S1 File — (ZIP) [file pone.0197588.s001.zip › Data Article Plos/Section application/Superficial network/Stade 1/2.jpg]

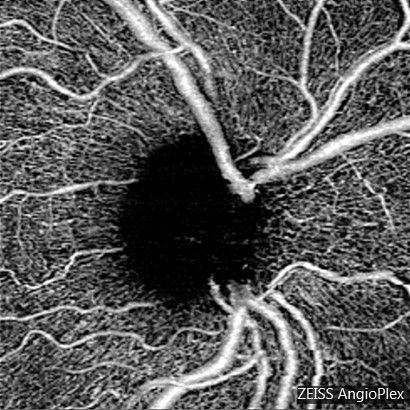

Supplement: S1 File — (ZIP) [file pone.0197588.s001.zip › Data Article Plos/Section application/Superficial network/Stade 1/20.jpg]

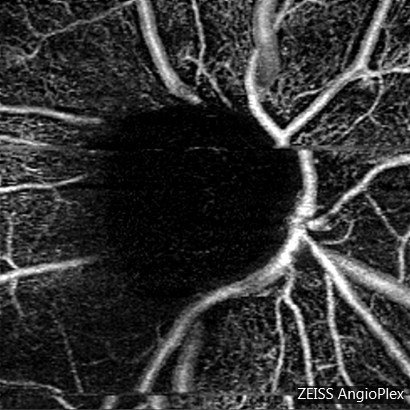

Supplement: S1 File — (ZIP) [file pone.0197588.s001.zip › Data Article Plos/Section application/Superficial network/Stade 1/3.jpg]

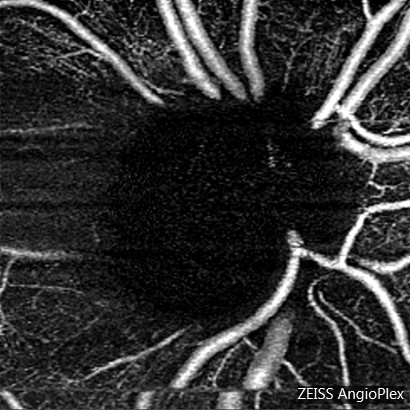

Supplement: S1 File — (ZIP) [file pone.0197588.s001.zip › Data Article Plos/Section application/Superficial network/Stade 1/4.jpg]

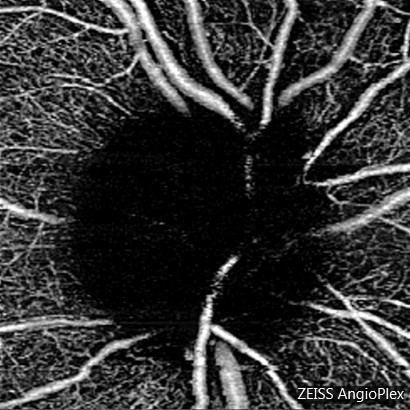

Supplement: S1 File — (ZIP) [file pone.0197588.s001.zip › Data Article Plos/Section application/Superficial network/Stade 1/5.jpg]

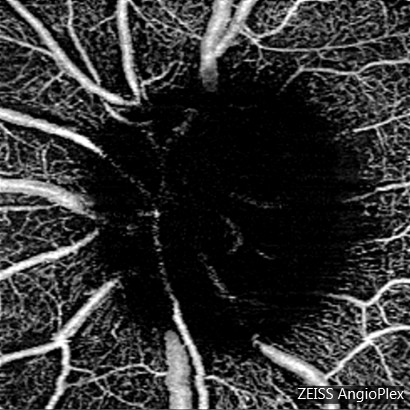

Supplement: S1 File — (ZIP) [file pone.0197588.s001.zip › Data Article Plos/Section application/Superficial network/Stade 1/6.jpg]

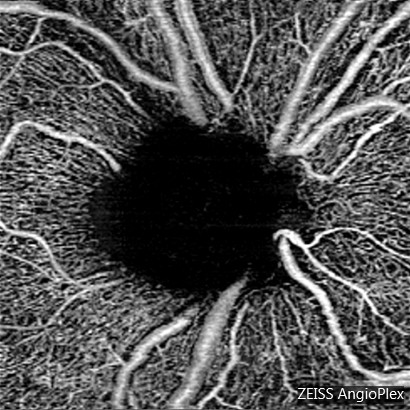

Supplement: S1 File — (ZIP) [file pone.0197588.s001.zip › Data Article Plos/Section application/Superficial network/Stade 1/7.jpg]

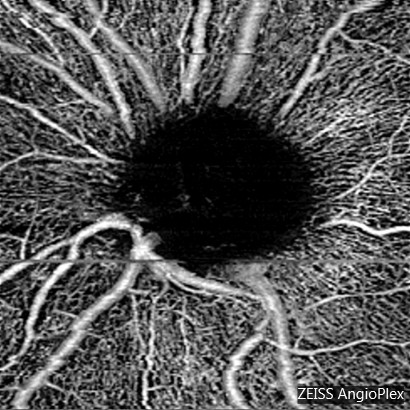

Supplement: S1 File — (ZIP) [file pone.0197588.s001.zip › Data Article Plos/Section application/Superficial network/Stade 1/8.jpg]

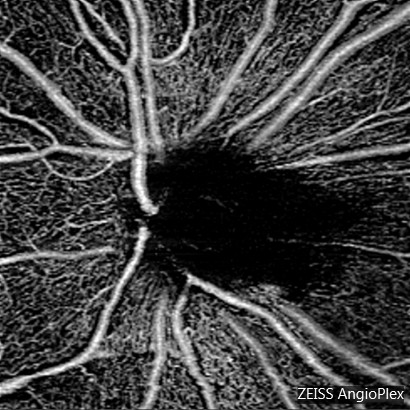

Supplement: S1 File — (ZIP) [file pone.0197588.s001.zip › Data Article Plos/Section application/Superficial network/Stade 1/9.jpg]

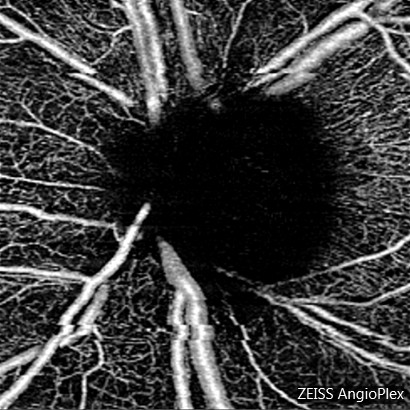

Supplement: S1 File — (ZIP) [file pone.0197588.s001.zip › Data Article Plos/Section application/Superficial network/Stade 2/1.jpg]

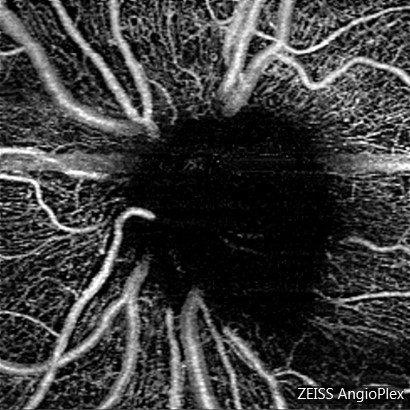

Supplement: S1 File — (ZIP) [file pone.0197588.s001.zip › Data Article Plos/Section application/Superficial network/Stade 2/10.jpg]

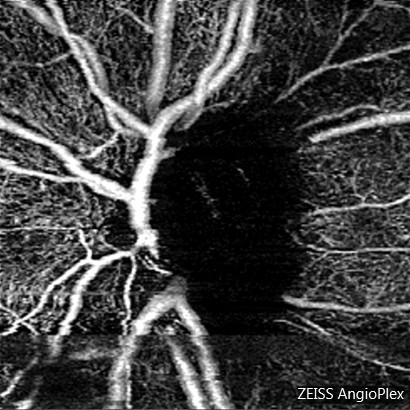

Supplement: S1 File — (ZIP) [file pone.0197588.s001.zip › Data Article Plos/Section application/Superficial network/Stade 2/11.jpg]

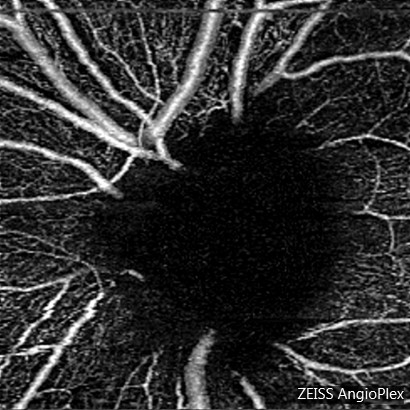

Supplement: S1 File — (ZIP) [file pone.0197588.s001.zip › Data Article Plos/Section application/Superficial network/Stade 2/12.jpg]

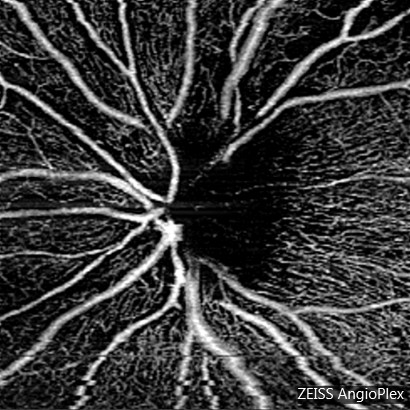

Supplement: S1 File — (ZIP) [file pone.0197588.s001.zip › Data Article Plos/Section application/Superficial network/Stade 2/13.jpg]

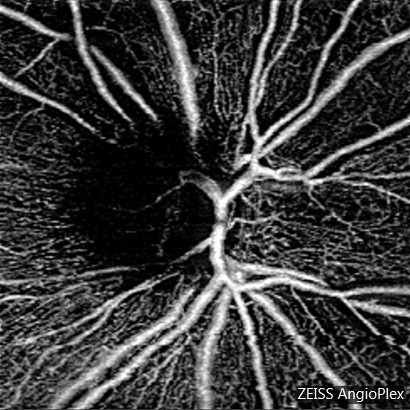

Supplement: S1 File — (ZIP) [file pone.0197588.s001.zip › Data Article Plos/Section application/Superficial network/Stade 2/14.jpg]

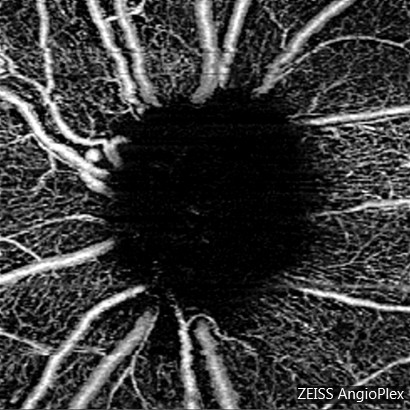

Supplement: S1 File — (ZIP) [file pone.0197588.s001.zip › Data Article Plos/Section application/Superficial network/Stade 2/15.jpg]

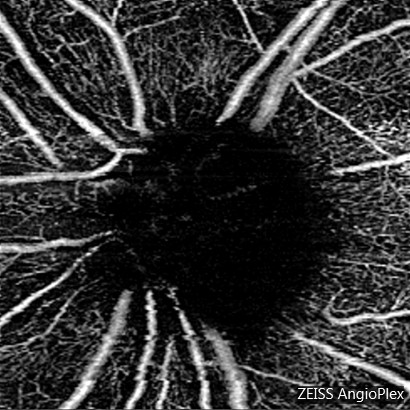

Supplement: S1 File — (ZIP) [file pone.0197588.s001.zip › Data Article Plos/Section application/Superficial network/Stade 2/16.jpg]

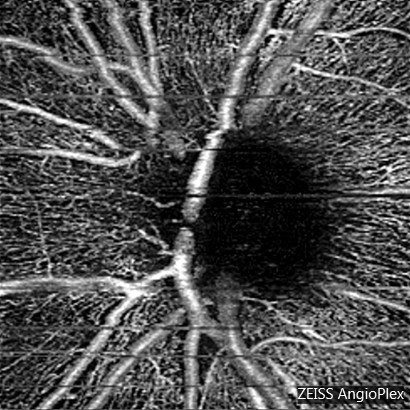

Supplement: S1 File — (ZIP) [file pone.0197588.s001.zip › Data Article Plos/Section application/Superficial network/Stade 2/17.jpg]

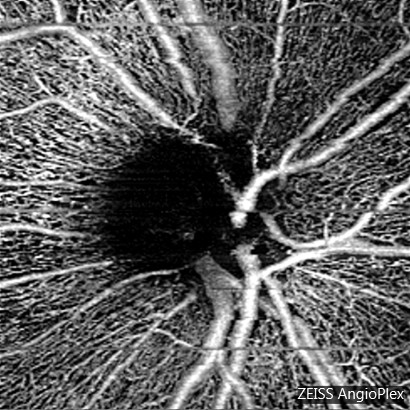

Supplement: S1 File — (ZIP) [file pone.0197588.s001.zip › Data Article Plos/Section application/Superficial network/Stade 2/18.jpg]

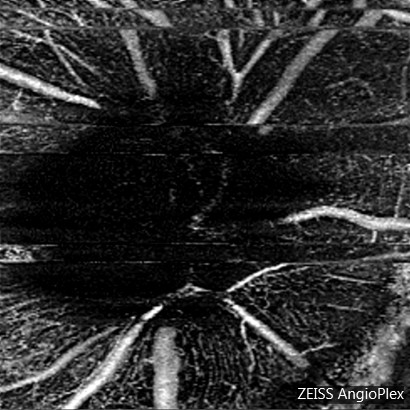

Supplement: S1 File — (ZIP) [file pone.0197588.s001.zip › Data Article Plos/Section application/Superficial network/Stade 2/19.jpg]

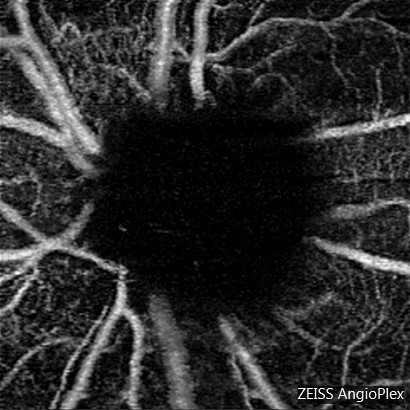

Supplement: S1 File — (ZIP) [file pone.0197588.s001.zip › Data Article Plos/Section application/Superficial network/Stade 2/2.jpg]

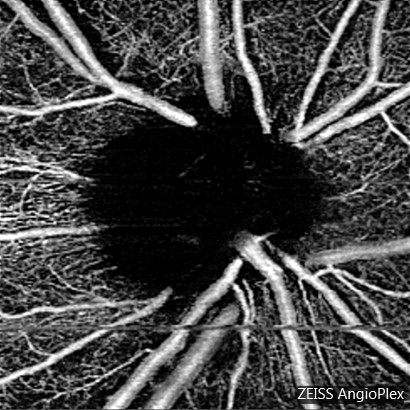

Supplement: S1 File — (ZIP) [file pone.0197588.s001.zip › Data Article Plos/Section application/Superficial network/Stade 2/20.jpg]

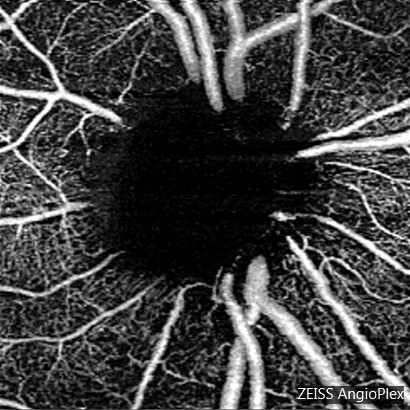

Supplement: S1 File — (ZIP) [file pone.0197588.s001.zip › Data Article Plos/Section application/Superficial network/Stade 2/3.jpg]

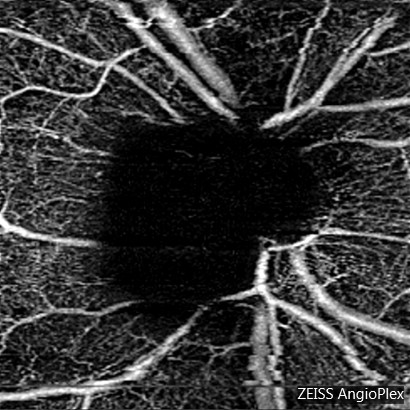

Supplement: S1 File — (ZIP) [file pone.0197588.s001.zip › Data Article Plos/Section application/Superficial network/Stade 2/4.jpg]

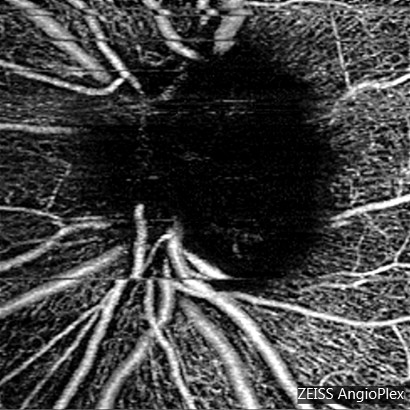

Supplement: S1 File — (ZIP) [file pone.0197588.s001.zip › Data Article Plos/Section application/Superficial network/Stade 2/5.jpg]

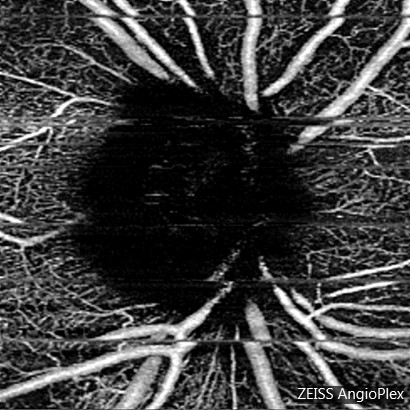

Supplement: S1 File — (ZIP) [file pone.0197588.s001.zip › Data Article Plos/Section application/Superficial network/Stade 2/6.jpg]

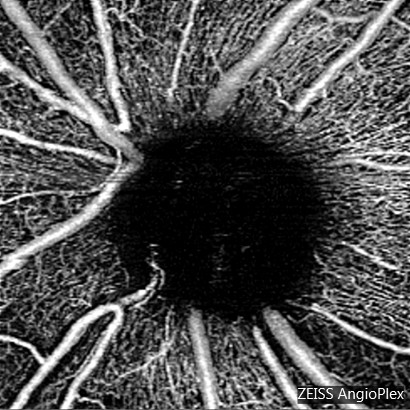

Supplement: S1 File — (ZIP) [file pone.0197588.s001.zip › Data Article Plos/Section application/Superficial network/Stade 2/7.jpg]

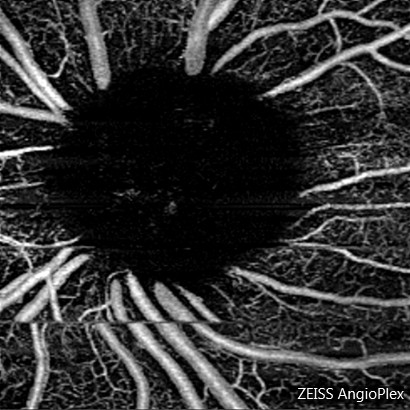

Supplement: S1 File — (ZIP) [file pone.0197588.s001.zip › Data Article Plos/Section application/Superficial network/Stade 2/8.jpg]

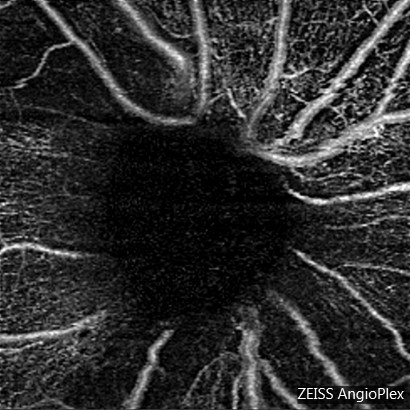

Supplement: S1 File — (ZIP) [file pone.0197588.s001.zip › Data Article Plos/Section application/Superficial network/Stade 2/9.jpg]

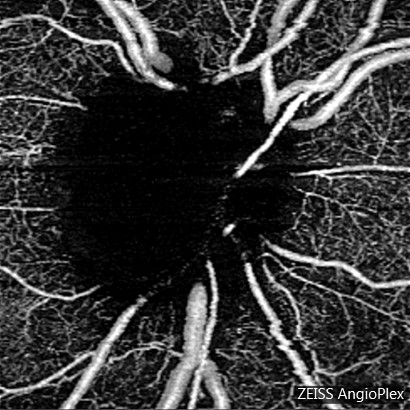

Supplement: S1 File — (ZIP) [file pone.0197588.s001.zip › Data Article Plos/Section application/Superficial network/Stade 4/1.jpg]

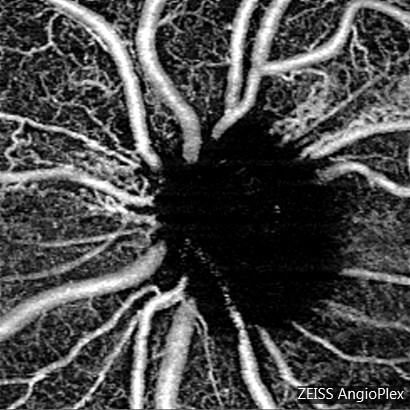

Supplement: S1 File — (ZIP) [file pone.0197588.s001.zip › Data Article Plos/Section application/Superficial network/Stade 4/10.jpg]

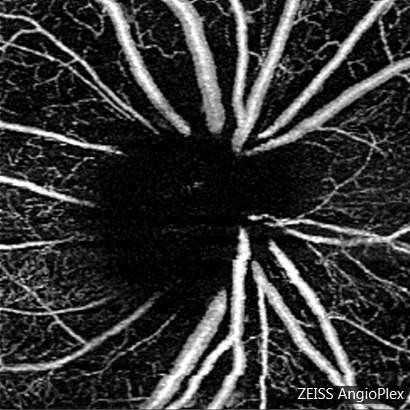

Supplement: S1 File — (ZIP) [file pone.0197588.s001.zip › Data Article Plos/Section application/Superficial network/Stade 4/11.jpg]

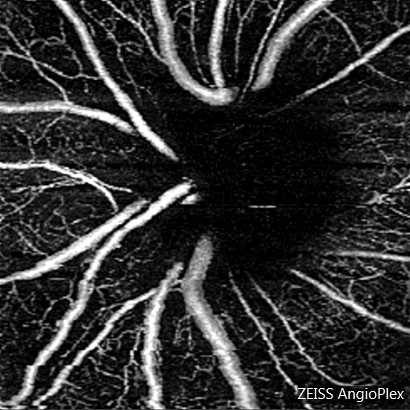

Supplement: S1 File — (ZIP) [file pone.0197588.s001.zip › Data Article Plos/Section application/Superficial network/Stade 4/12.jpg]

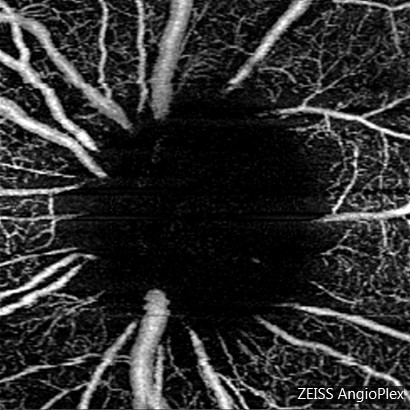

Supplement: S1 File — (ZIP) [file pone.0197588.s001.zip › Data Article Plos/Section application/Superficial network/Stade 4/13.jpg]

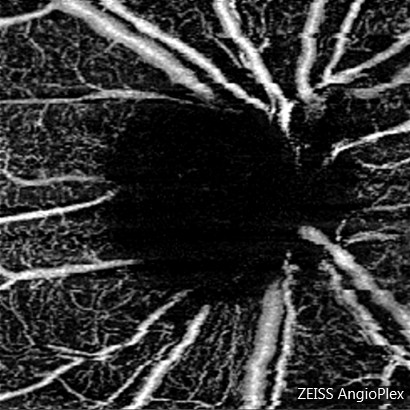

Supplement: S1 File — (ZIP) [file pone.0197588.s001.zip › Data Article Plos/Section application/Superficial network/Stade 4/14.jpg]

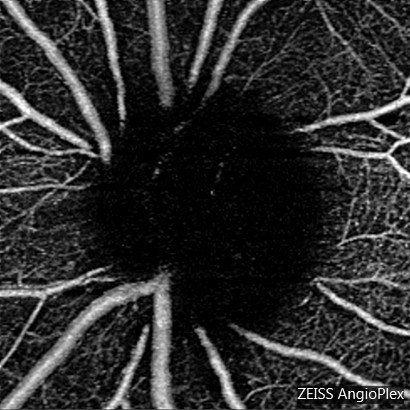

Supplement: S1 File — (ZIP) [file pone.0197588.s001.zip › Data Article Plos/Section application/Superficial network/Stade 4/15.jpg]

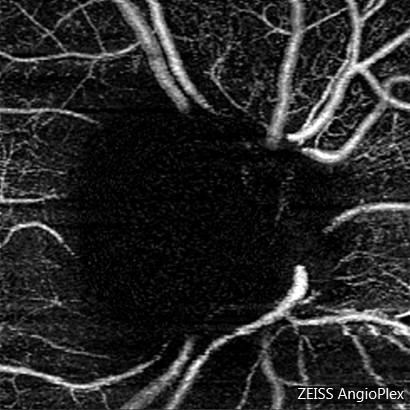

Supplement: S1 File — (ZIP) [file pone.0197588.s001.zip › Data Article Plos/Section application/Superficial network/Stade 4/16.jpg]

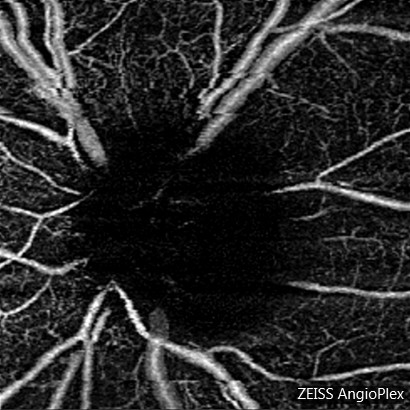

Supplement: S1 File — (ZIP) [file pone.0197588.s001.zip › Data Article Plos/Section application/Superficial network/Stade 4/18.jpg]

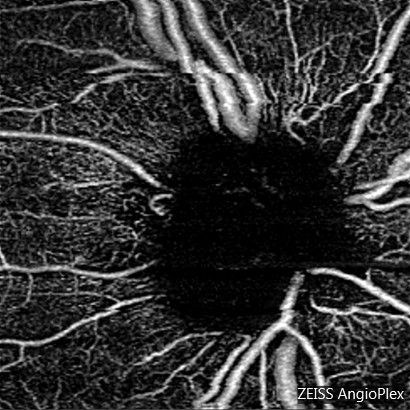

Supplement: S1 File — (ZIP) [file pone.0197588.s001.zip › Data Article Plos/Section application/Superficial network/Stade 4/19.jpg]

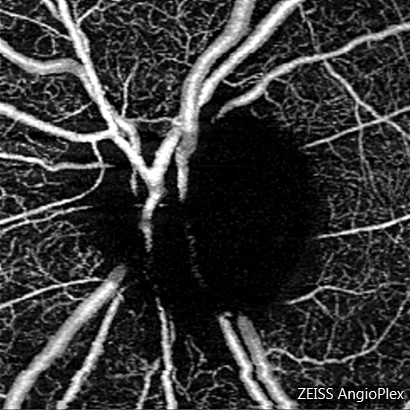

Supplement: S1 File — (ZIP) [file pone.0197588.s001.zip › Data Article Plos/Section application/Superficial network/Stade 4/2.jpg]

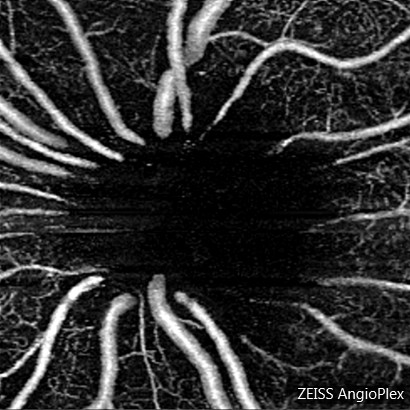

Supplement: S1 File — (ZIP) [file pone.0197588.s001.zip › Data Article Plos/Section application/Superficial network/Stade 4/3.jpg]

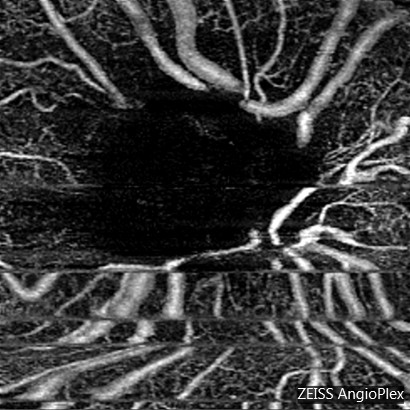

Supplement: S1 File — (ZIP) [file pone.0197588.s001.zip › Data Article Plos/Section application/Superficial network/Stade 4/4.jpg]

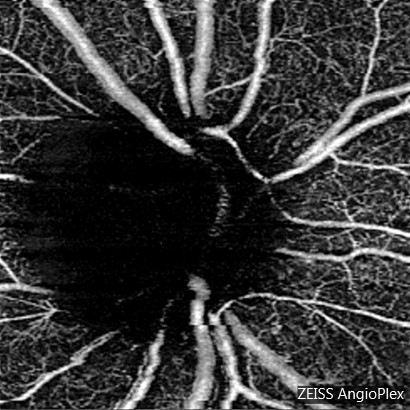

Supplement: S1 File — (ZIP) [file pone.0197588.s001.zip › Data Article Plos/Section application/Superficial network/Stade 4/5.jpg]

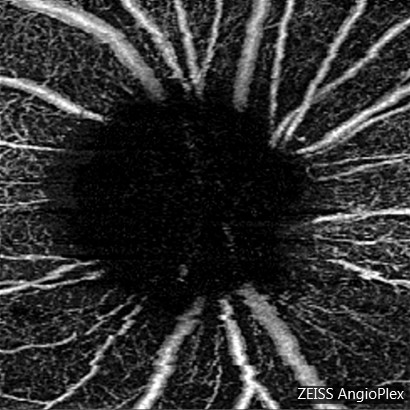

Supplement: S1 File — (ZIP) [file pone.0197588.s001.zip › Data Article Plos/Section application/Superficial network/Stade 4/6.jpg]

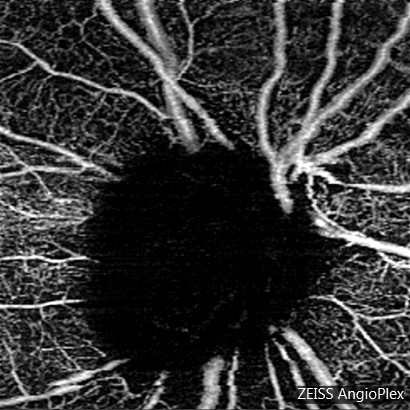

Supplement: S1 File — (ZIP) [file pone.0197588.s001.zip › Data Article Plos/Section application/Superficial network/Stade 4/7.jpg]

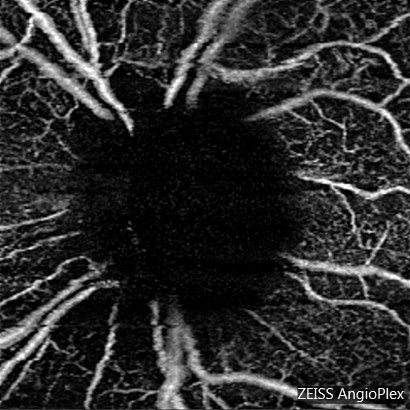

Supplement: S1 File — (ZIP) [file pone.0197588.s001.zip › Data Article Plos/Section application/Superficial network/Stade 4/8.jpg]

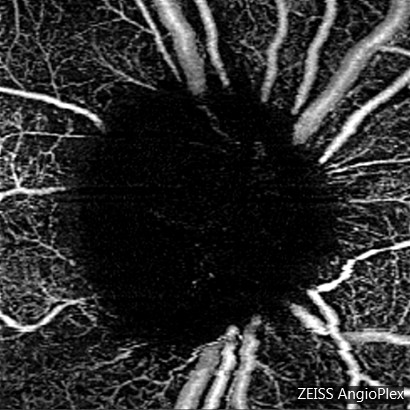

Supplement: S1 File — (ZIP) [file pone.0197588.s001.zip › Data Article Plos/Section application/Superficial network/Stade 4/9.jpg]

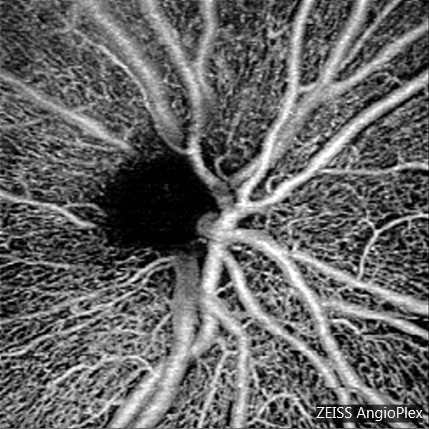

Supplement: S1 File — (ZIP) [file pone.0197588.s001.zip › Data Article Plos/Section repeatability/OCT A 5 appareils/Angioplex/1.jpg]

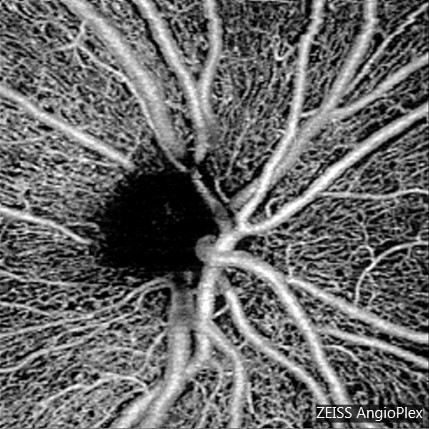

Supplement: S1 File — (ZIP) [file pone.0197588.s001.zip › Data Article Plos/Section repeatability/OCT A 5 appareils/Angioplex/2.jpg]

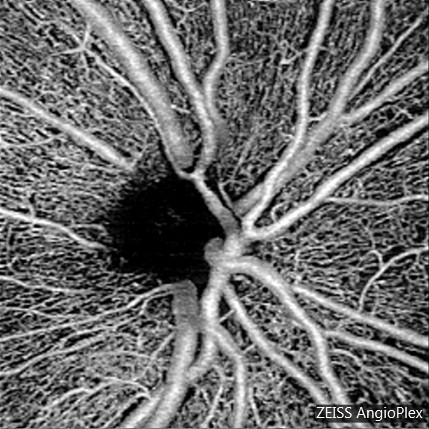

Supplement: S1 File — (ZIP) [file pone.0197588.s001.zip › Data Article Plos/Section repeatability/OCT A 5 appareils/Angioplex/3.jpg]

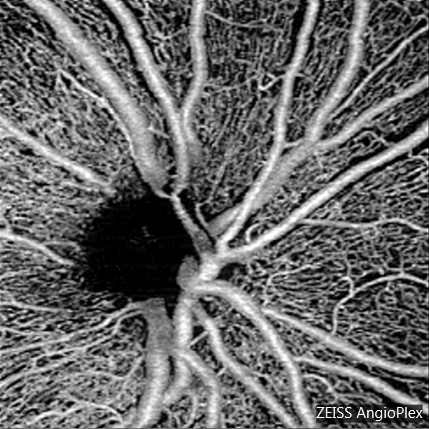

Supplement: S1 File — (ZIP) [file pone.0197588.s001.zip › Data Article Plos/Section repeatability/OCT A 5 appareils/Angioplex/4.jpg]

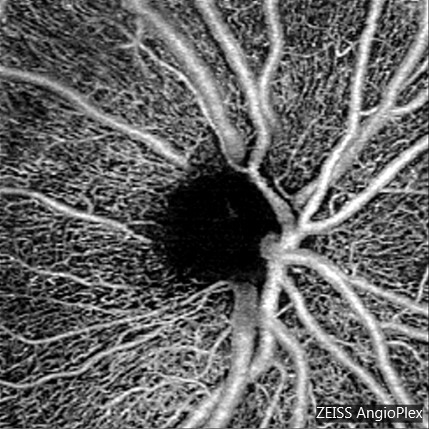

Supplement: S1 File — (ZIP) [file pone.0197588.s001.zip › Data Article Plos/Section repeatability/OCT A 5 appareils/Angioplex/5.jpg]

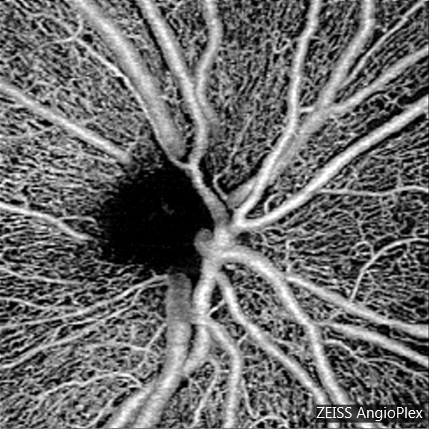

Supplement: S1 File — (ZIP) [file pone.0197588.s001.zip › Data Article Plos/Section repeatability/OCT A 5 appareils/Angioplex/6.jpg]

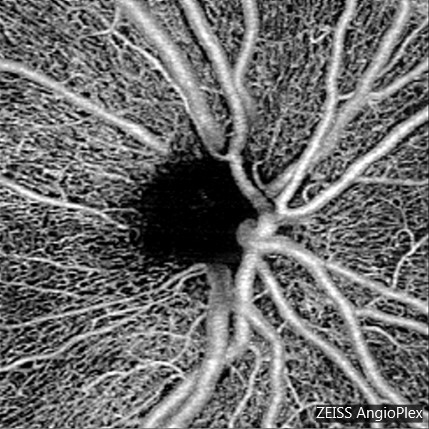

Supplement: S1 File — (ZIP) [file pone.0197588.s001.zip › Data Article Plos/Section repeatability/OCT A 5 appareils/Angioplex/7.jpg]

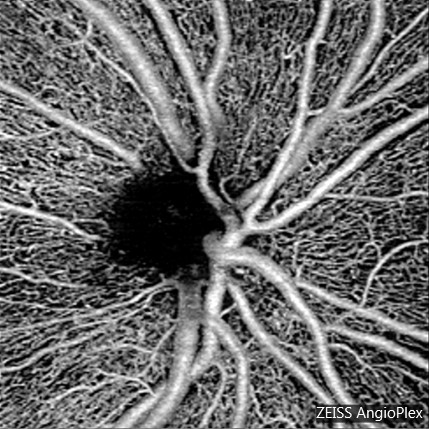

Supplement: S1 File — (ZIP) [file pone.0197588.s001.zip › Data Article Plos/Section repeatability/OCT A 5 appareils/Angioplex/8.jpg]

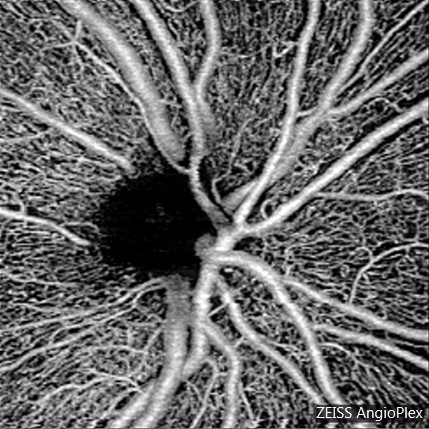

Supplement: S1 File — (ZIP) [file pone.0197588.s001.zip › Data Article Plos/Section repeatability/OCT A 5 appareils/Angioplex/9.jpg]

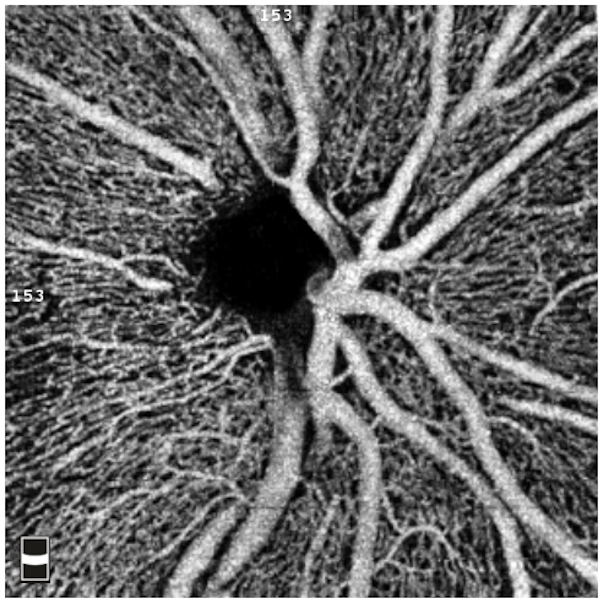

Supplement: S1 File — (ZIP) [file pone.0197588.s001.zip › Data Article Plos/Section repeatability/OCT A 5 appareils/ANGIOVUE/1.png]

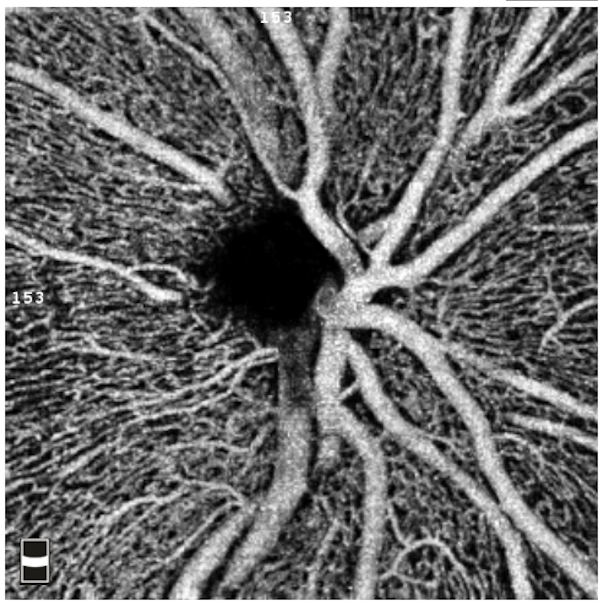

Supplement: S1 File — (ZIP) [file pone.0197588.s001.zip › Data Article Plos/Section repeatability/OCT A 5 appareils/ANGIOVUE/10.png]

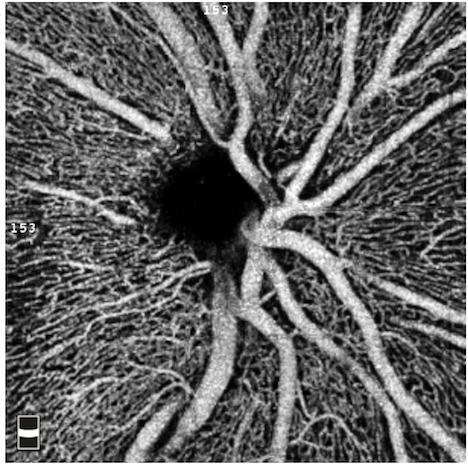

Supplement: S1 File — (ZIP) [file pone.0197588.s001.zip › Data Article Plos/Section repeatability/OCT A 5 appareils/ANGIOVUE/2.png]

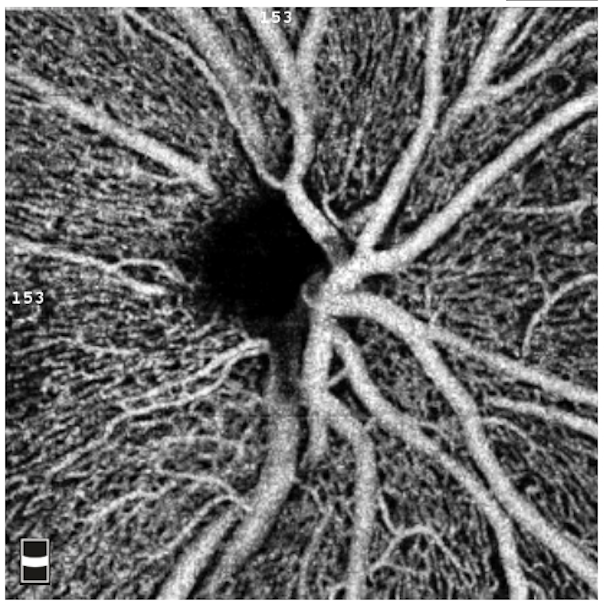

Supplement: S1 File — (ZIP) [file pone.0197588.s001.zip › Data Article Plos/Section repeatability/OCT A 5 appareils/ANGIOVUE/3.png]

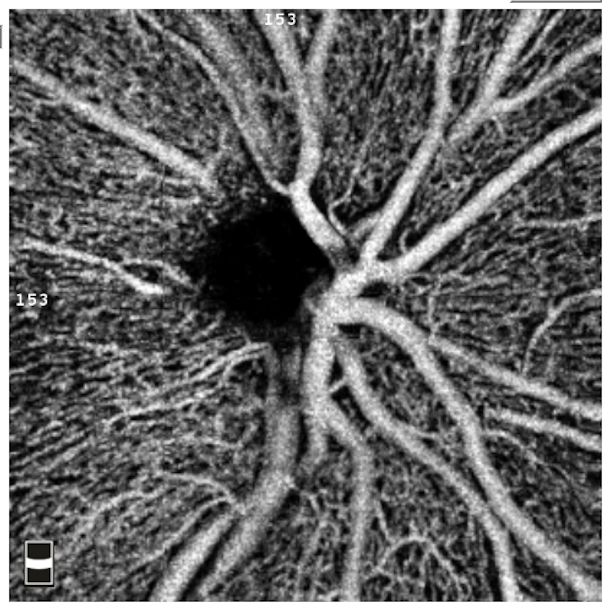

Supplement: S1 File — (ZIP) [file pone.0197588.s001.zip › Data Article Plos/Section repeatability/OCT A 5 appareils/ANGIOVUE/4.png]

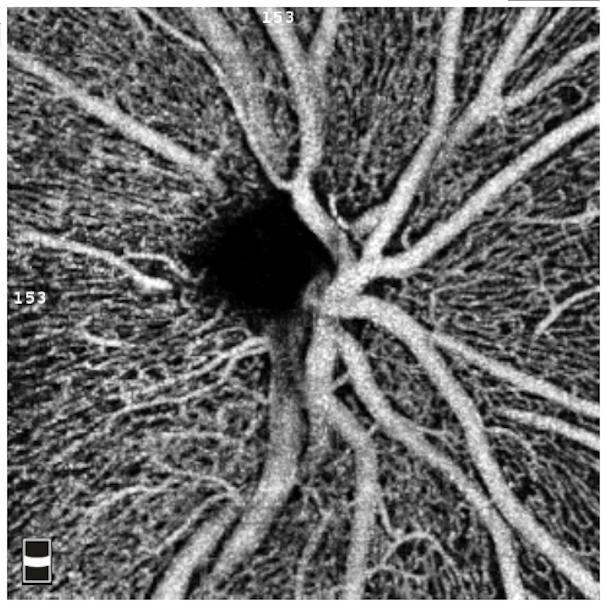

Supplement: S1 File — (ZIP) [file pone.0197588.s001.zip › Data Article Plos/Section repeatability/OCT A 5 appareils/ANGIOVUE/5.png]

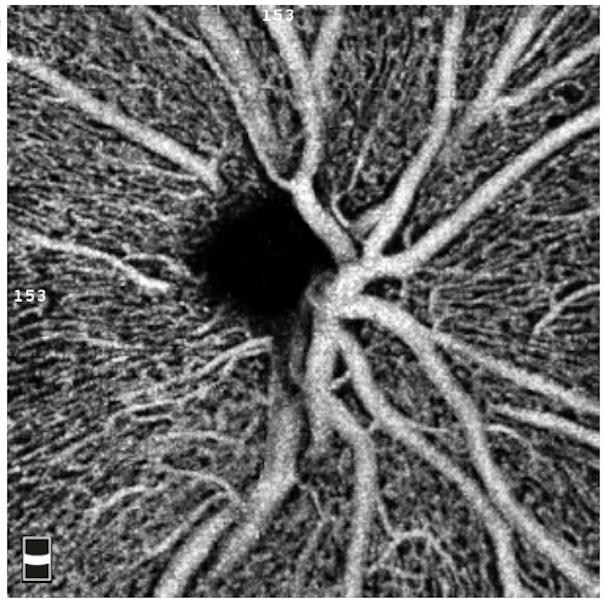

Supplement: S1 File — (ZIP) [file pone.0197588.s001.zip › Data Article Plos/Section repeatability/OCT A 5 appareils/ANGIOVUE/6.png]

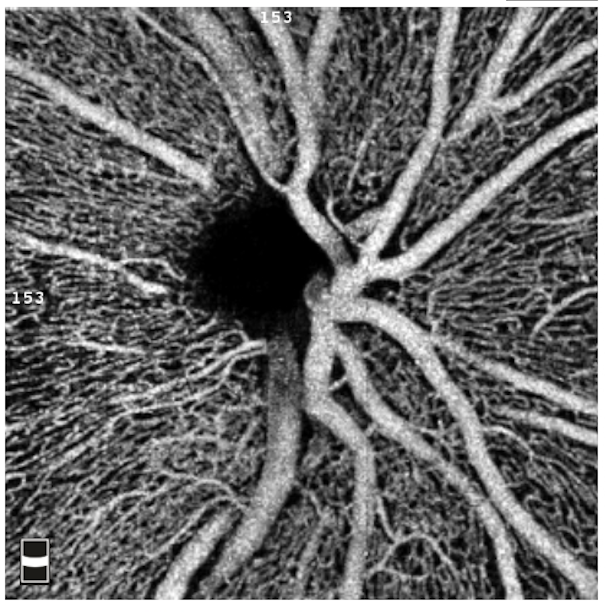

Supplement: S1 File — (ZIP) [file pone.0197588.s001.zip › Data Article Plos/Section repeatability/OCT A 5 appareils/ANGIOVUE/7.png]

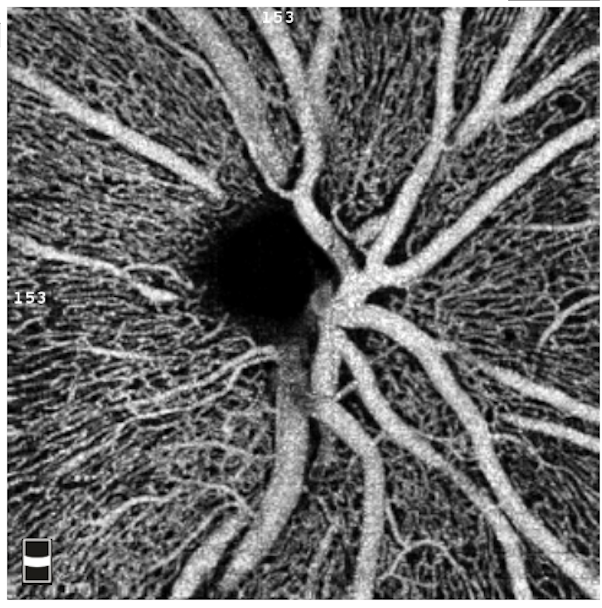

Supplement: S1 File — (ZIP) [file pone.0197588.s001.zip › Data Article Plos/Section repeatability/OCT A 5 appareils/ANGIOVUE/8.png]

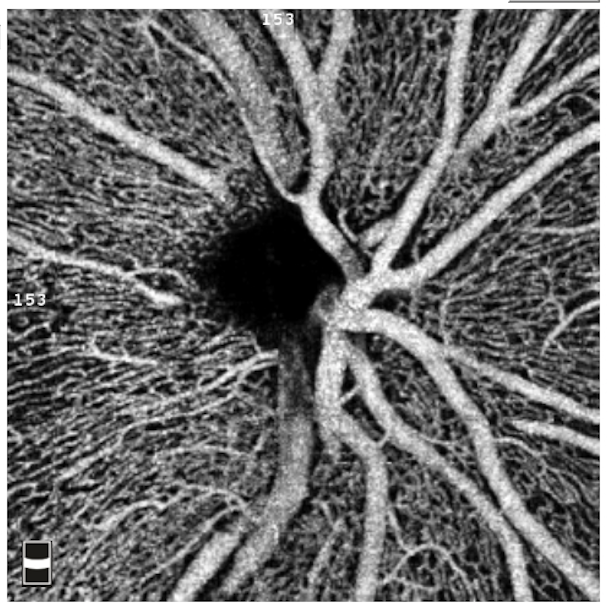

Supplement: S1 File — (ZIP) [file pone.0197588.s001.zip › Data Article Plos/Section repeatability/OCT A 5 appareils/ANGIOVUE/9.png]

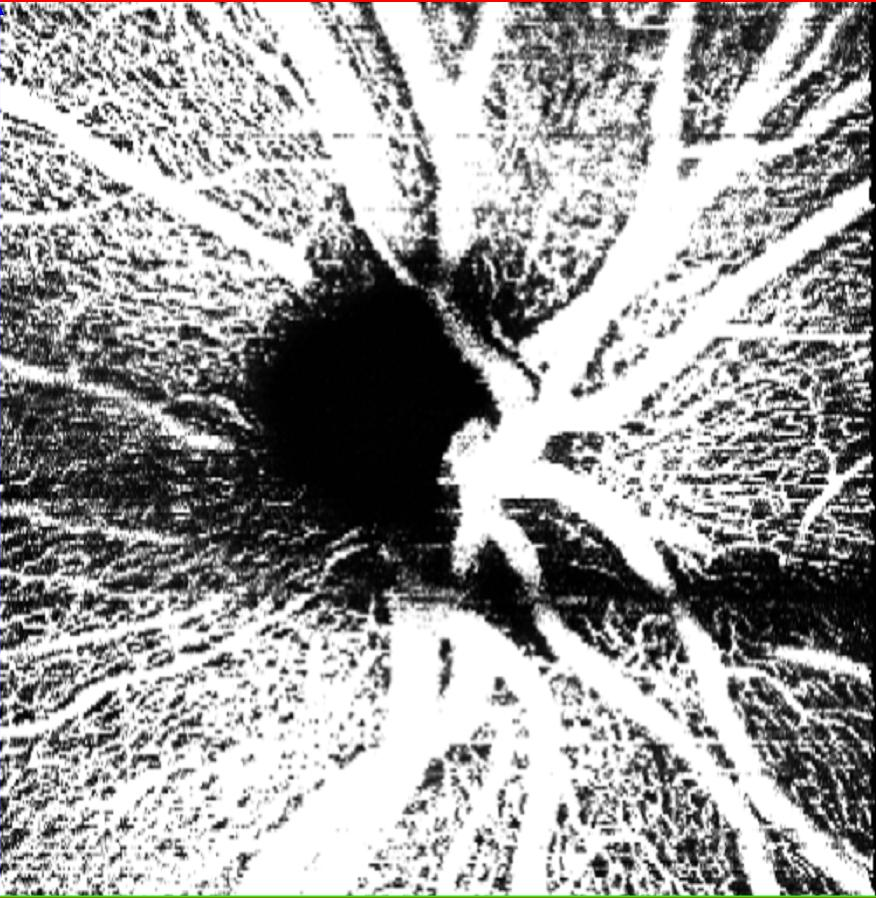

Supplement: S1 File — (ZIP) [file pone.0197588.s001.zip › Data Article Plos/Section repeatability/OCT A 5 appareils/HRA/1.tif]

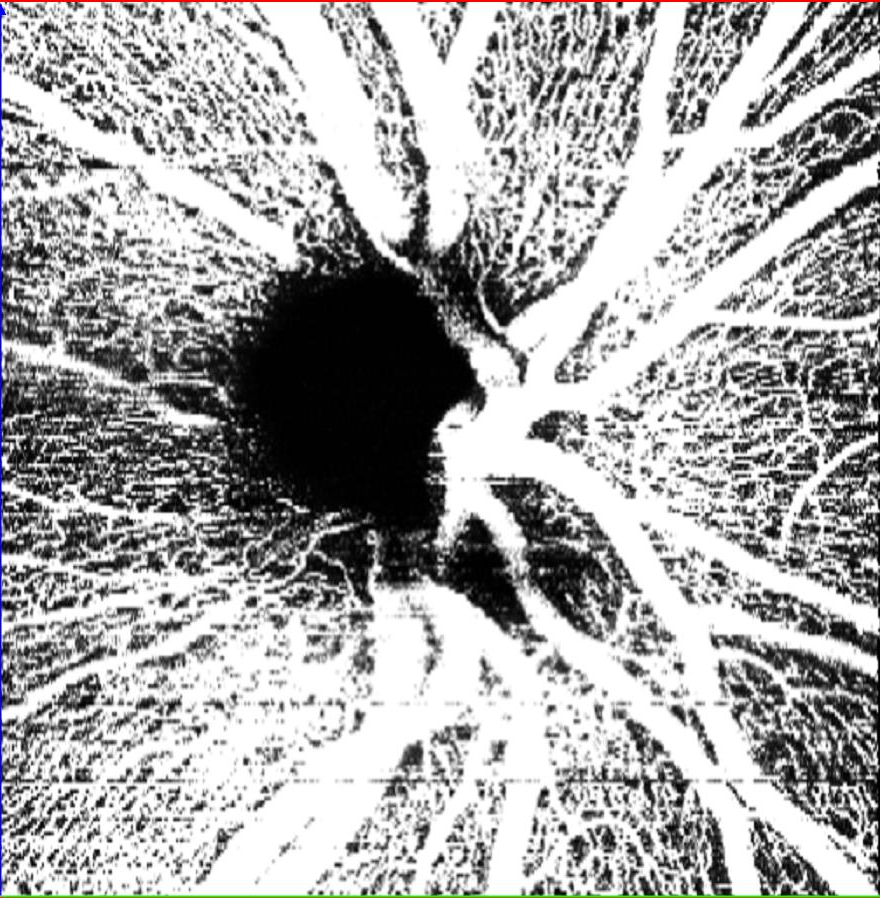

Supplement: S1 File — (ZIP) [file pone.0197588.s001.zip › Data Article Plos/Section repeatability/OCT A 5 appareils/HRA/10.tiff]

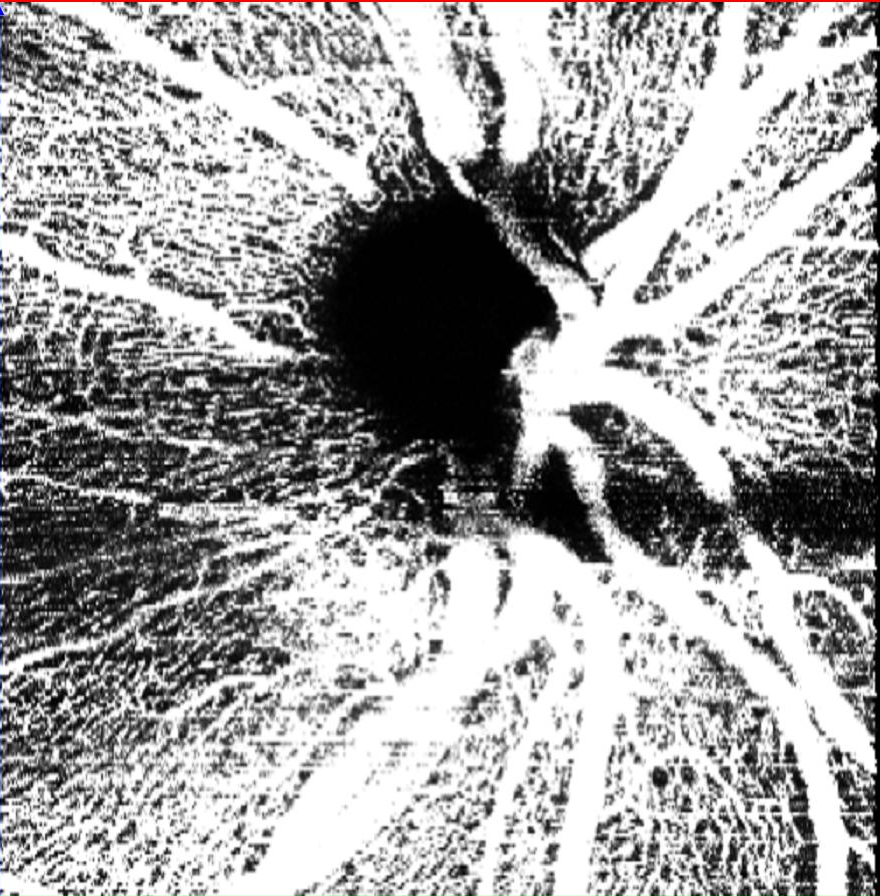

Supplement: S1 File — (ZIP) [file pone.0197588.s001.zip › Data Article Plos/Section repeatability/OCT A 5 appareils/HRA/3.tif]

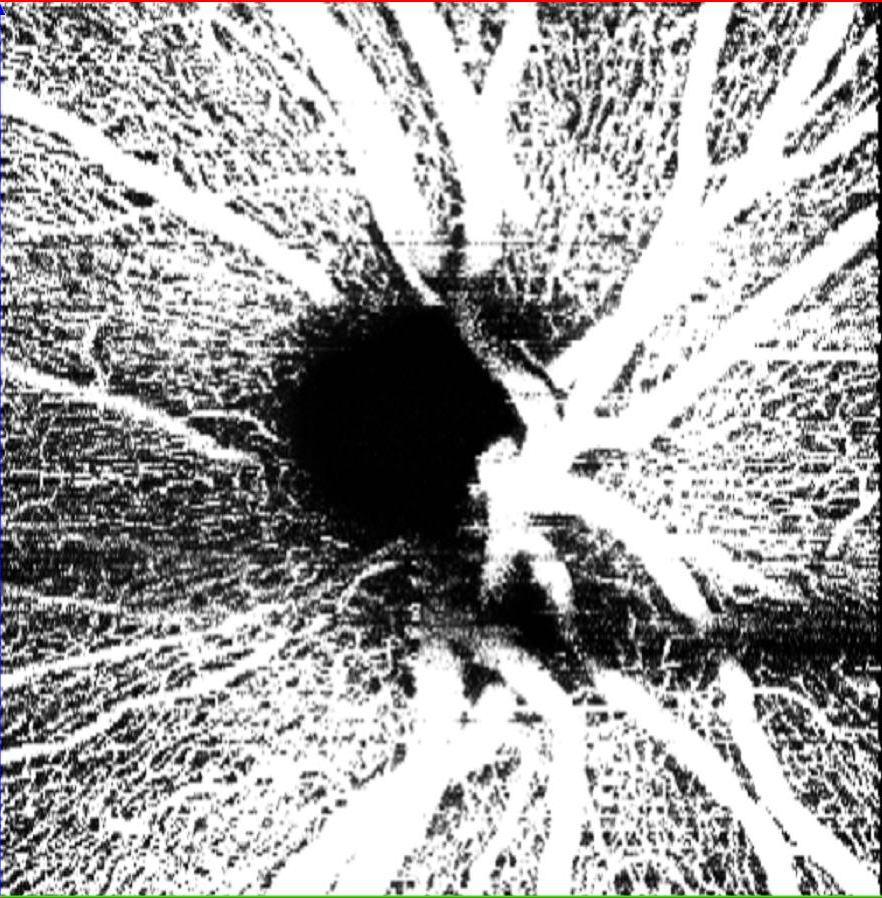

Supplement: S1 File — (ZIP) [file pone.0197588.s001.zip › Data Article Plos/Section repeatability/OCT A 5 appareils/HRA/4.tif]

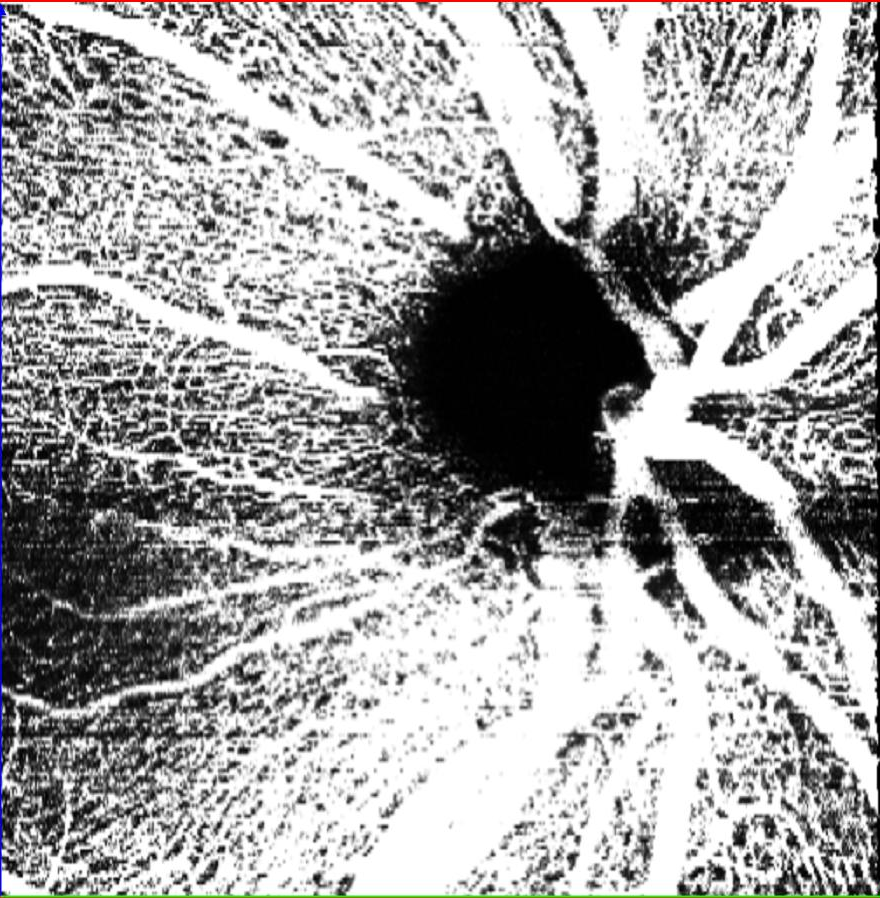

Supplement: S1 File — (ZIP) [file pone.0197588.s001.zip › Data Article Plos/Section repeatability/OCT A 5 appareils/HRA/5.tiff]

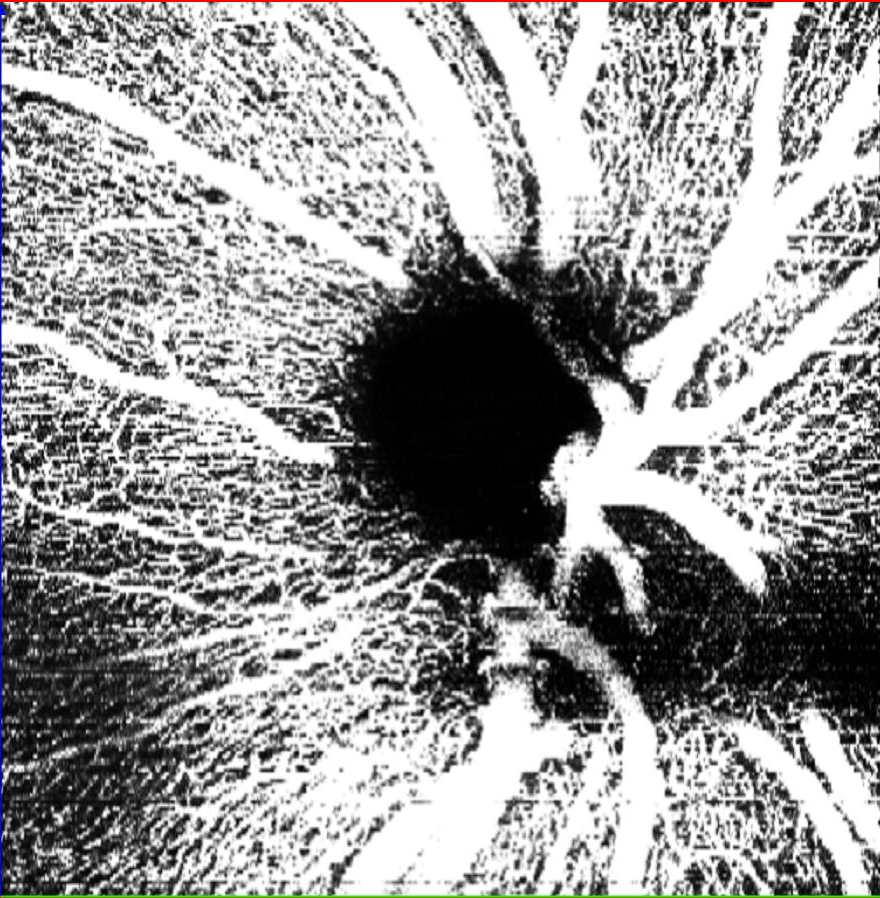

Supplement: S1 File — (ZIP) [file pone.0197588.s001.zip › Data Article Plos/Section repeatability/OCT A 5 appareils/HRA/6.tiff]

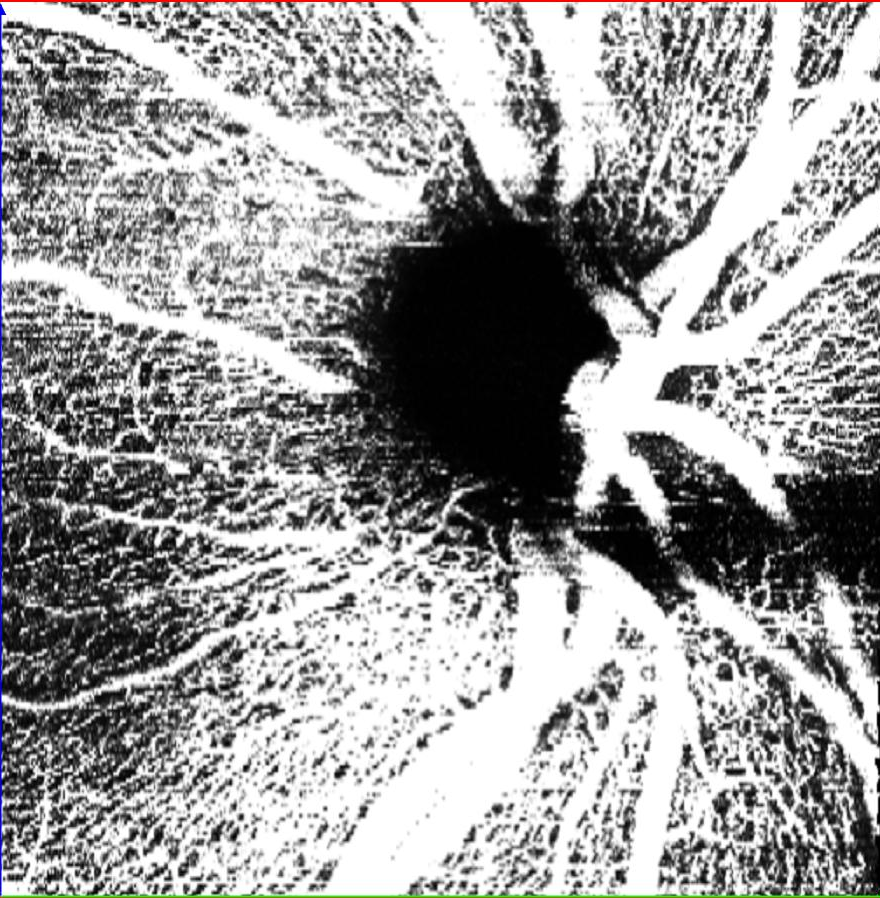

Supplement: S1 File — (ZIP) [file pone.0197588.s001.zip › Data Article Plos/Section repeatability/OCT A 5 appareils/HRA/7.tiff]

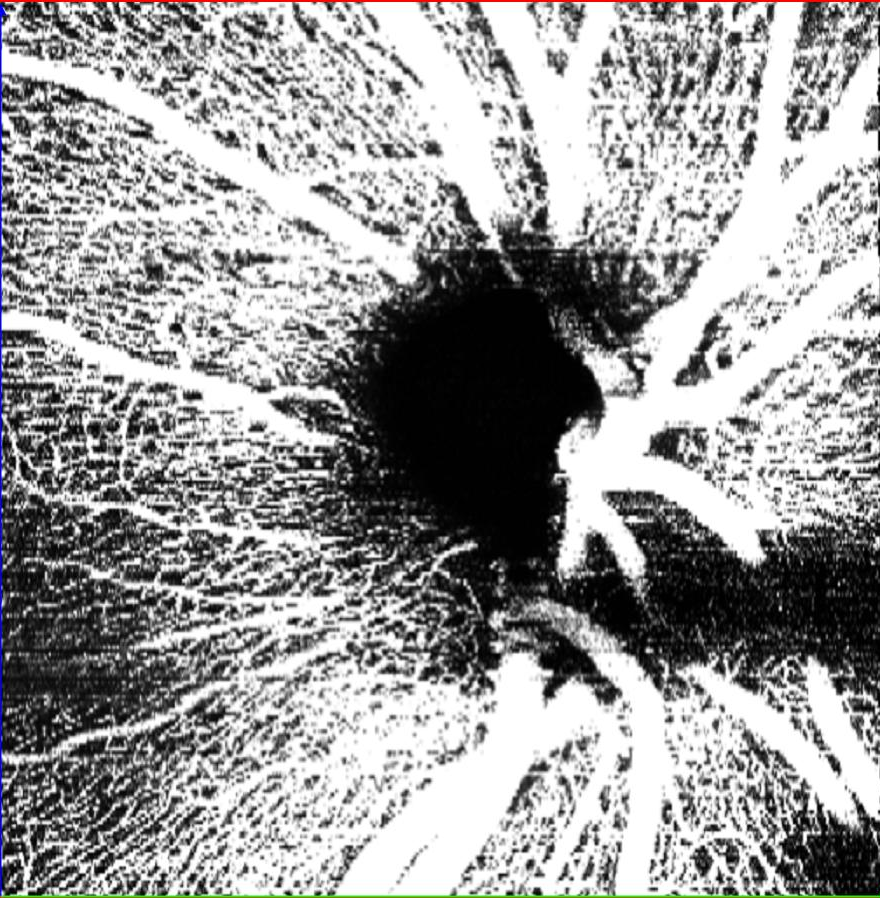

Supplement: S1 File — (ZIP) [file pone.0197588.s001.zip › Data Article Plos/Section repeatability/OCT A 5 appareils/HRA/8.tiff]

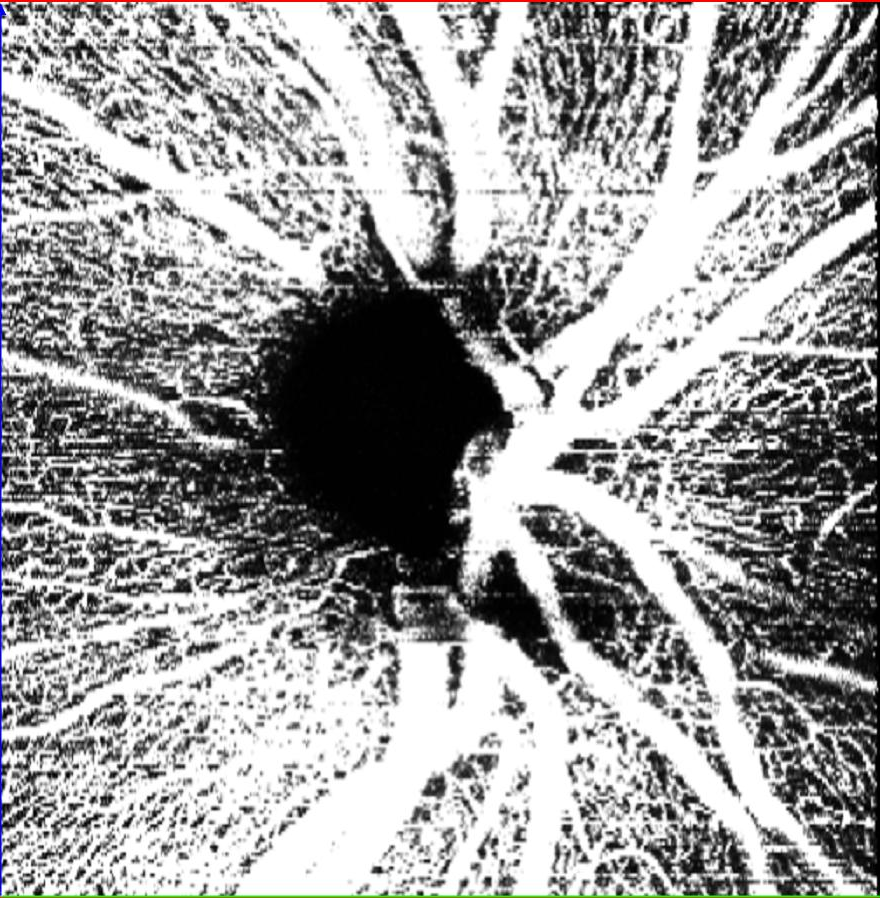

Supplement: S1 File — (ZIP) [file pone.0197588.s001.zip › Data Article Plos/Section repeatability/OCT A 5 appareils/HRA/9.tiff]

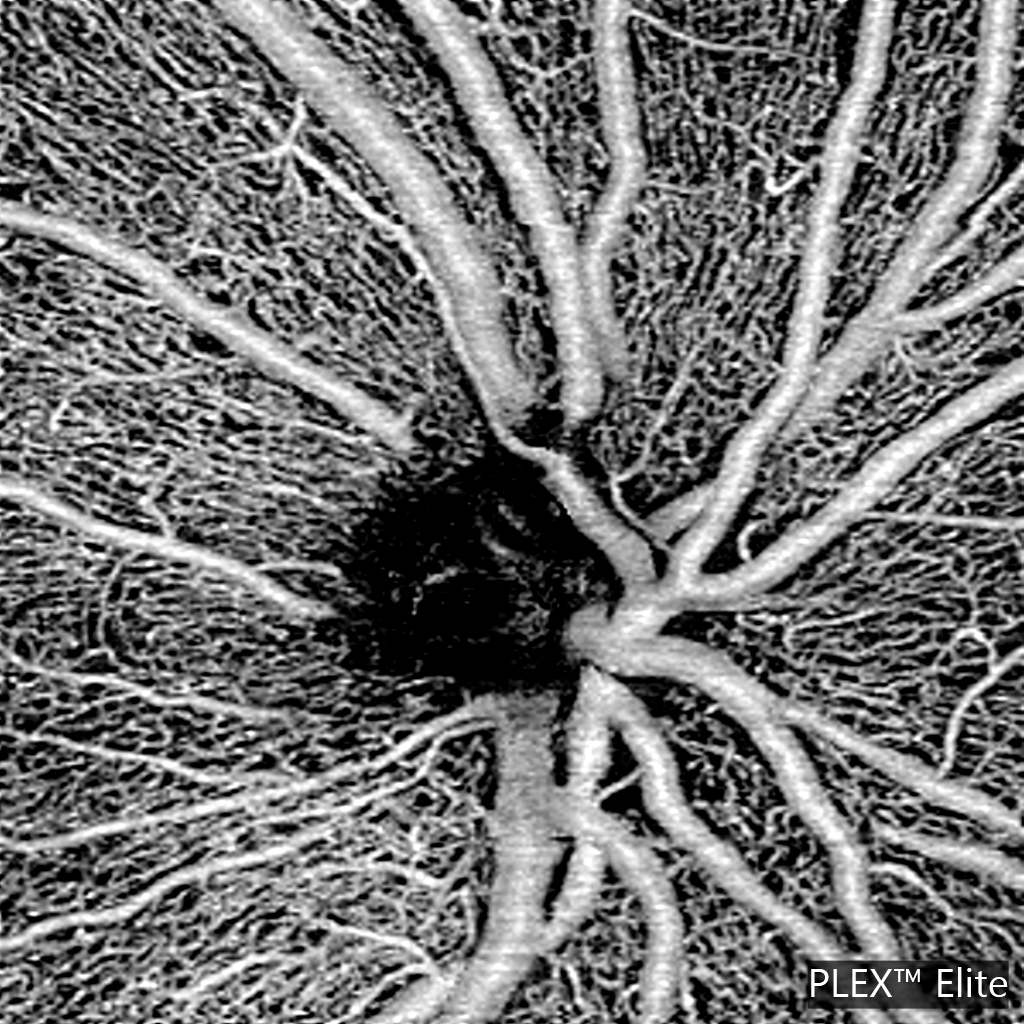

Supplement: S1 File — (ZIP) [file pone.0197588.s001.zip › Data Article Plos/Section repeatability/OCT A 5 appareils/Plex Elit/1.bmp]

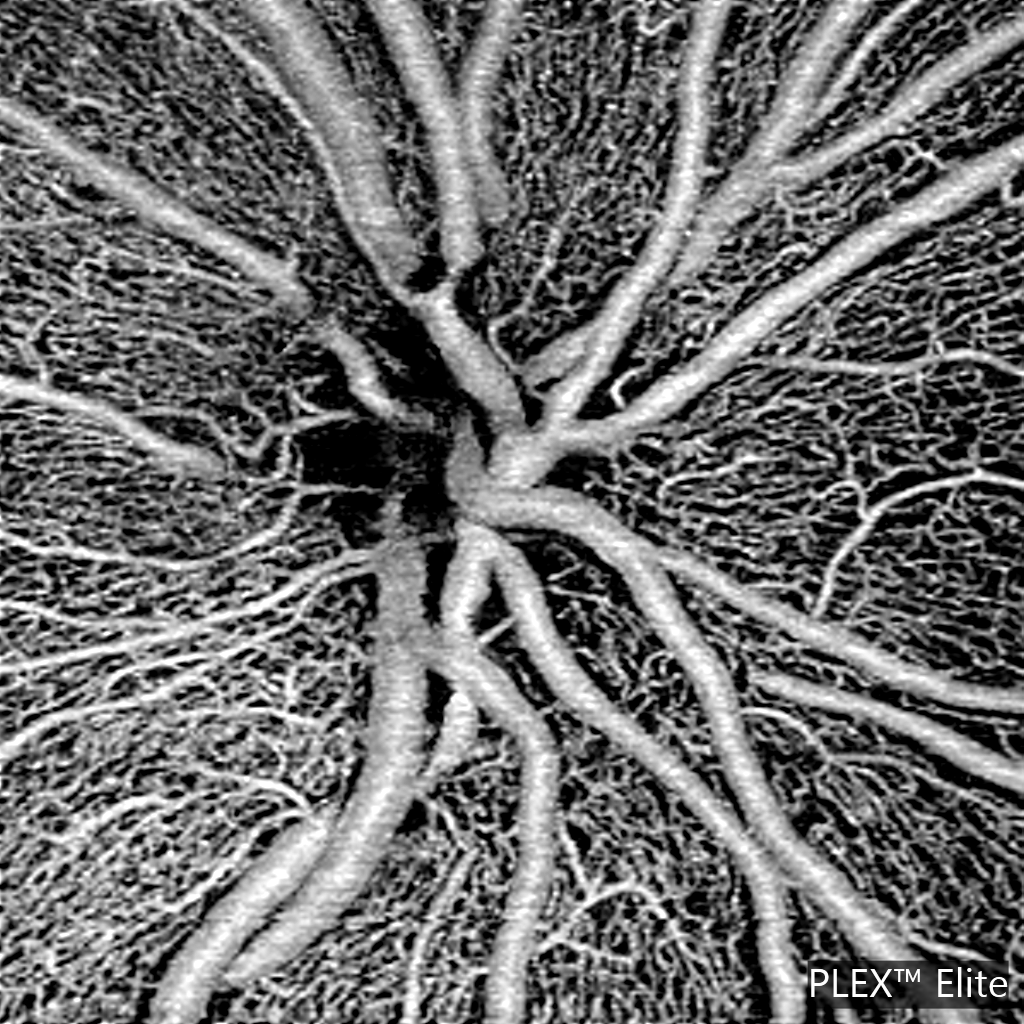

Supplement: S1 File — (ZIP) [file pone.0197588.s001.zip › Data Article Plos/Section repeatability/OCT A 5 appareils/Plex Elit/10.bmp]

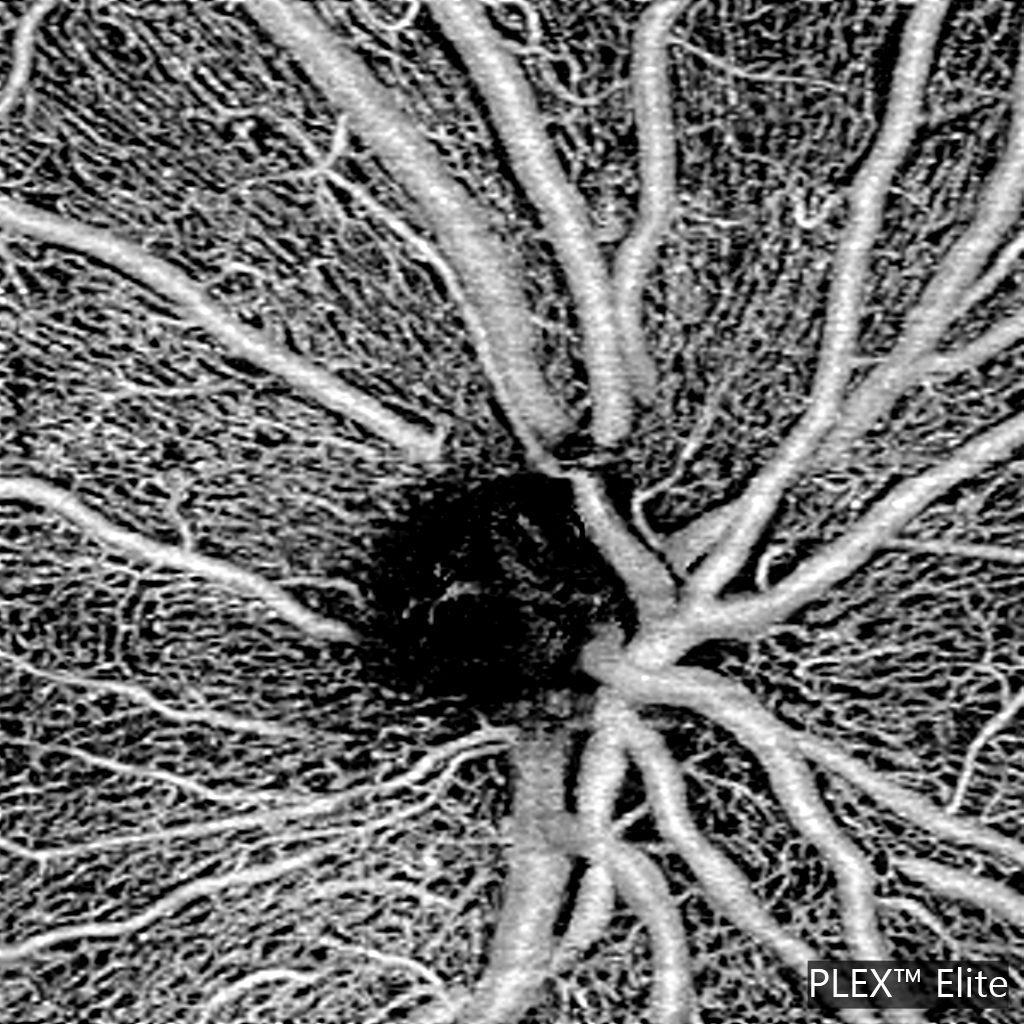

Supplement: S1 File — (ZIP) [file pone.0197588.s001.zip › Data Article Plos/Section repeatability/OCT A 5 appareils/Plex Elit/2.bmp]

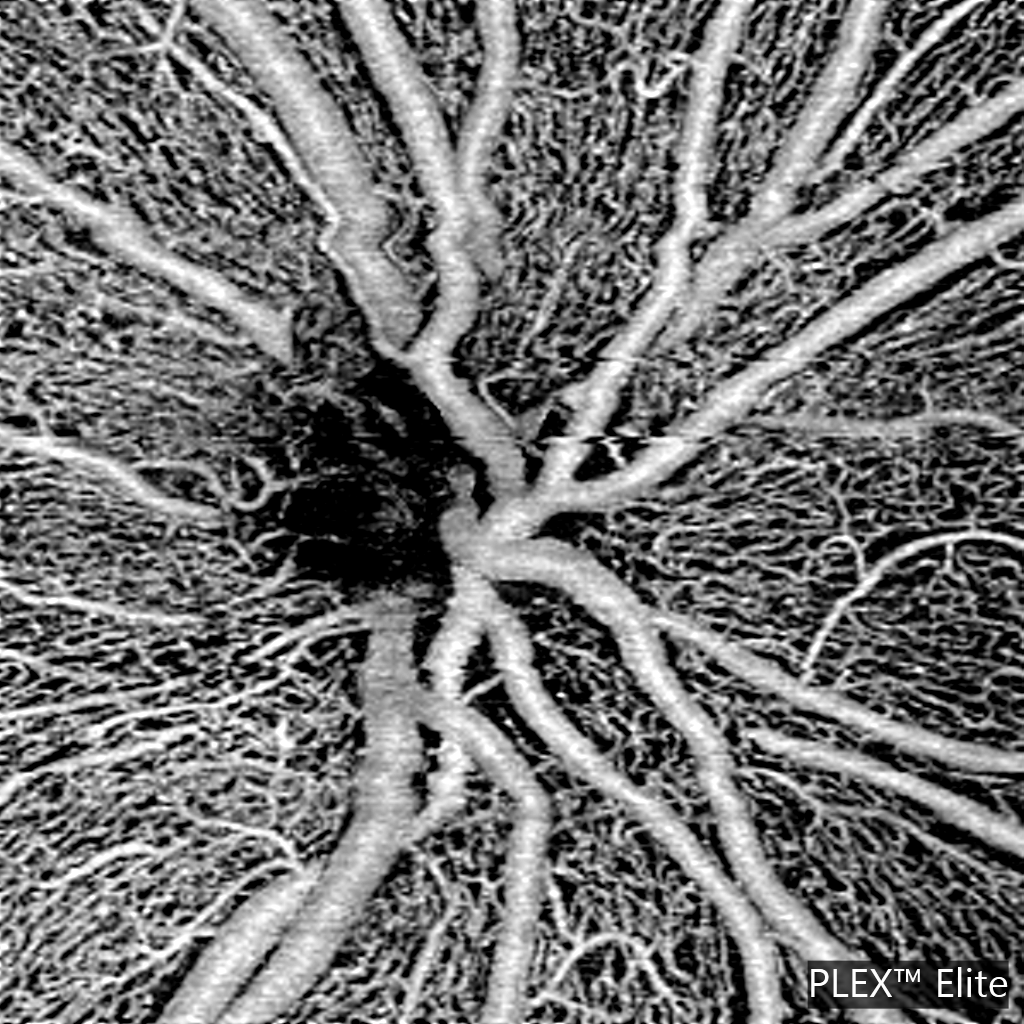

Supplement: S1 File — (ZIP) [file pone.0197588.s001.zip › Data Article Plos/Section repeatability/OCT A 5 appareils/Plex Elit/3.bmp]

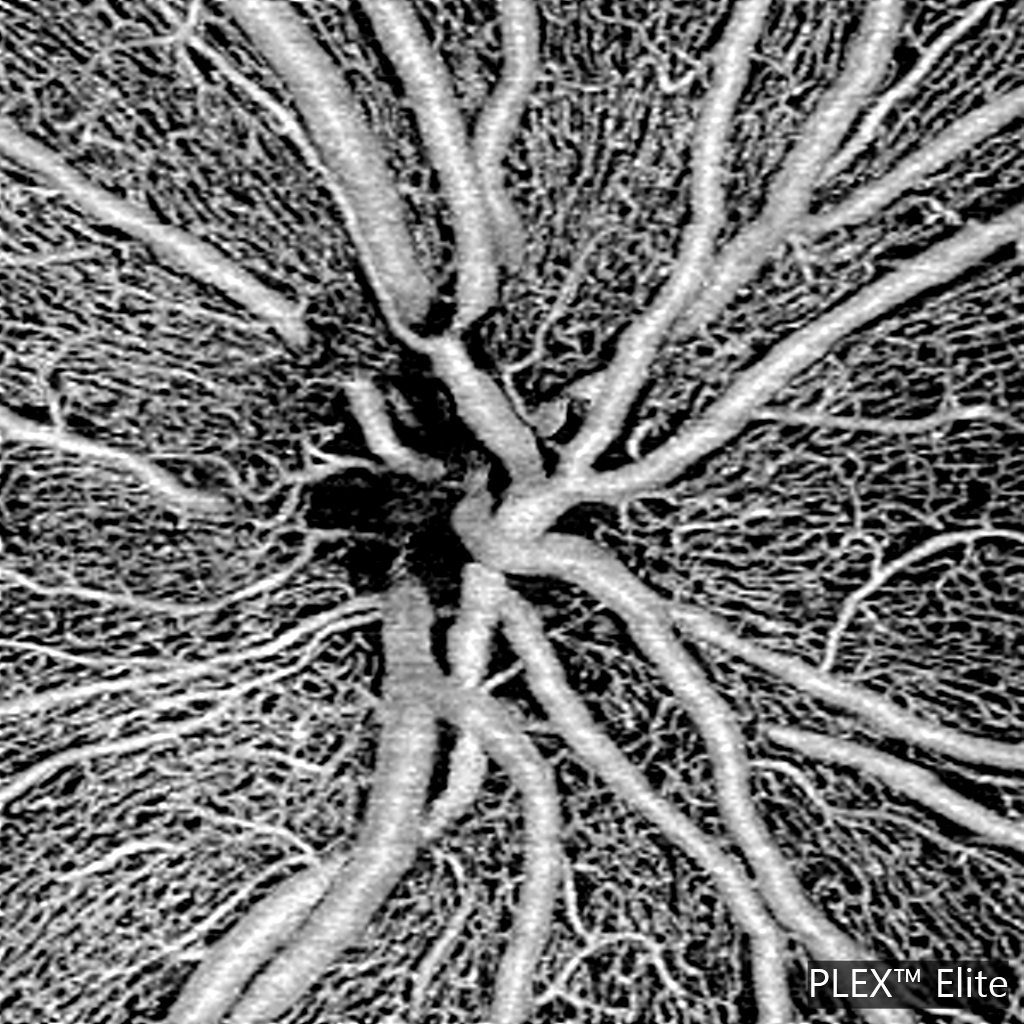

Supplement: S1 File — (ZIP) [file pone.0197588.s001.zip › Data Article Plos/Section repeatability/OCT A 5 appareils/Plex Elit/4.bmp]

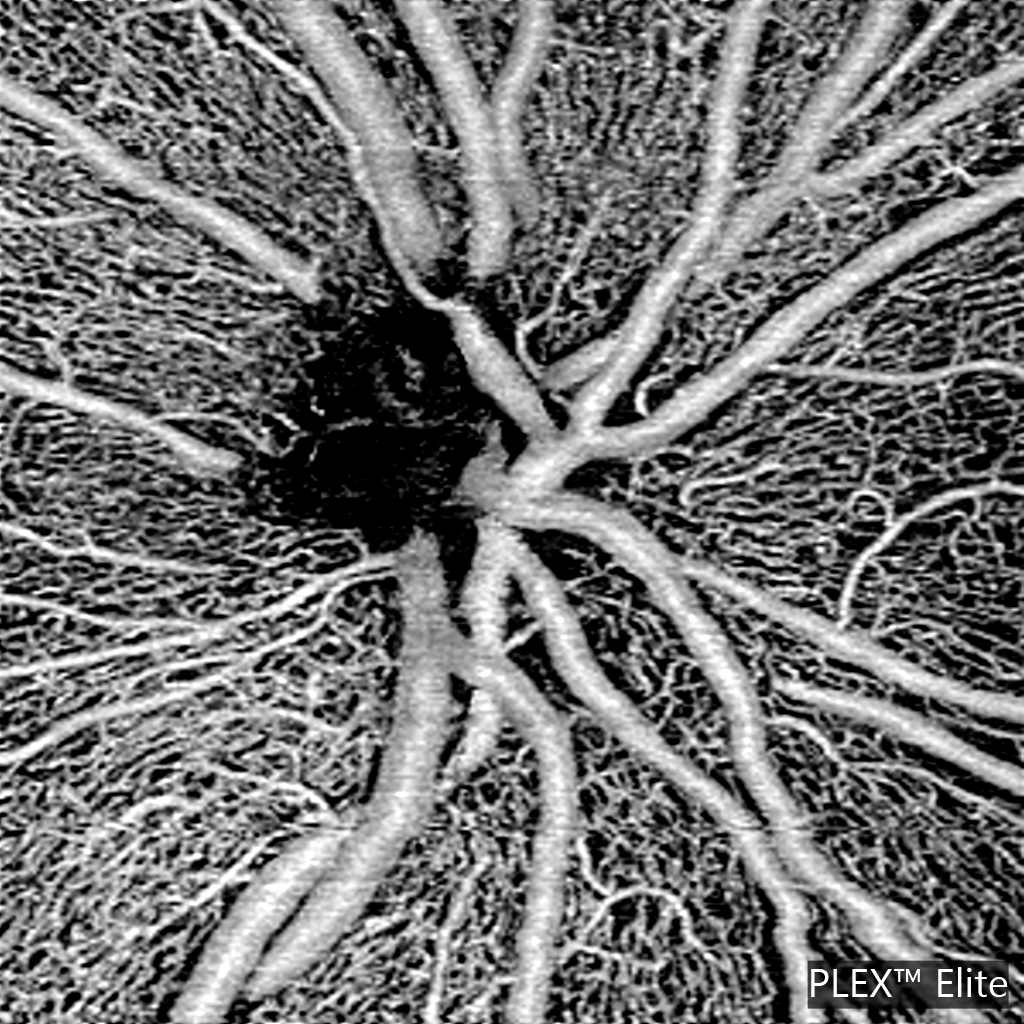

Supplement: S1 File — (ZIP) [file pone.0197588.s001.zip › Data Article Plos/Section repeatability/OCT A 5 appareils/Plex Elit/5.bmp]

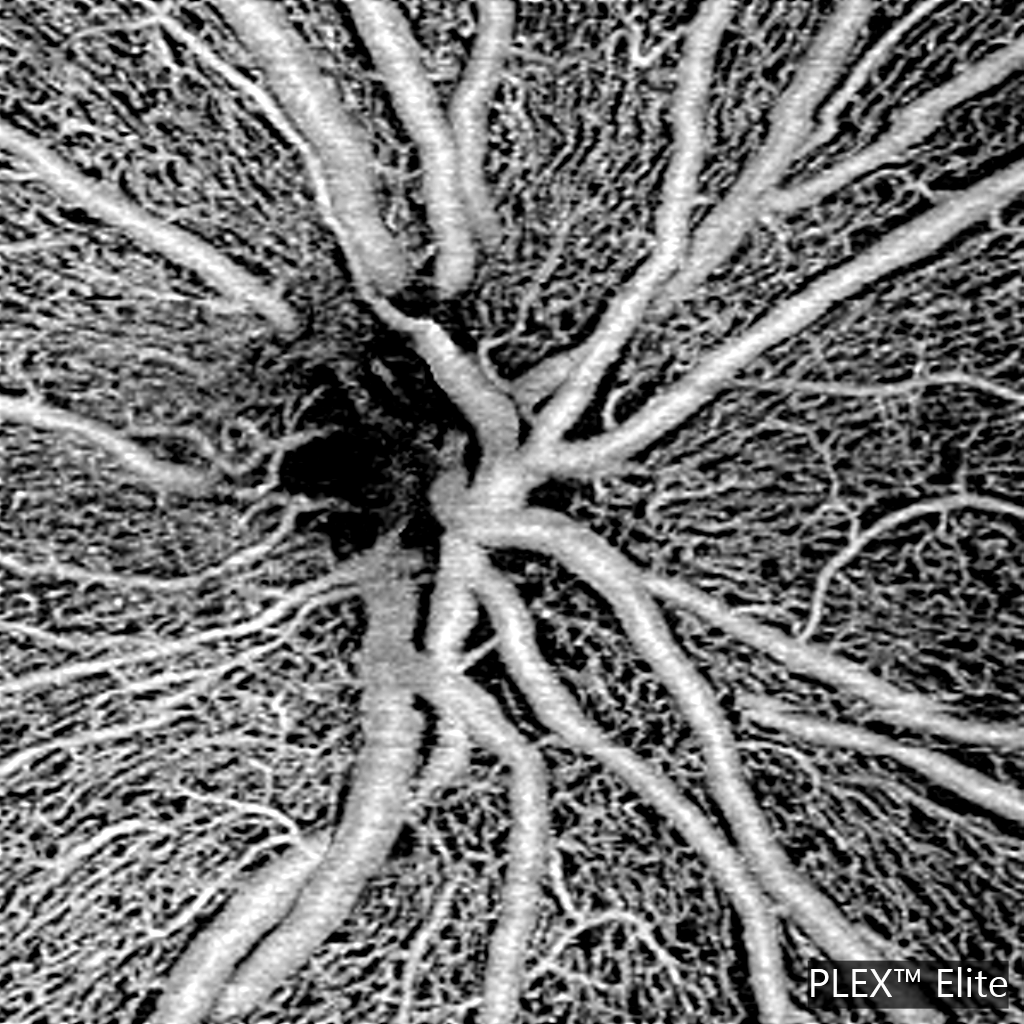

Supplement: S1 File — (ZIP) [file pone.0197588.s001.zip › Data Article Plos/Section repeatability/OCT A 5 appareils/Plex Elit/6.bmp]

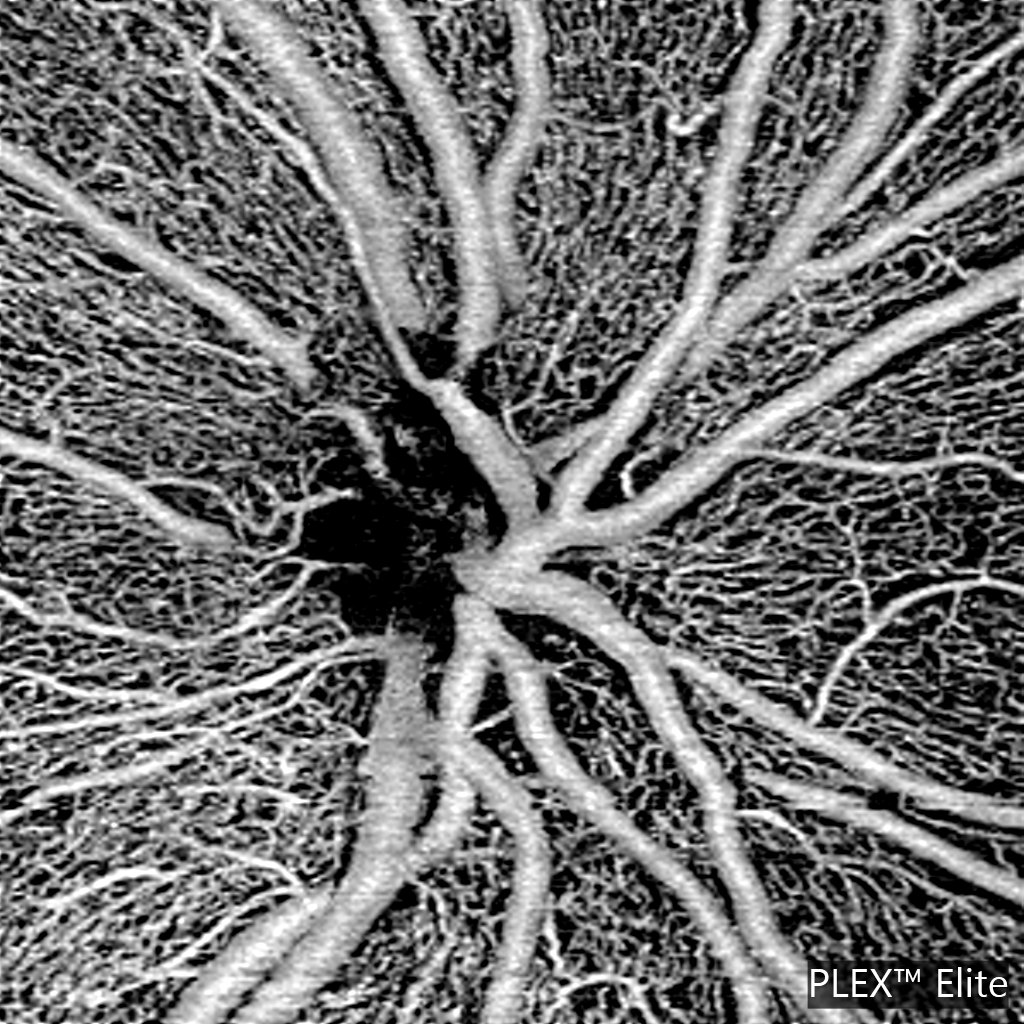

Supplement: S1 File — (ZIP) [file pone.0197588.s001.zip › Data Article Plos/Section repeatability/OCT A 5 appareils/Plex Elit/7.bmp]

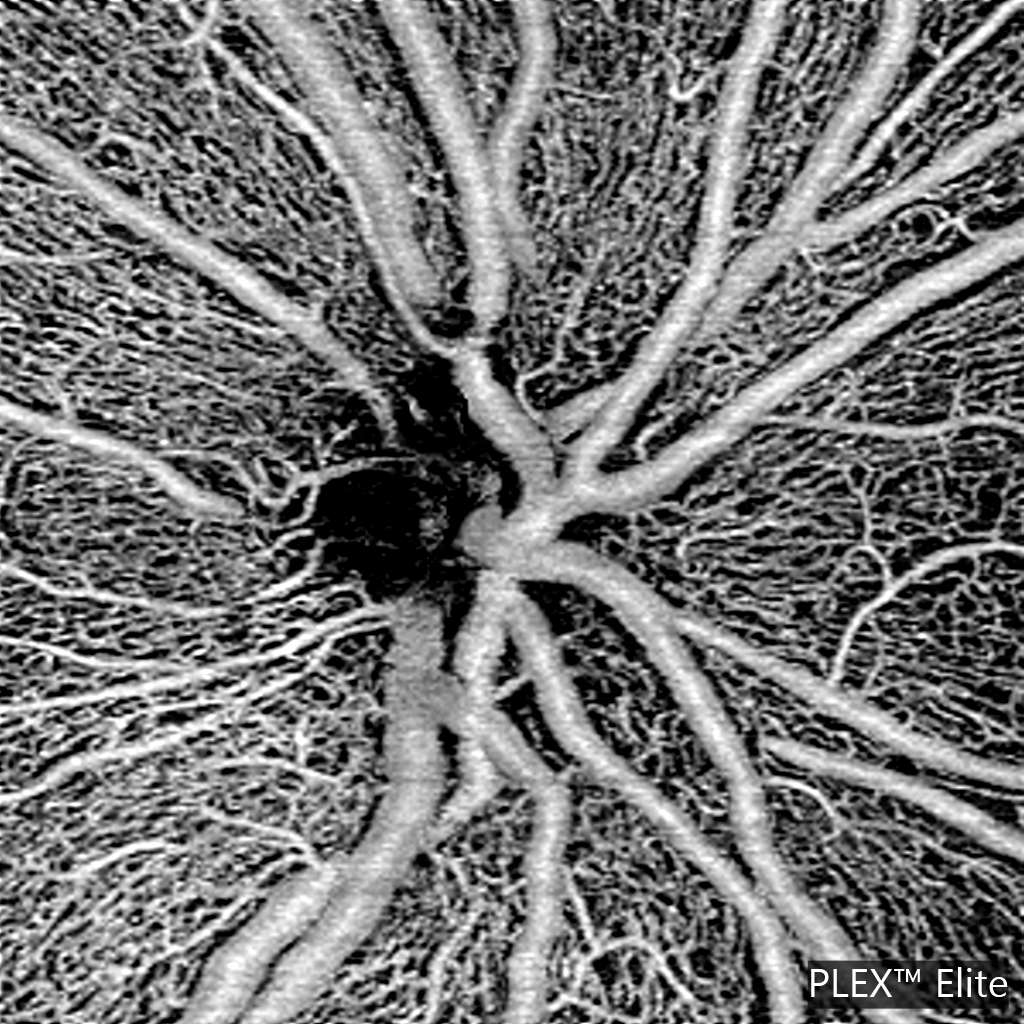

Supplement: S1 File — (ZIP) [file pone.0197588.s001.zip › Data Article Plos/Section repeatability/OCT A 5 appareils/Plex Elit/8.bmp]

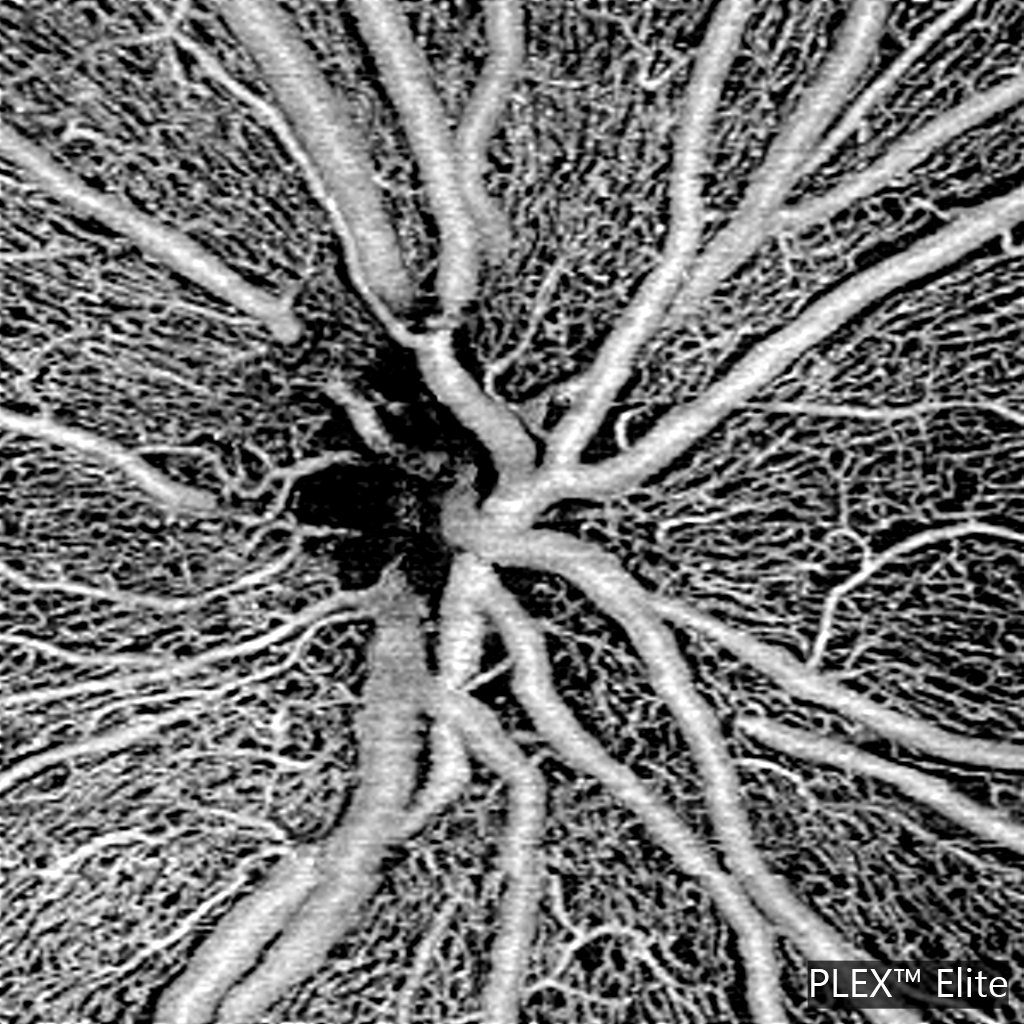

Supplement: S1 File — (ZIP) [file pone.0197588.s001.zip › Data Article Plos/Section repeatability/OCT A 5 appareils/Plex Elit/9.bmp]

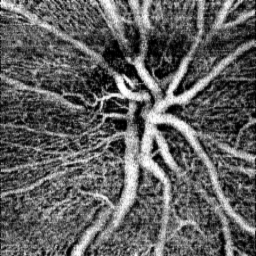

Supplement: S1 File — (ZIP) [file pone.0197588.s001.zip › Data Article Plos/Section repeatability/OCT A 5 appareils/Topcon/1.jpg]
