# Supplementary figures and images for: Toward quantitative and reproducible clinical use of OCT-Angiography (part 1 of 2)
Source: PLoS One. 2018 Jul 6;13(7):e0197588. doi: 10.1371/journal.pone.0197588 (PMC6034792; doi:10.1371/journal.pone.0197588)

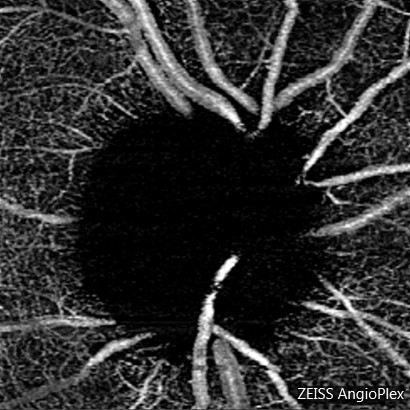

Supplement: S1 File — (ZIP) [file pone.0197588.s001.zip › Data Article Plos/Section application/Deep Network/Stade 1/1.jpg]

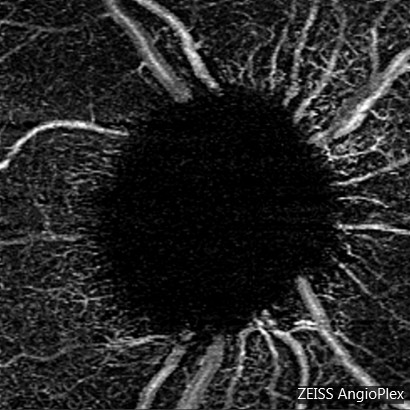

Supplement: S1 File — (ZIP) [file pone.0197588.s001.zip › Data Article Plos/Section application/Deep Network/Stade 1/10.jpg]

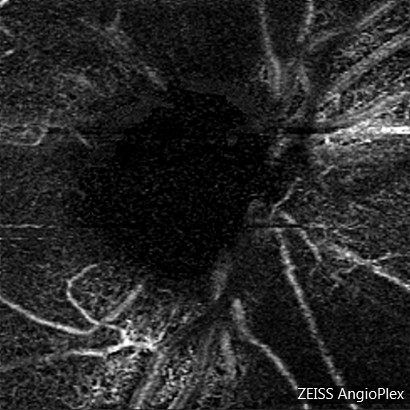

Supplement: S1 File — (ZIP) [file pone.0197588.s001.zip › Data Article Plos/Section application/Deep Network/Stade 1/11.jpg]

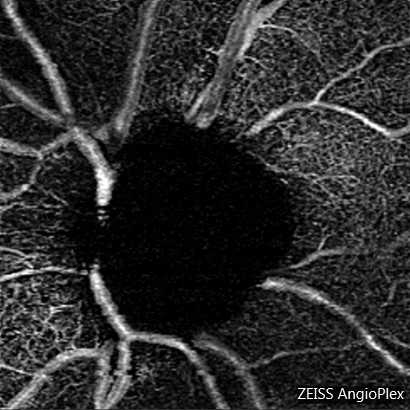

Supplement: S1 File — (ZIP) [file pone.0197588.s001.zip › Data Article Plos/Section application/Deep Network/Stade 1/12.jpg]

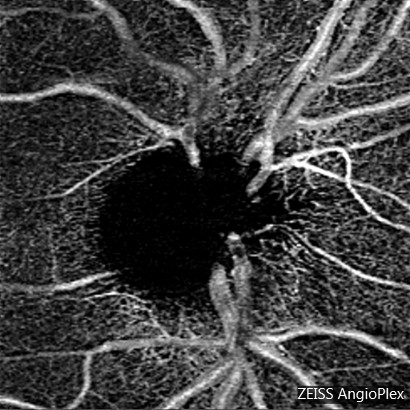

Supplement: S1 File — (ZIP) [file pone.0197588.s001.zip › Data Article Plos/Section application/Deep Network/Stade 1/13.jpg]

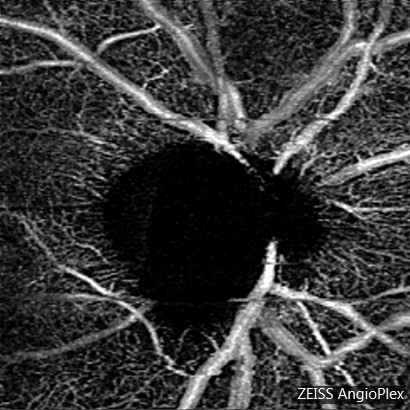

Supplement: S1 File — (ZIP) [file pone.0197588.s001.zip › Data Article Plos/Section application/Deep Network/Stade 1/14.jpg]

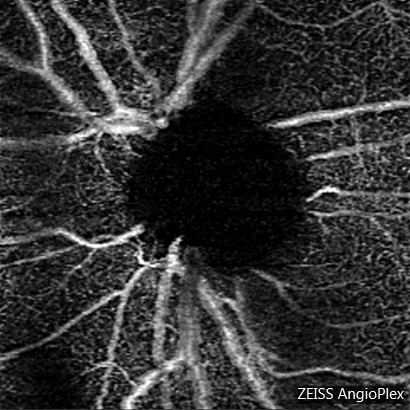

Supplement: S1 File — (ZIP) [file pone.0197588.s001.zip › Data Article Plos/Section application/Deep Network/Stade 1/15.jpg]

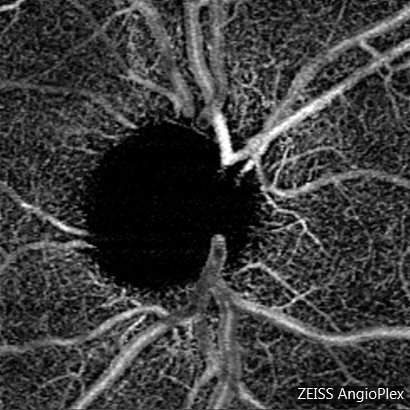

Supplement: S1 File — (ZIP) [file pone.0197588.s001.zip › Data Article Plos/Section application/Deep Network/Stade 1/16.jpg]

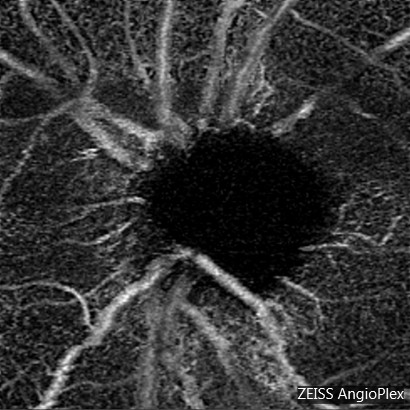

Supplement: S1 File — (ZIP) [file pone.0197588.s001.zip › Data Article Plos/Section application/Deep Network/Stade 1/17.jpg]

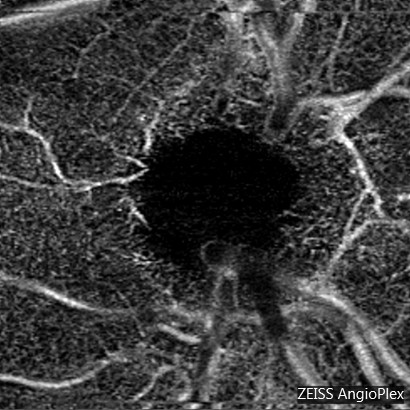

Supplement: S1 File — (ZIP) [file pone.0197588.s001.zip › Data Article Plos/Section application/Deep Network/Stade 1/18.jpg]

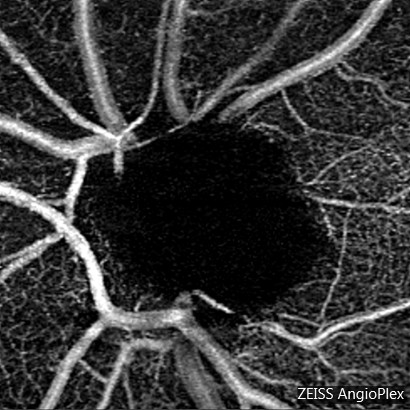

Supplement: S1 File — (ZIP) [file pone.0197588.s001.zip › Data Article Plos/Section application/Deep Network/Stade 1/19.jpg]

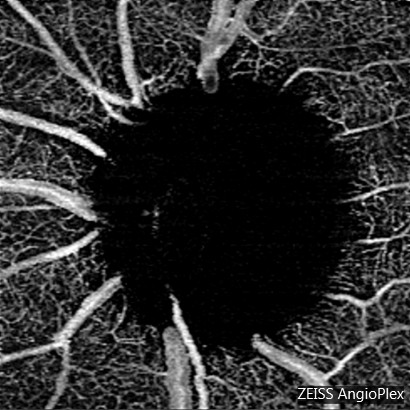

Supplement: S1 File — (ZIP) [file pone.0197588.s001.zip › Data Article Plos/Section application/Deep Network/Stade 1/2.jpg]

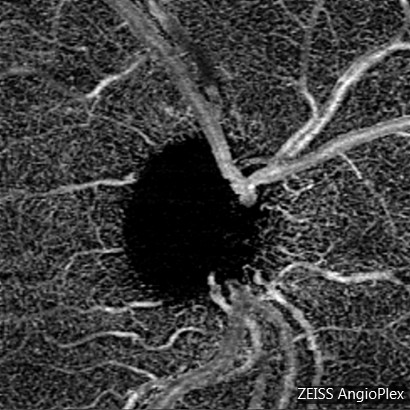

Supplement: S1 File — (ZIP) [file pone.0197588.s001.zip › Data Article Plos/Section application/Deep Network/Stade 1/20.jpg]

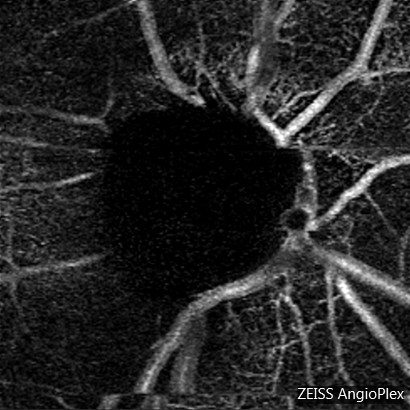

Supplement: S1 File — (ZIP) [file pone.0197588.s001.zip › Data Article Plos/Section application/Deep Network/Stade 1/3.jpg]

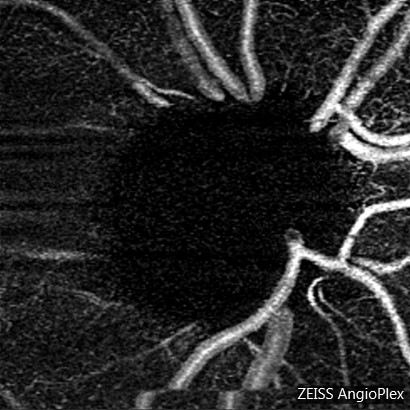

Supplement: S1 File — (ZIP) [file pone.0197588.s001.zip › Data Article Plos/Section application/Deep Network/Stade 1/4.jpg]

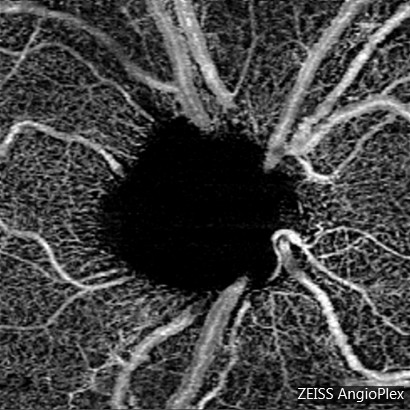

Supplement: S1 File — (ZIP) [file pone.0197588.s001.zip › Data Article Plos/Section application/Deep Network/Stade 1/5.jpg]

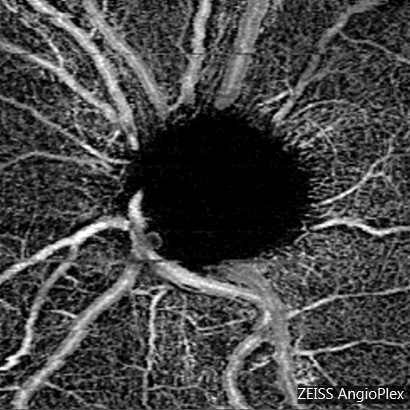

Supplement: S1 File — (ZIP) [file pone.0197588.s001.zip › Data Article Plos/Section application/Deep Network/Stade 1/6.jpg]

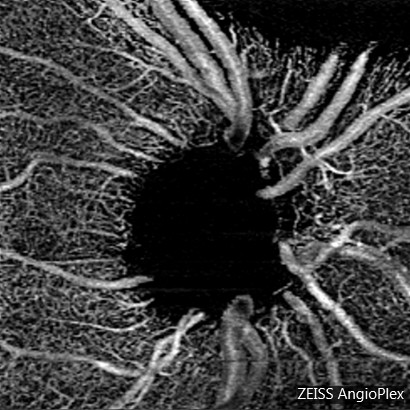

Supplement: S1 File — (ZIP) [file pone.0197588.s001.zip › Data Article Plos/Section application/Deep Network/Stade 1/7.jpg]

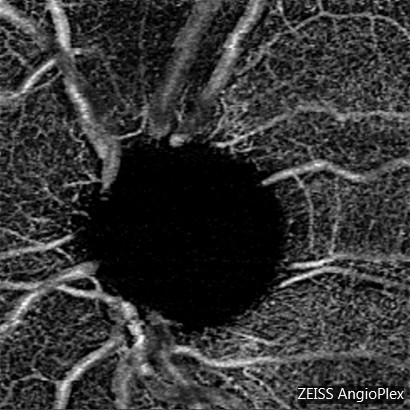

Supplement: S1 File — (ZIP) [file pone.0197588.s001.zip › Data Article Plos/Section application/Deep Network/Stade 1/8.jpg]

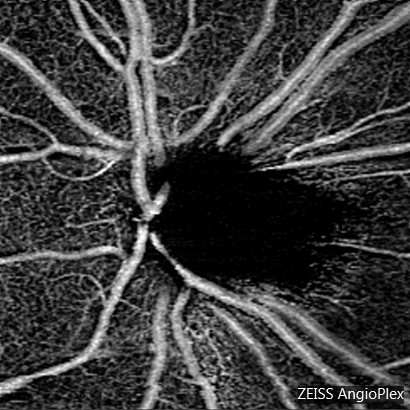

Supplement: S1 File — (ZIP) [file pone.0197588.s001.zip › Data Article Plos/Section application/Deep Network/Stade 1/9.jpg]

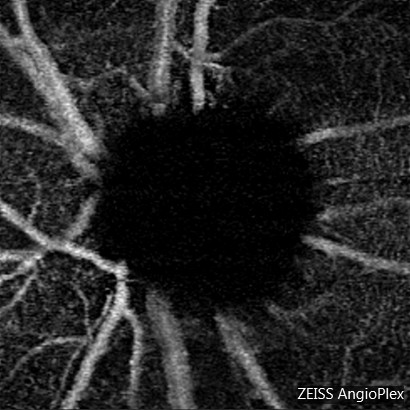

Supplement: S1 File — (ZIP) [file pone.0197588.s001.zip › Data Article Plos/Section application/Deep Network/Stade 2/1.jpg]

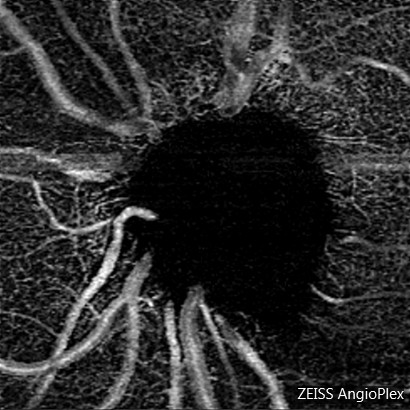

Supplement: S1 File — (ZIP) [file pone.0197588.s001.zip › Data Article Plos/Section application/Deep Network/Stade 2/10.jpg]

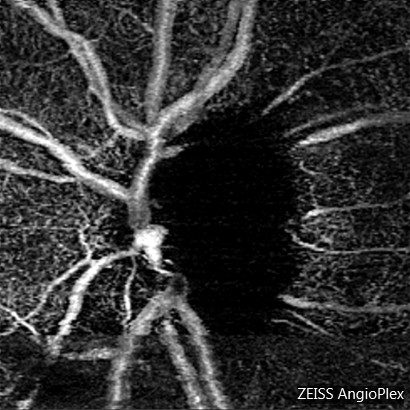

Supplement: S1 File — (ZIP) [file pone.0197588.s001.zip › Data Article Plos/Section application/Deep Network/Stade 2/11.jpg]

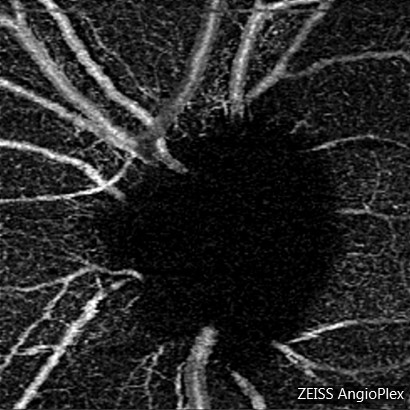

Supplement: S1 File — (ZIP) [file pone.0197588.s001.zip › Data Article Plos/Section application/Deep Network/Stade 2/12.jpg]

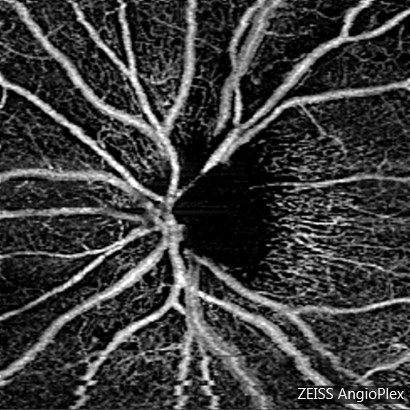

Supplement: S1 File — (ZIP) [file pone.0197588.s001.zip › Data Article Plos/Section application/Deep Network/Stade 2/13.jpg]

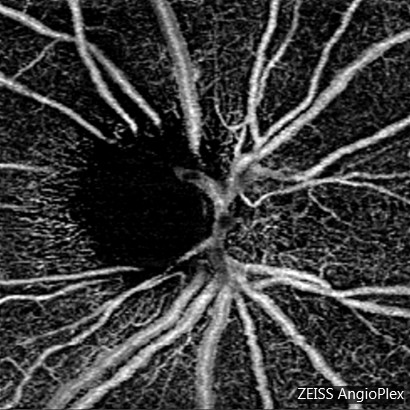

Supplement: S1 File — (ZIP) [file pone.0197588.s001.zip › Data Article Plos/Section application/Deep Network/Stade 2/14.jpg]

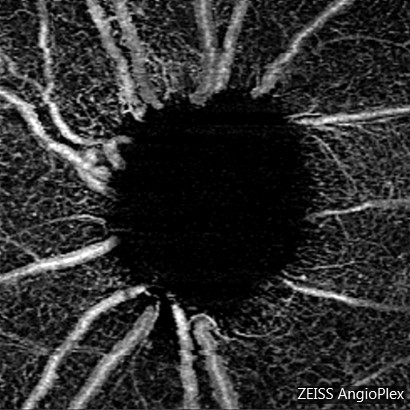

Supplement: S1 File — (ZIP) [file pone.0197588.s001.zip › Data Article Plos/Section application/Deep Network/Stade 2/15.jpg]

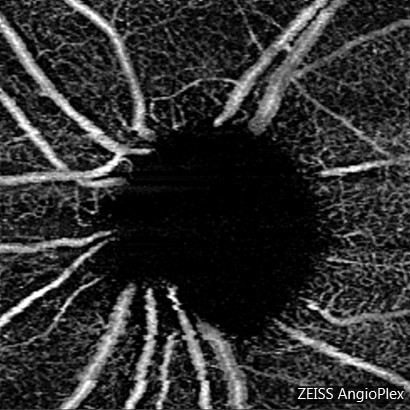

Supplement: S1 File — (ZIP) [file pone.0197588.s001.zip › Data Article Plos/Section application/Deep Network/Stade 2/16.jpg]

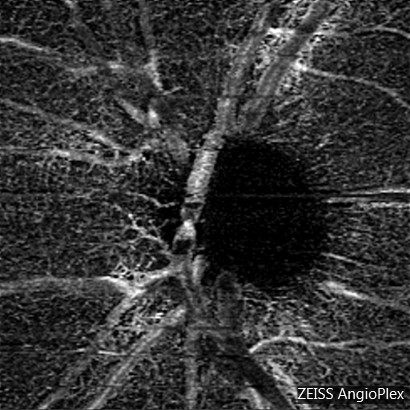

Supplement: S1 File — (ZIP) [file pone.0197588.s001.zip › Data Article Plos/Section application/Deep Network/Stade 2/17.jpg]

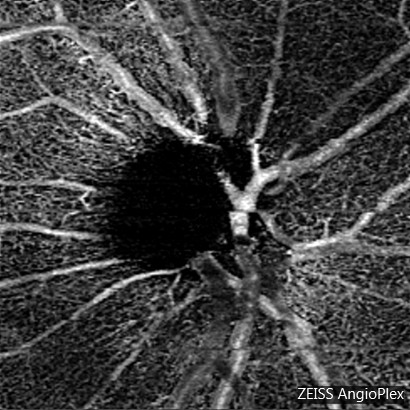

Supplement: S1 File — (ZIP) [file pone.0197588.s001.zip › Data Article Plos/Section application/Deep Network/Stade 2/18.jpg]

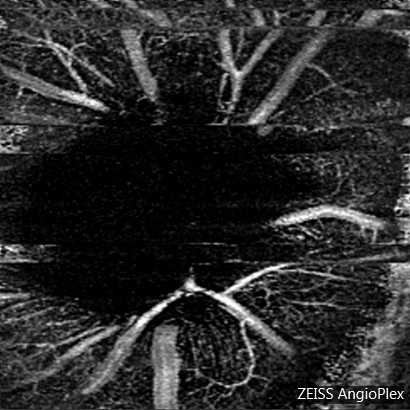

Supplement: S1 File — (ZIP) [file pone.0197588.s001.zip › Data Article Plos/Section application/Deep Network/Stade 2/19.jpg]

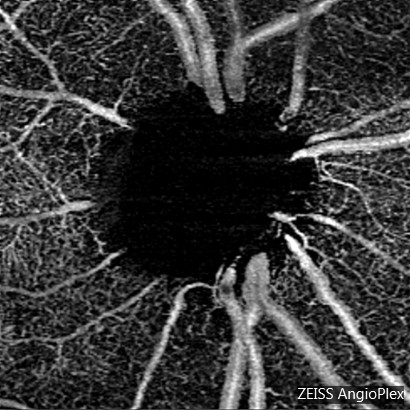

Supplement: S1 File — (ZIP) [file pone.0197588.s001.zip › Data Article Plos/Section application/Deep Network/Stade 2/2.jpg]

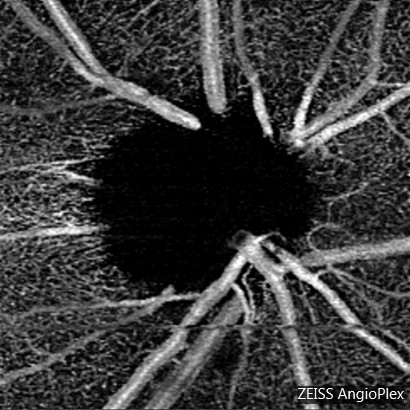

Supplement: S1 File — (ZIP) [file pone.0197588.s001.zip › Data Article Plos/Section application/Deep Network/Stade 2/20.jpg]

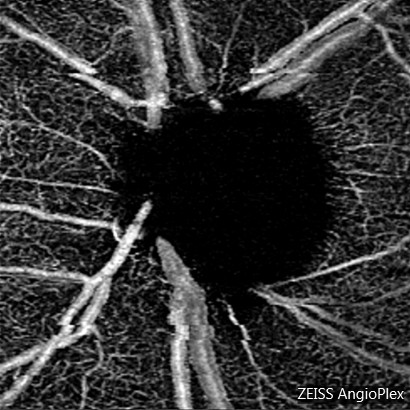

Supplement: S1 File — (ZIP) [file pone.0197588.s001.zip › Data Article Plos/Section application/Deep Network/Stade 2/3.jpg]

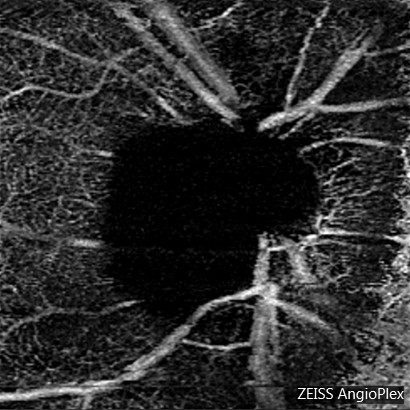

Supplement: S1 File — (ZIP) [file pone.0197588.s001.zip › Data Article Plos/Section application/Deep Network/Stade 2/4.jpg]

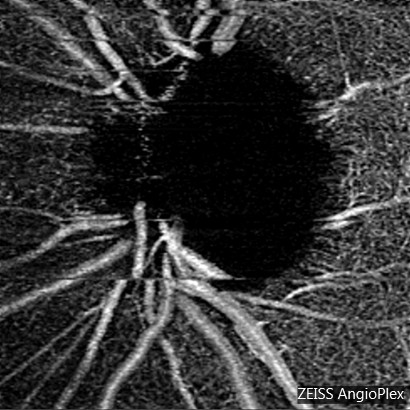

Supplement: S1 File — (ZIP) [file pone.0197588.s001.zip › Data Article Plos/Section application/Deep Network/Stade 2/5.jpg]

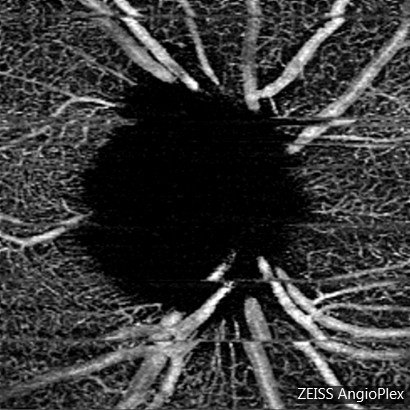

Supplement: S1 File — (ZIP) [file pone.0197588.s001.zip › Data Article Plos/Section application/Deep Network/Stade 2/6.jpg]

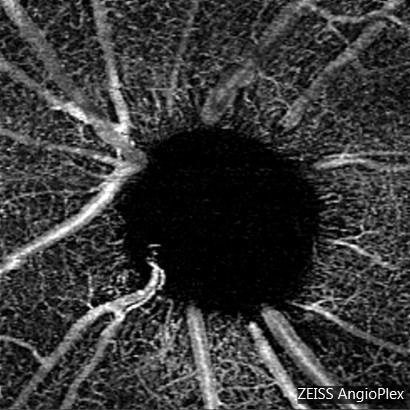

Supplement: S1 File — (ZIP) [file pone.0197588.s001.zip › Data Article Plos/Section application/Deep Network/Stade 2/7.jpg]

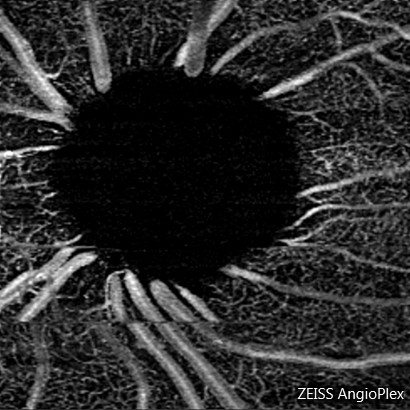

Supplement: S1 File — (ZIP) [file pone.0197588.s001.zip › Data Article Plos/Section application/Deep Network/Stade 2/8.jpg]

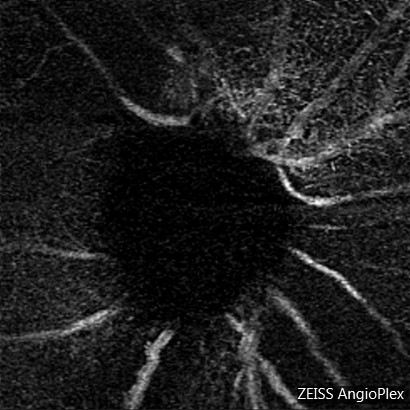

Supplement: S1 File — (ZIP) [file pone.0197588.s001.zip › Data Article Plos/Section application/Deep Network/Stade 2/9.jpg]

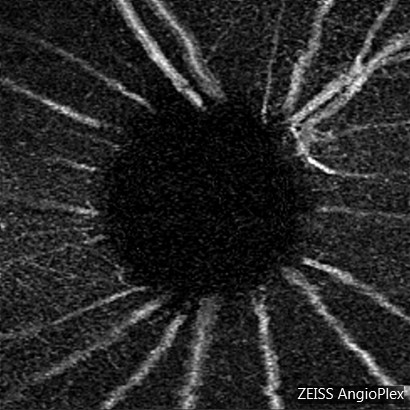

Supplement: S1 File — (ZIP) [file pone.0197588.s001.zip › Data Article Plos/Section application/Deep Network/Stade 3/1.jpg]

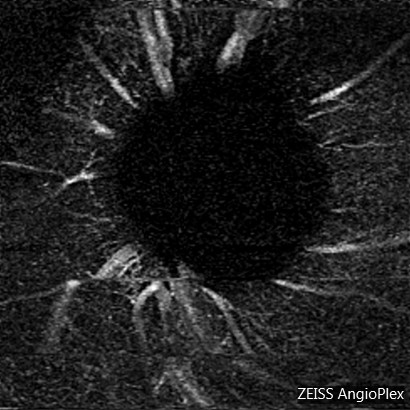

Supplement: S1 File — (ZIP) [file pone.0197588.s001.zip › Data Article Plos/Section application/Deep Network/Stade 3/10.jpg]

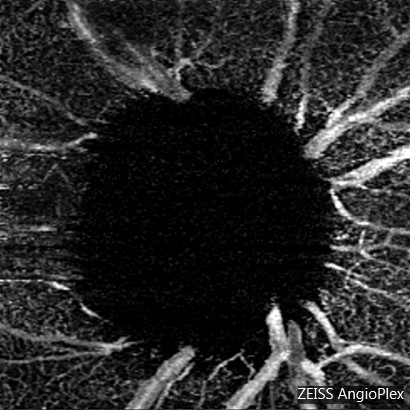

Supplement: S1 File — (ZIP) [file pone.0197588.s001.zip › Data Article Plos/Section application/Deep Network/Stade 3/11.jpg]

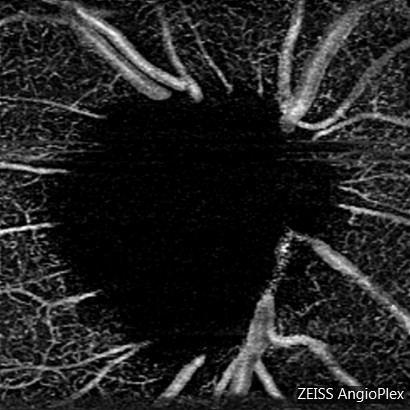

Supplement: S1 File — (ZIP) [file pone.0197588.s001.zip › Data Article Plos/Section application/Deep Network/Stade 3/13.jpg]

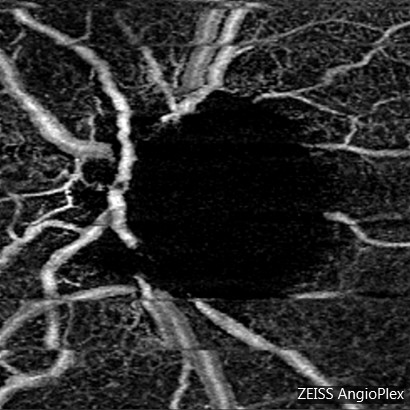

Supplement: S1 File — (ZIP) [file pone.0197588.s001.zip › Data Article Plos/Section application/Deep Network/Stade 3/14.jpg]

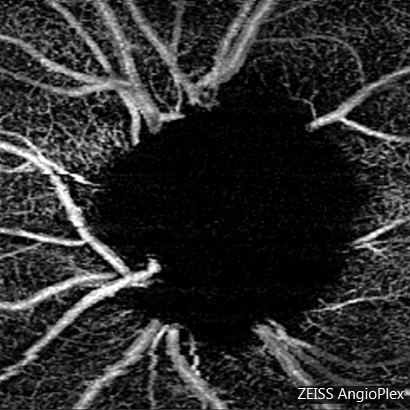

Supplement: S1 File — (ZIP) [file pone.0197588.s001.zip › Data Article Plos/Section application/Deep Network/Stade 3/15.jpg]

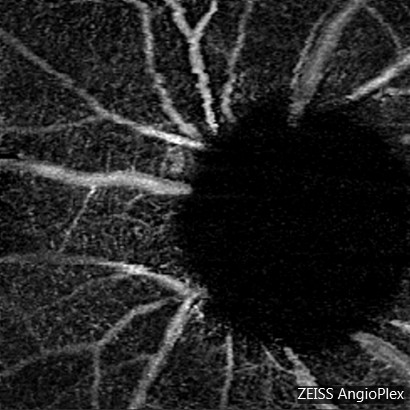

Supplement: S1 File — (ZIP) [file pone.0197588.s001.zip › Data Article Plos/Section application/Deep Network/Stade 3/16.jpg]

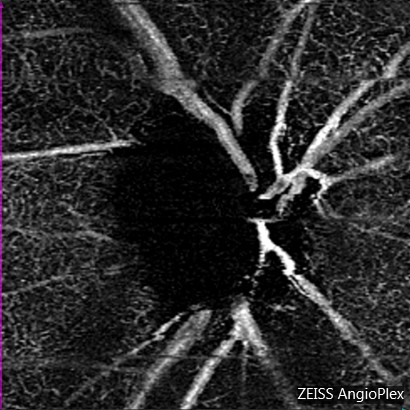

Supplement: S1 File — (ZIP) [file pone.0197588.s001.zip › Data Article Plos/Section application/Deep Network/Stade 3/17.jpg]

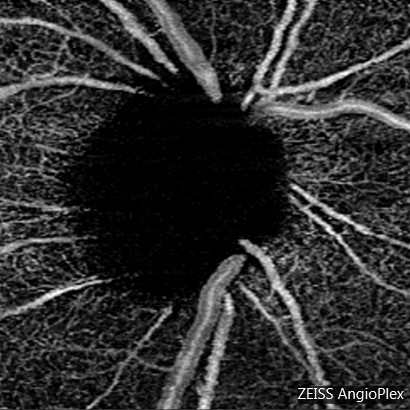

Supplement: S1 File — (ZIP) [file pone.0197588.s001.zip › Data Article Plos/Section application/Deep Network/Stade 3/18.jpg]

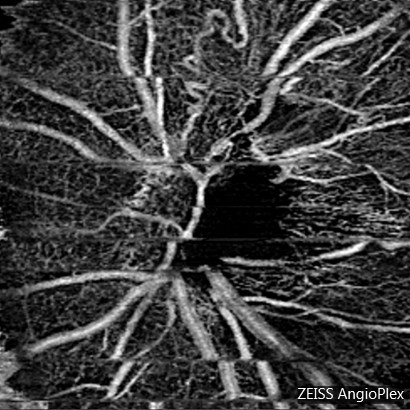

Supplement: S1 File — (ZIP) [file pone.0197588.s001.zip › Data Article Plos/Section application/Deep Network/Stade 3/19.jpg]

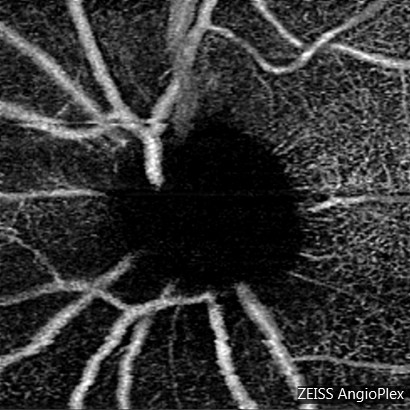

Supplement: S1 File — (ZIP) [file pone.0197588.s001.zip › Data Article Plos/Section application/Deep Network/Stade 3/2.jpg]

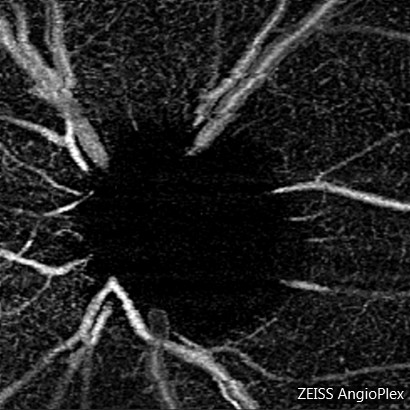

Supplement: S1 File — (ZIP) [file pone.0197588.s001.zip › Data Article Plos/Section application/Deep Network/Stade 3/20.jpg]

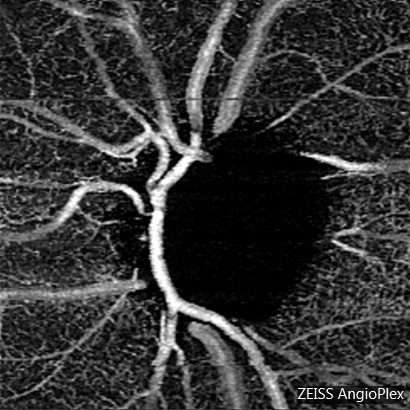

Supplement: S1 File — (ZIP) [file pone.0197588.s001.zip › Data Article Plos/Section application/Deep Network/Stade 3/3.jpg]

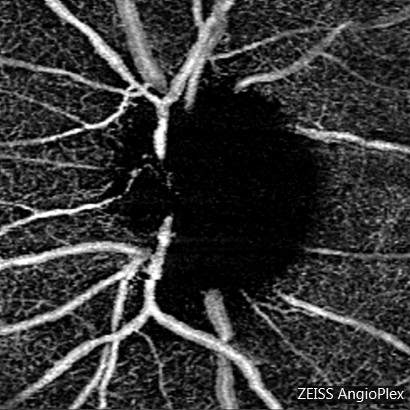

Supplement: S1 File — (ZIP) [file pone.0197588.s001.zip › Data Article Plos/Section application/Deep Network/Stade 3/5.jpg]

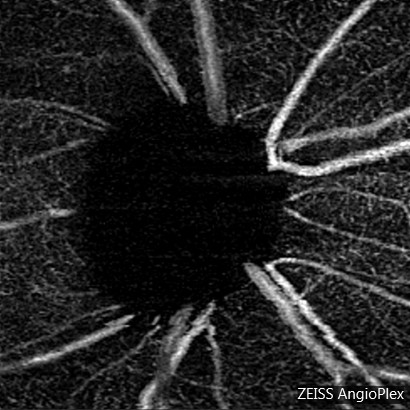

Supplement: S1 File — (ZIP) [file pone.0197588.s001.zip › Data Article Plos/Section application/Deep Network/Stade 3/6.jpg]

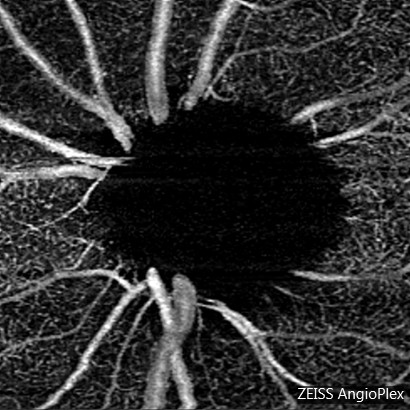

Supplement: S1 File — (ZIP) [file pone.0197588.s001.zip › Data Article Plos/Section application/Deep Network/Stade 3/7.jpg]

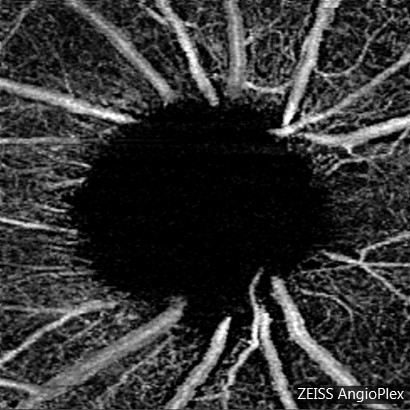

Supplement: S1 File — (ZIP) [file pone.0197588.s001.zip › Data Article Plos/Section application/Deep Network/Stade 3/8.jpg]

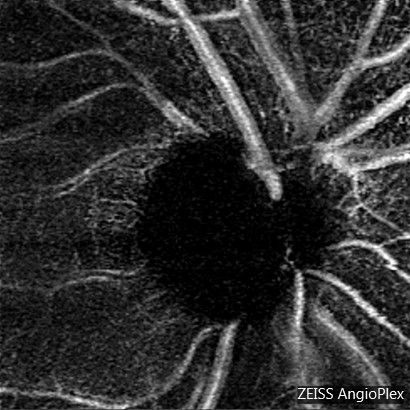

Supplement: S1 File — (ZIP) [file pone.0197588.s001.zip › Data Article Plos/Section application/Deep Network/Stade 3/9.jpg]

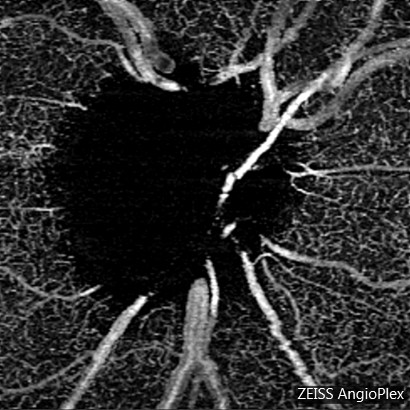

Supplement: S1 File — (ZIP) [file pone.0197588.s001.zip › Data Article Plos/Section application/Deep Network/Stade 4/1.jpg]

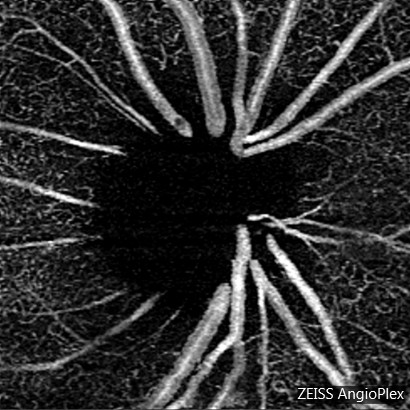

Supplement: S1 File — (ZIP) [file pone.0197588.s001.zip › Data Article Plos/Section application/Deep Network/Stade 4/10.jpg]

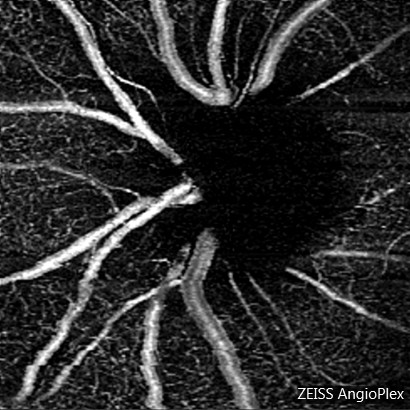

Supplement: S1 File — (ZIP) [file pone.0197588.s001.zip › Data Article Plos/Section application/Deep Network/Stade 4/11.jpg]

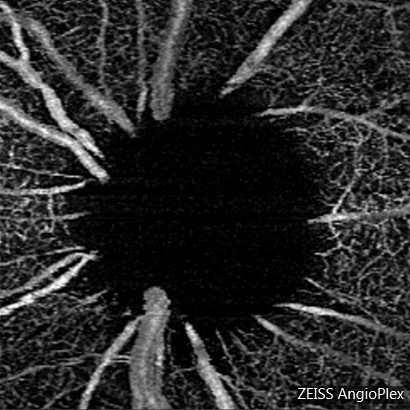

Supplement: S1 File — (ZIP) [file pone.0197588.s001.zip › Data Article Plos/Section application/Deep Network/Stade 4/12.jpg]

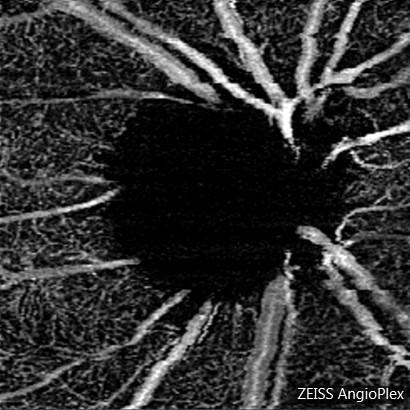

Supplement: S1 File — (ZIP) [file pone.0197588.s001.zip › Data Article Plos/Section application/Deep Network/Stade 4/13.jpg]

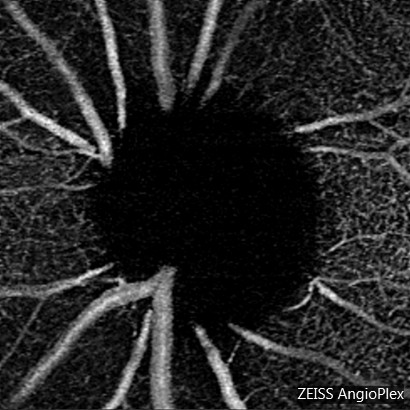

Supplement: S1 File — (ZIP) [file pone.0197588.s001.zip › Data Article Plos/Section application/Deep Network/Stade 4/14.jpg]

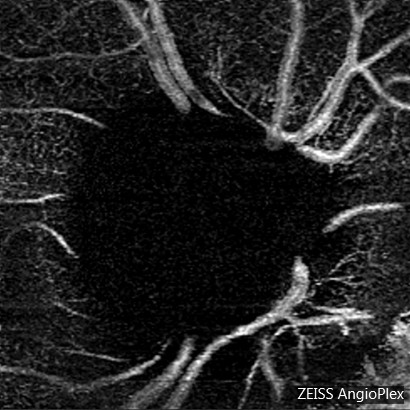

Supplement: S1 File — (ZIP) [file pone.0197588.s001.zip › Data Article Plos/Section application/Deep Network/Stade 4/15.jpg]

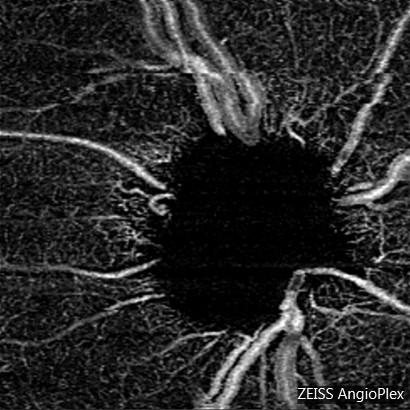

Supplement: S1 File — (ZIP) [file pone.0197588.s001.zip › Data Article Plos/Section application/Deep Network/Stade 4/17.jpg]

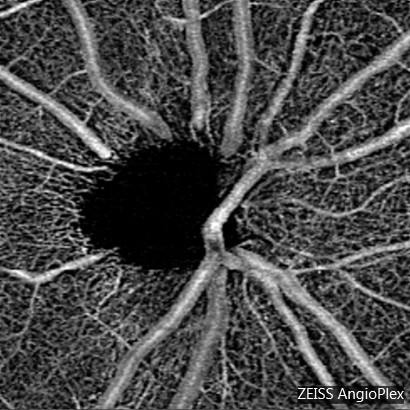

Supplement: S1 File — (ZIP) [file pone.0197588.s001.zip › Data Article Plos/Section application/Deep Network/Stade 4/18.jpg]

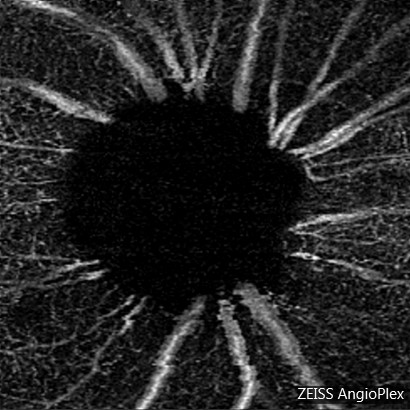

Supplement: S1 File — (ZIP) [file pone.0197588.s001.zip › Data Article Plos/Section application/Deep Network/Stade 4/19.jpg]

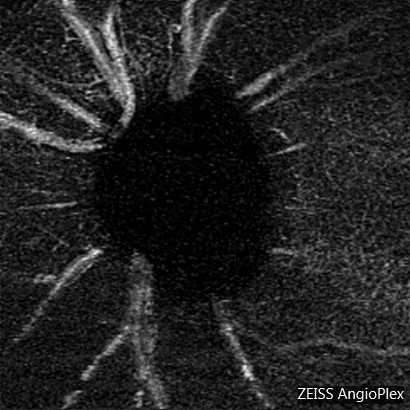

Supplement: S1 File — (ZIP) [file pone.0197588.s001.zip › Data Article Plos/Section application/Deep Network/Stade 4/2.jpg]

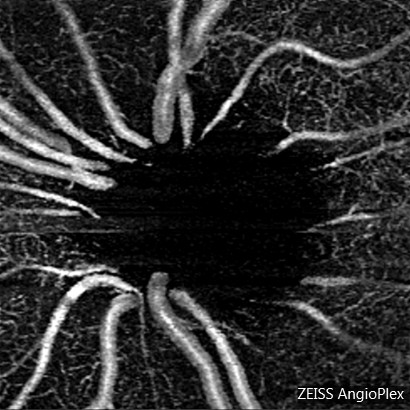

Supplement: S1 File — (ZIP) [file pone.0197588.s001.zip › Data Article Plos/Section application/Deep Network/Stade 4/3.jpg]

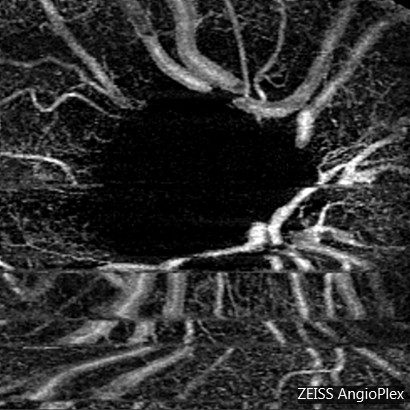

Supplement: S1 File — (ZIP) [file pone.0197588.s001.zip › Data Article Plos/Section application/Deep Network/Stade 4/4.jpg]

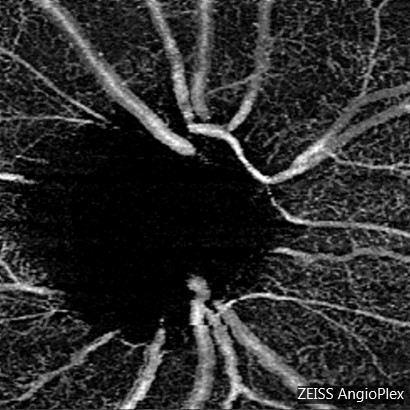

Supplement: S1 File — (ZIP) [file pone.0197588.s001.zip › Data Article Plos/Section application/Deep Network/Stade 4/5.jpg]

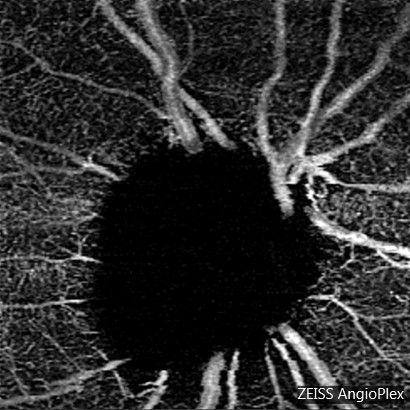

Supplement: S1 File — (ZIP) [file pone.0197588.s001.zip › Data Article Plos/Section application/Deep Network/Stade 4/7.jpg]

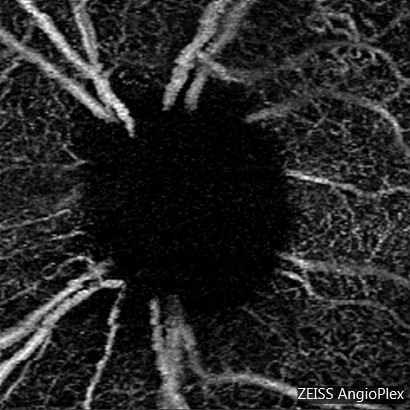

Supplement: S1 File — (ZIP) [file pone.0197588.s001.zip › Data Article Plos/Section application/Deep Network/Stade 4/8.jpg]

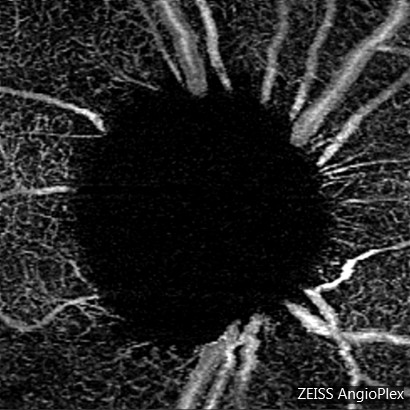

Supplement: S1 File — (ZIP) [file pone.0197588.s001.zip › Data Article Plos/Section application/Deep Network/Stade 4/9.jpg]

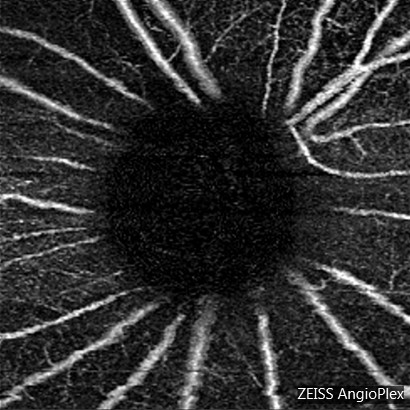

Supplement: S1 File — (ZIP) [file pone.0197588.s001.zip › Data Article Plos/Section application/Superficial network/ Stade 3/1.jpg]

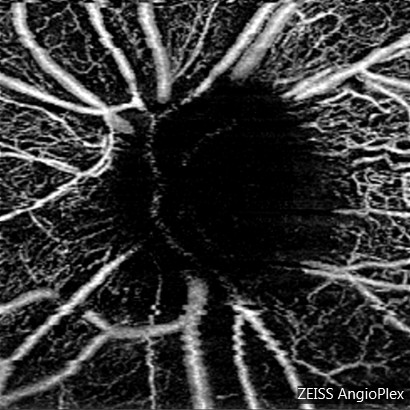

Supplement: S1 File — (ZIP) [file pone.0197588.s001.zip › Data Article Plos/Section application/Superficial network/ Stade 3/10.jpg]

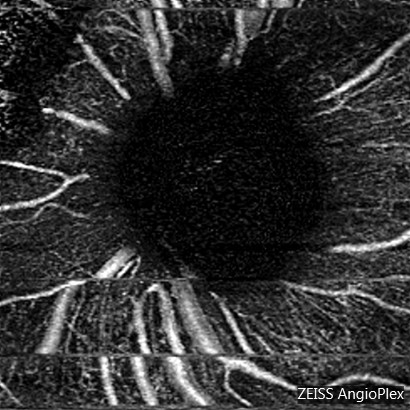

Supplement: S1 File — (ZIP) [file pone.0197588.s001.zip › Data Article Plos/Section application/Superficial network/ Stade 3/11.jpg]

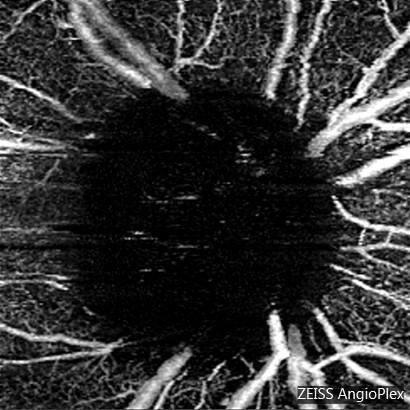

Supplement: S1 File — (ZIP) [file pone.0197588.s001.zip › Data Article Plos/Section application/Superficial network/ Stade 3/12.jpg]

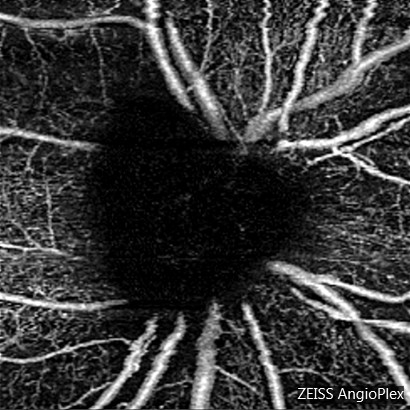

Supplement: S1 File — (ZIP) [file pone.0197588.s001.zip › Data Article Plos/Section application/Superficial network/ Stade 3/13.jpg]

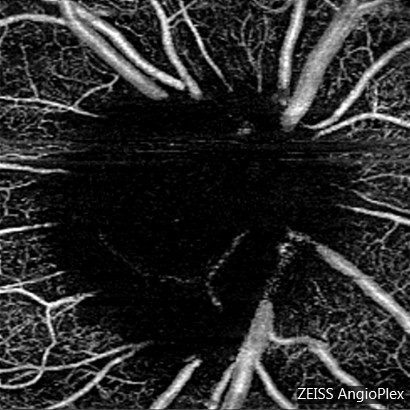

Supplement: S1 File — (ZIP) [file pone.0197588.s001.zip › Data Article Plos/Section application/Superficial network/ Stade 3/14.jpg]

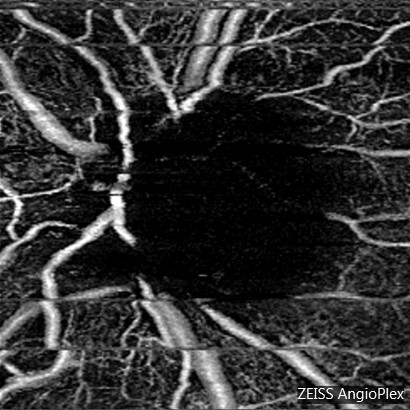

Supplement: S1 File — (ZIP) [file pone.0197588.s001.zip › Data Article Plos/Section application/Superficial network/ Stade 3/15.jpg]

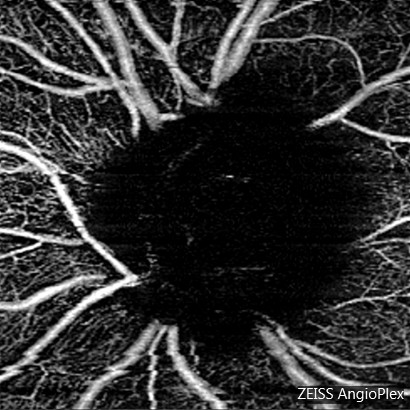

Supplement: S1 File — (ZIP) [file pone.0197588.s001.zip › Data Article Plos/Section application/Superficial network/ Stade 3/16.jpg]

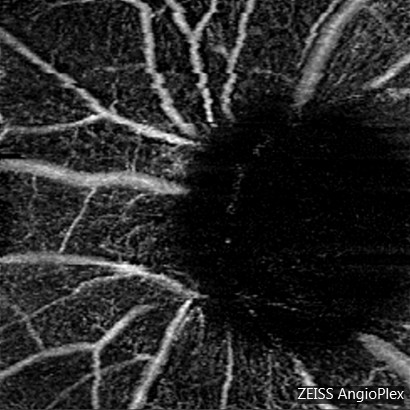

Supplement: S1 File — (ZIP) [file pone.0197588.s001.zip › Data Article Plos/Section application/Superficial network/ Stade 3/17.jpg]

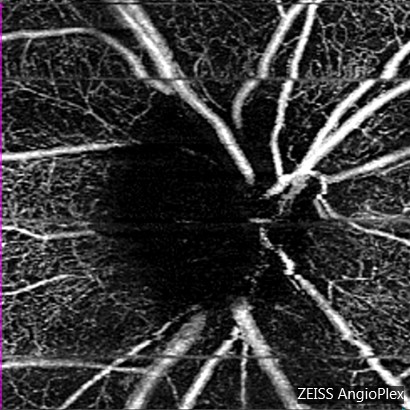

Supplement: S1 File — (ZIP) [file pone.0197588.s001.zip › Data Article Plos/Section application/Superficial network/ Stade 3/18.jpg]

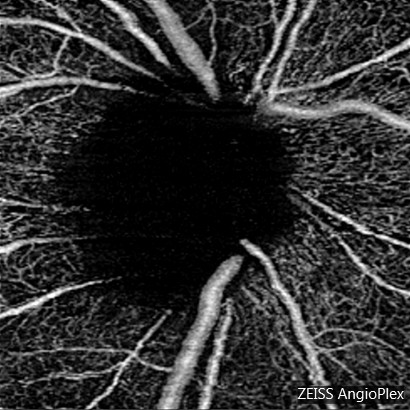

Supplement: S1 File — (ZIP) [file pone.0197588.s001.zip › Data Article Plos/Section application/Superficial network/ Stade 3/19.jpg]

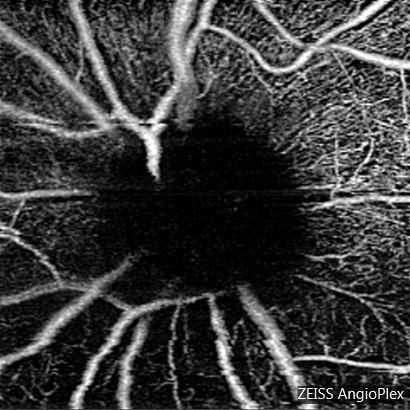

Supplement: S1 File — (ZIP) [file pone.0197588.s001.zip › Data Article Plos/Section application/Superficial network/ Stade 3/2.jpg]

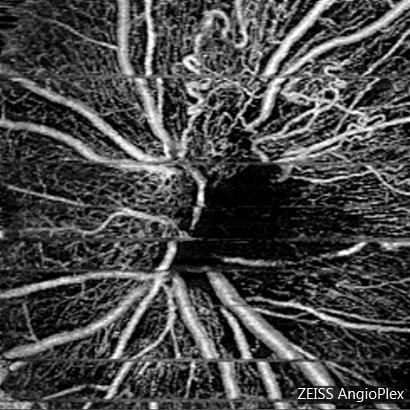

Supplement: S1 File — (ZIP) [file pone.0197588.s001.zip › Data Article Plos/Section application/Superficial network/ Stade 3/20.jpg]

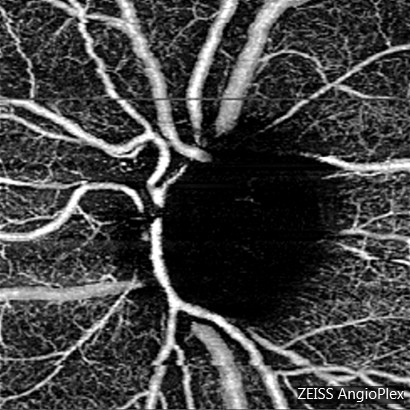

Supplement: S1 File — (ZIP) [file pone.0197588.s001.zip › Data Article Plos/Section application/Superficial network/ Stade 3/3.jpg]

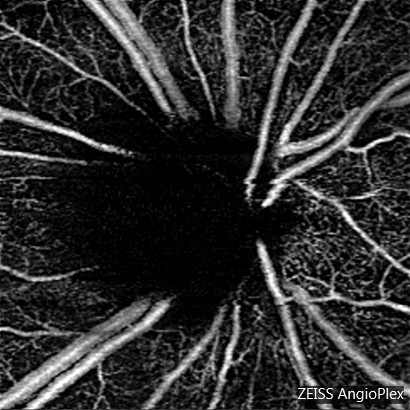

Supplement: S1 File — (ZIP) [file pone.0197588.s001.zip › Data Article Plos/Section application/Superficial network/ Stade 3/4.jpg]

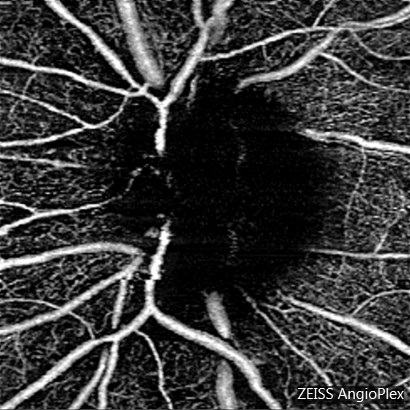

Supplement: S1 File — (ZIP) [file pone.0197588.s001.zip › Data Article Plos/Section application/Superficial network/ Stade 3/5.jpg]

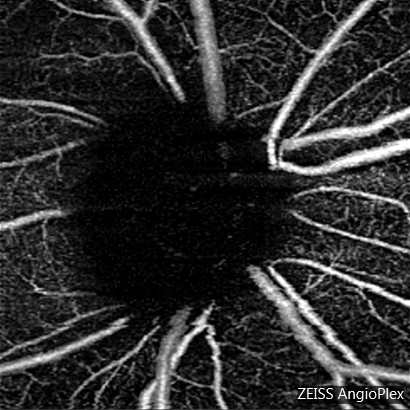

Supplement: S1 File — (ZIP) [file pone.0197588.s001.zip › Data Article Plos/Section application/Superficial network/ Stade 3/6.jpg]

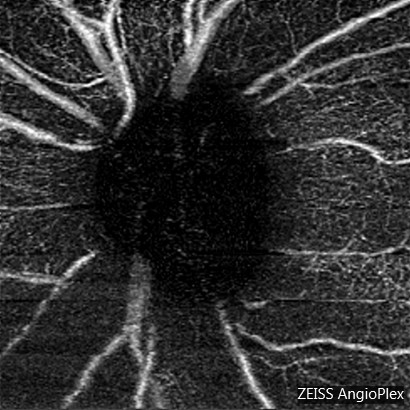

Supplement: S1 File — (ZIP) [file pone.0197588.s001.zip › Data Article Plos/Section application/Superficial network/ Stade 3/7.jpg]

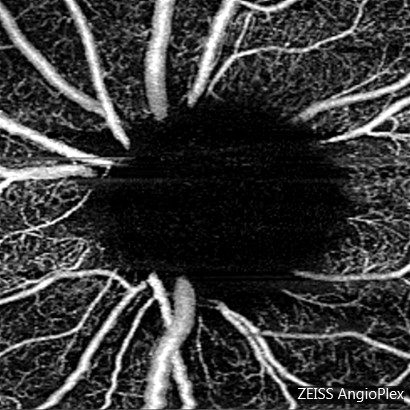

Supplement: S1 File — (ZIP) [file pone.0197588.s001.zip › Data Article Plos/Section application/Superficial network/ Stade 3/8.jpg]

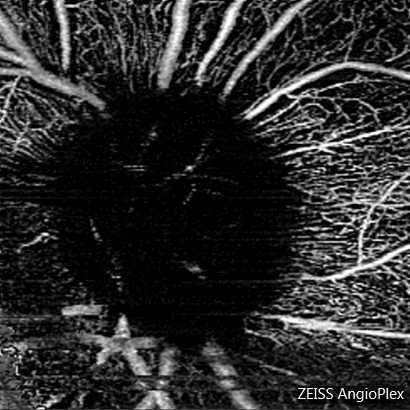

Supplement: S1 File — (ZIP) [file pone.0197588.s001.zip › Data Article Plos/Section application/Superficial network/ Stade 3/9.jpg]

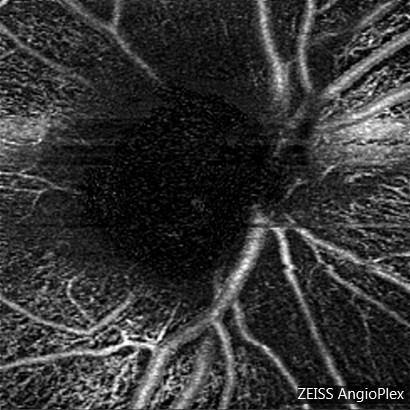

Supplement: S1 File — (ZIP) [file pone.0197588.s001.zip › Data Article Plos/Section application/Superficial network/Stade 1/1.jpg]

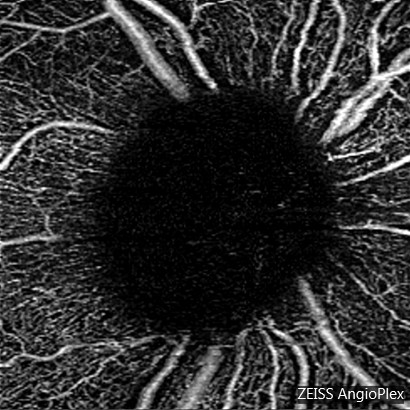

Supplement: S1 File — (ZIP) [file pone.0197588.s001.zip › Data Article Plos/Section application/Superficial network/Stade 1/10.jpg]

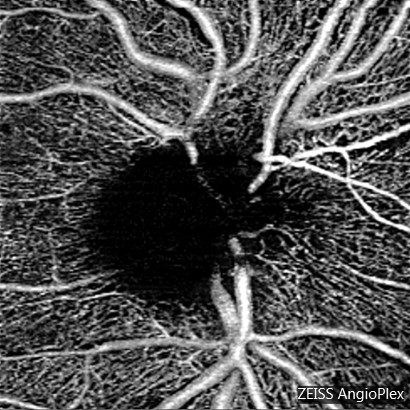

Supplement: S1 File — (ZIP) [file pone.0197588.s001.zip › Data Article Plos/Section application/Superficial network/Stade 1/11.jpg]

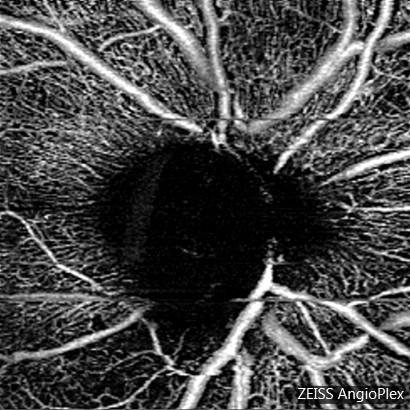

Supplement: S1 File — (ZIP) [file pone.0197588.s001.zip › Data Article Plos/Section application/Superficial network/Stade 1/12.jpg]

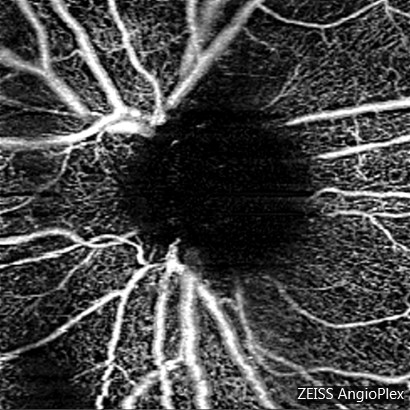

Supplement: S1 File — (ZIP) [file pone.0197588.s001.zip › Data Article Plos/Section application/Superficial network/Stade 1/13.jpg]
